# Selective arc-discharge synthesis of Dy_2_S-clusterfullerenes and their isomer-dependent single molecule magnetism†
†Electronic supplementary information (ESI) available. CCDC 1546957 and 1551313. For ESI and crystallographic data in CIF or other electronic format see DOI: 10.1039/c7sc02395b


**DOI:** 10.1039/c7sc02395b

**Published:** 2017-06-30

**Authors:** Chia-Hsiang Chen, Denis S. Krylov, Stanislav M. Avdoshenko, Fupin Liu, Lukas Spree, Ravi Yadav, Antonis Alvertis, Liviu Hozoi, Konstantin Nenkov, Aram Kostanyan, Thomas Greber, Anja U. B. Wolter, Alexey A. Popov

**Affiliations:** a Leibniz Institute for Solid State and Materials Research Dresden , 01069 Dresden , Germany . Email: a.popov@ifw-dresden.de; b Physik-Institut , Universität Zürich , Zürich , Switzerland

## Abstract

A method for the selective synthesis of sulfide clusterfullerenes Dy_2_S@C_2*n*_ is developed. Addition of methane to the reactive atmosphere reduces the formation of empty fullerenes in the arc-discharge synthesis, whereas the use of Dy_2_S_3_ as a source of metal and sulfur affords sulfide clusterfullerenes as the main fullerene products along with smaller amounts of carbide clusterfullerenes. Two isomers of Dy_2_S@C_82_ with *C*_s_(6) and *C*_3v_(8) cage symmetry, Dy_2_S@C_72_-*C*_s_(10528), and a carbide clusterfullerene Dy_2_C_2_@C_82_-*C*_s_(6) were isolated. The molecular structure of both Dy_2_S@C_82_ isomers was elucidated by single-crystal X-ray diffraction. SQUID magnetometry demonstrates that all of these clusterfullerenes exhibit hysteresis of magnetization, with Dy_2_S@C_82_-*C*_3v_(8) being the strongest single molecule magnet in the series. DC- and AC-susceptibility measurements were used to determine magnetization relaxation times in the temperature range from 1.6 K to 70 K. Unprecedented magnetization relaxation dynamics with three consequent Orbach processes and energy barriers of 10.5, 48, and 1232 K are determined for Dy_2_S@C_82_-*C*_3v_(8). Dy_2_S@C_82_-*C*_s_(6) exhibits faster relaxation of magnetization with two barriers of 15.2 and 523 K. *Ab initio* calculations were used to interpret experimental data and compare the Dy-sulfide clusterfullerenes to other Dy-clusterfullerenes. The smallest and largest barriers are ascribed to the exchange/dipolar barrier and relaxation *via* crystal-field states, respectively, whereas an intermediate energy barrier of 48 K in Dy_2_S@C_82_-*C*_3v_(8) is assigned to the local phonon mode, corresponding to the librational motion of the Dy_2_S cluster inside the carbon cage.

## Abstract 2

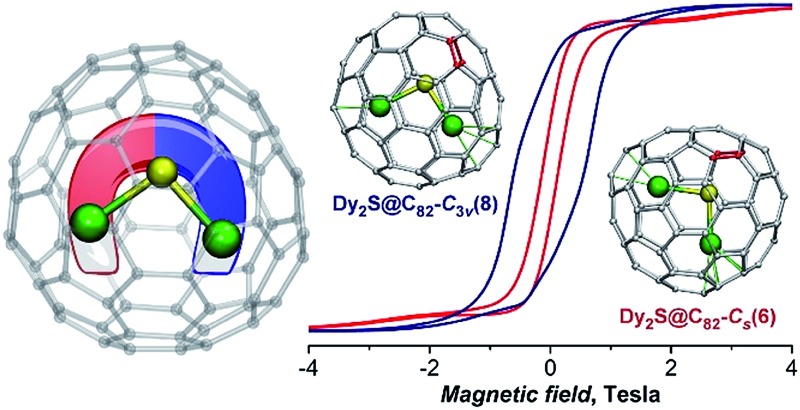
Dy-sulfide clusterfullerene single molecule magnets are synthesized selectively, and their relaxation of magnetization is thoroughly studied by DC and AC-magnetometry.

## Introduction

The discovery of single molecule magnetism in the Mn_12_ complex in 1993 (ref. 1) initiated an on-going chase for molecules with a high blocking temperature and large relaxation barrier of magnetization. Lanthanides entered the field in 2003 with the report on the slow relaxation of magnetization in their double–decker complexes,^2^ and hundreds of lanthanide-SMMs have been described since that time.^3^–^12^ Quantum tunneling of magnetization (QTM) is one of the most important mechanisms of losing the spin information by single-ion lanthanide SMMs at zero field. One of the ways to improve the situation is to increase the local symmetry of the crystal field acting on the lanthanide ion, and some of the best single-ion Dy-SMM have been obtained following this strategy.^10^,^13^–^15^ Another approach is to combine two or more lanthanide ions in one molecule,^5^,^16^–^19^ or combine lanthanides with transition metals in 3d–4f complexes.^20^–^25^ Exchange and dipolar coupling in polynuclear complexes create a manifold of additional states and an additional barrier to relaxation, thus preventing QTM. Therefore, single-ion anisotropy and inter-lanthanide interactions are the two key ingredients in improving the SMM properties.

Endohedral metallofullerenes (EMFs),^26^,^27^ and in particular clusterfullerenes,^28^ combining lanthanides and non-metal ions in endohedral species, provide a convenient platform for creating SMMs. The presence of negatively charged non-metal ions (such as a nitride ion N^3–^ in the nitride clusterfullerene M_3_N@C_80_) close to the lanthanide ions leads to the large magnetic anisotropy of the latter,^29^–^33^ whereas the possibility of varying the composition of the endohedral cluster by combining lanthanides with scandium or other diamagnetic analogs allows tuning the intracluster interactions.^34^–^36^ Both parameters can change strongly with variation of the central (non-metal) atom in clusterfullerenes. EMFs emerged as a new class of SMMs in 2012, when the nitride clusterfullerene DySc_2_N@C_80_ was shown to exhibit a hysteresis of magnetization with zero-field quantum tunneling of magnetization.^37^ Subsequent studies have shown that SMM behavior of Dy–Sc nitride clusterfullerenes strongly depends on the endohedral cluster composition, with Dy_2_ScN@C_80_ being a better SMM than DySc_2_N@C_80_ and much better than Dy_3_N@C_80_.^38^ The superior SMM properties of Dy_2_ScN@C_80_ are explained by the ferromagnetic exchange and dipolar coupling of Dy ions, which lead to the exchange/dipolar barrier of 10.5 K and suppress zero-field QTM. At higher temperatures, the relaxation of the magnetization in Dy_2_ScN@C_80_ proceeds *via* the Orbach mechanism with a high barrier of 1735 K, corresponding to the fifth Kramers doublet of the Dy^3+^ ion.^39^ The long magnetization relaxation time of Dy_2_ScN@C_80_ was partially preserved even on a metallic substrate.^40^ HoSc_2_N@C_80_ was also found to be a SMM, albeit with much faster relaxation than in the Dy analog.^41^ Other types of clusterfullerenes were also tested for SMM behavior. Ti-carbide Dy_2_TiC@C_80_ was found to exhibit hysteresis similar to Dy_2_ScN@C_80_, albeit with a lower blocking temperature.^42^ At the same time, addition of one more carbon atom to the cluster, such as in Dy_2_TiC_2_@C_80_, led to substantially worsened SMM properties.^42^ Field-induced SMM behavior was also demonstrated for cyano-clusterfullerenes with single metal atoms, TbNC@C_82_ (ref. 43) and TbNC@C_76_.^44^ As Tb^3+^ and Ho^3+^ are non-Kramers ions, the corresponding EMFs exhibit much faster relaxation of magnetization, and hence better EMF-SMMs are to be looked for among Dy-EMFs.

In this work we focus on Dy-based sulfide clusterfullerenes of the formula Dy_2_S@C_2*n*_ to study how Dy–S bonding and inter-lanthanide coupling *via* the sulfide bridge affect the SMM properties. The first synthesis of the sulfide clusterfullerene Sc_2_S@C_82_ was reported in 2010.^45^ In that work, guanidinium thiocyanate was used as a source of nitrogen in the synthesis of nitride clusterfullerenes, and the sulfide was obtained as a by-product with much lower relative yield. Echegoyen *et al.* used the addition of SO_2_ gas to the reactor atmosphere and obtained a family of Sc_2_S@C_2*n*_ EMFs with 2*n* ranging from 70 to 100 according to mass-spectrometry.^46^ In SO_2_-assisted synthesis, empty fullerenes are the main fullerene products. Thus, both synthetic routes to sulfide clusterfullerenes led to other types of fullerenes (nitride clusterfullerenes or empty fullerenes) as the main products. Isolation of sulfide clusterfullerenes then required tedious multistep chromatographic separation. The principal possibility to obtain non-Sc M_2_S@C_82_ clusterfullerenes was also demonstrated in 2010, but the isolated amounts were very small.^45^

The low selectivity of the arc-discharge synthesis is a serious obstacle when low-yield EMFs, such as sulfide clusterfullerenes, are the goal of the synthesis. It is therefore desirable to develop more selective approaches for the synthesis of clusterfullerenes. The first selective method for the synthesis of EMFs was developed by Dunsch and coworkers.^47^,^48^ The authors found that addition of NH_3_ gas to the arc-discharge reactor atmosphere dramatically reduced the yield of empty fullerenes but did not affect the formation of nitride clusterfullerenes. The latter could be thus obtained with a high degree of selectivity. High selectivity of nitride clusterfullerene formation was also achieved with the use of solid nitrogen sources (such as guanidinium thiocyanate,^49^ inorganic salts,^50^ melamine,^51^ or urea^52^) or using NO_*x*_ vapor from NO_*x*_-generating solid reagents and air (known as the CAPTEAR approach).^53^ More recently, we have adapted a method for selective synthesis of carbide clusterfullerenes using methane as a reactive gas.^42^,^54^–^57^ Its influence on the arc-discharge is similar to that of NH_3_. Namely, hydrogen suppresses the formation of empty fullerenes, and carbide clusterfullerenes, especially Ti-carbide clusterfullerenes M_2_TiC@C_80_ and M_2_TiC_2_@C_80_,^42^,^55^ as well as Sc-carbide Sc_3_CH@C_80_ (ref. 54) and Sc_4_C_2_@C_80_,^55^ can be obtained with a high degree of selectivity.

In this work, we pursue two goals. First, we develop the procedure for the selective synthesis of sulfide clusterfullerenes and synthesize a new family of EMF-SMMs, Dy-based sulfide clusterfullerenes. Second, we perform a thorough analysis of the magnetic properties of the Dy-sulfide clusterfullerenes and demonstrate that they exhibit SMM behavior. Their unprecedented magnetization relaxation dynamics is analyzed as a function of temperature and the main relaxation pathways are revealed.

## Results and discussion

### Synthesis of clusterfullerenes

**Fig. 1:**
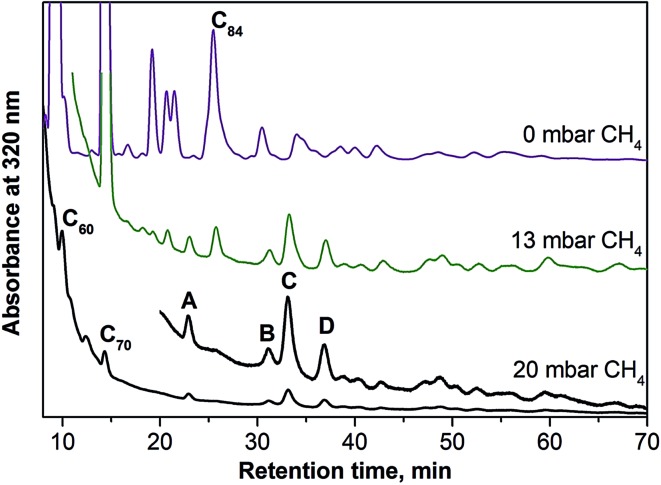
HPLC traces of the extracts obtained in the arc-discharge synthesis with Dy_2_S_3_ and different amounts of methane in the reactor atmosphere. Block letters in the lower trace denote the main clusterfullerene fractions, corresponding to (A) Dy_2_S@C_72_, (B) Dy_3_N@C_80_, (C) Dy_2_S@C_82_-I/Dy_2_C_2_@C_82_-I, and (D) Dy_2_S@C_82_-II. Note that a different intensity is used for each curve.

Selective synthesis of nitride and carbide clusterfullerenes was achieved *via* addition of hydrogen-containing compounds. Hydrogen suppresses the yield of empty fullerenes, and EMFs can be obtained with improved selectivity. To achieve a similar effect in the synthesis of sulfide clusterfullerenes, several combinations of dysprosium and sulfur sources were tested. In particular, we used (i) a mixture of Dy powder with elementary sulfur; (ii) a mixture of Dy powder with a solid organic sulfur compound, dibenzyl sulfide; (iii) Dy_2_S_3_ sulfide. The syntheses were performed in pure helium atmosphere (230 mbar), as well as with the addition of several mbar of methane. Mass-spectrometric analysis showed that all three strategies led to formation of Dy_2_S@C_2*n*_ clusterfullerenes, albeit with quite a low yield. In all cases the presence of methane increased the relative yield of sulfide clusterfullerenes (as far as it could be decided based on laser-desorption ionization time-of flight (LDI-TOF) mass-spectra; note that conclusions on the yield of EMFs based on LDI-TOF data should be treated with caution). The best results were obtained with the use of Dy_2_S_3_ as a simultaneous source of metal and sulfur, and this route was then further optimized by varying the amount of methane. Fig. 1 compares HPLC traces of CS_2_ fullerene extracts obtained in the syntheses without methane and with 13 mbar and 20 mbar of CH_4_ (total pressure was kept at 250 mbar). In the absence of methane, empty fullerenes are formed in much larger amounts than EMFs, and the HPLC trace is very similar to that of a standard empty fullerene synthesis (not shown). Addition of 13 mbar CH_4_ to the reactor atmosphere immediately reduced the yield of empty fullerenes, and EMF peaks with retention times longer than 30 minutes can be well seen in the chromatogram. Their intensities are comparable to those of higher empty fullerenes (the yield of C_60_ and C_70_ is still considerably higher). In the presence of 20 mbar CH_4_, formation of empty fullerenes is suppressed almost completely, leaving only several well established peaks marked with block letters (A–D). Mass-spectral analysis revealed that each peak corresponds to clusterfullerenes, including Dy_2_S@C_72_ (A), Dy_3_N@C_80_ (B), and two isomers of Dy_2_S@C_82_ (C and D). Mass-spectra of the fraction collected at longer retention times (37–45 min) also showed the presence of Dy_2_S@C_78_ and Dy_2_S@C_86_, but their amounts are too low for separation. Formation of the nitride clusterfullerene Dy_3_N@C_80_ seems to be inevitable even when only traces of nitrogen are present in the generator (earlier we observed the same effect in the synthesis of carbide clusterfullerenes^42^). Mass-spectral analysis of the fractions C and D also showed that they contained certain amounts of carbide clusterfullerenes Dy_2_C_2_@C_82_. To obtain pure compounds, recycling HPLC was used at the second separation step (Fig. S1†). As a result of the chromatographic separation, pure Dy_2_S@C_72_, two isomers of Dy_2_S@C_82_, and one isomer of Dy_2_C_2_@C_82_ were obtained. It should be noted that the use of methane suppresses the formation of empty fullerenes and simplifies the separation of sulfide clusterfullerenes, but their overall yield remains quite low. The isolated amounts for each compound were less than 1 mg.

### Spectroscopic characterization and molecular structures

**Fig. 2:**
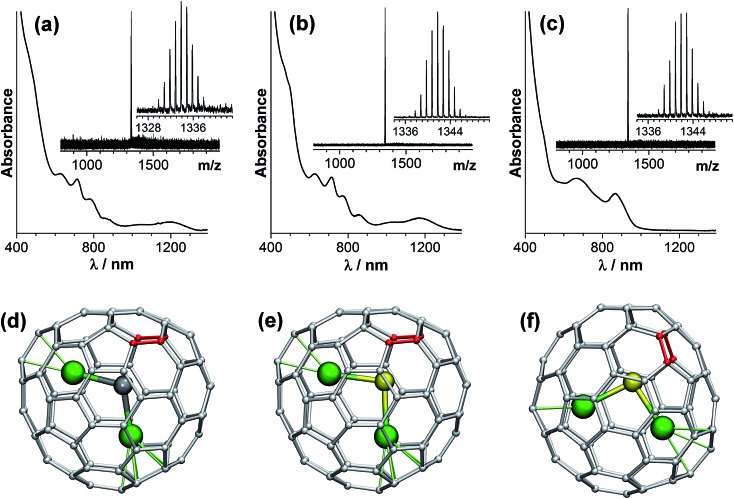
(a–c) UV-vis-NIR absorption spectra of Dy-clusterfullerenes in toluene: (a) Dy_2_C_2_@C_82_-*C*_s_(6), (b) Dy_2_S@C_82_-*C*_s_(6), and (c) Dy_2_S@C_82_-*C*_3v_(8); insets show the LDI-TOF mass-spectra for each clusterfullerene (positive ion mode). (d–f) DFT-optimized molecular structures of (d) Y_2_C_2_@C_82_-*C*_s_(6), (e) Y_2_S@C_82_-*C*_s_(6) and (f) Y_2_S@C_82_-*C*_3v_(8); Y atoms are green, S atoms are yellow, and carbon atoms are light gray; C_82_-*C*_s_(6) and C_82_-*C*_3v_(8) cages are related *via* Stones–Wales transformations of the two C–C bonds highlighted in red.

Molecular structures of the isolated clusterfullerenes are first established with the use of UV-vis-NIR absorption spectroscopy. Due to multiple π–π* transitions, the absorption spectra of EMFs are very sensitive to the isomeric structure of the fullerene cage, which can be used for structure elucidation. Fig. 2 shows that Dy_2_S@C_82_-I and Dy_2_C_2_@C_82_-I have very similar absorption spectra, which indicates that these two EMFs have the same carbon cage. This spectral pattern is in fact very characteristic for the EMFs with a C_82_-*C*_s_(6) cage in the formal four-fold charged state, including Er_2_S@C_82_-*C*_s_(6),^58^ Sc_2_C_2_@C_82_-*C*_s_(6),^59^ Sc_2_S@C_82_-*C*_s_(6),^46^ and Y_2_C_2_@C_82_-*C*_s_(6).^60^ Thus, we can reliably assign the cage isomer of isostructural Dy_2_S@C_82_ and Dy_2_C_2_@C_82_ molecules as C_82_-*C*_s_(6). The absorption pattern of Dy_2_S@C_82_-II closely resembles that of EMFs with the C_82_-*C*_3v_(8) cage in the four-fold charged state, such as sulfide clusterfullerenes Er_2_S@C_82_-*C*_3v_(8)^58^ and Sc_2_S@C_82_-*C*_3v_(8),^45^,^46^ or the carbide clusterfullerene M_2_C_2_@C_82_-*C*_3v_(8).^60^,^61^ Note that the C_82_-*C*_s_(6) and C_82_-*C*_3v_(8) cages are rather similar and are related *via* two Stone–Wales transformations (*i.e. via* the pseudo-rotation of two C–C bonds highlighted in red in Fig. 2 by 90°).

**Fig. 3:**
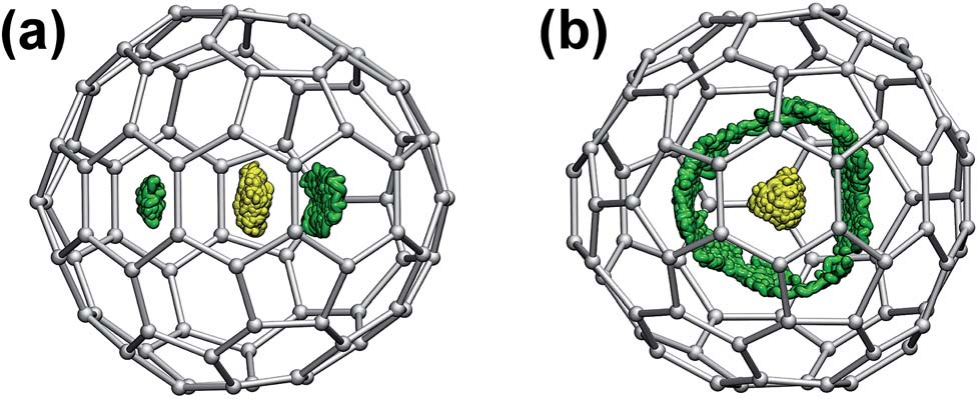
(a, b) Born–Oppenheimer molecular dynamics simulations of (a) Y_2_S@C_82_-*C*_s_(6) and (b) Y_2_S@C_82_-*C*_3v_(8) at the PBE/DZVP level, *T* = 300 K, propagation time 100 ps. Displacements of carbon atoms are not shown. In (a), the symmetry plane of the C_82_-*C*_s_(6) cage is perpendicular to the paper (in Fig. 2e, the plane is parallel to the paper). In (b), the molecule is viewed along the C_3_ axis of the C_82_-*C*_3v_(8) cage (which lies in the plane of the paper in Fig. 2f).

Possible orientations of the endohedral clusters in the Dy_2_C_2_@C_82_ and Dy_2_S@C_82_ isomers are addressed with the use of DFT calculations (Fig. 2d–f). To avoid difficulties of treating the system with partially-filled 4f-shells at the DFT level, we used Y as a model of Dy in such calculations because of their close ionic radius. For Y_2_C_2_@C_82_-*C*_s_(6) and Y_2_S@C_82_-*C*_s_(6), our calculations revealed one particular cluster orientation (identical for both carbide and sulfide clusters), which is at least 25 kJ mol^–1^ lower in energy than all other configurations (Fig. 2d and e). For Y_2_S@C_82_-*C*_3v_(8), the calculations revealed several energy minima, all related *via* rotation of the cluster around the *C*_3_ axis of the carbon cage; the lowest-energy one is shown in Fig. 2f. DFT-based Born–Oppenheimer molecular dynamics (BOMD) simulations for Y_2_S@C_82_-*C*_s_(6) at 300 and 450 K did not reveal reorientation of the cluster on the 100 ps time scale (Fig. 3a). These data indicate that the Dy_2_S cluster in Dy_2_S@C_82_-*C*_s_(6) is probably fixed, or exhibits jump-like rotations with a low rate. Note that NMR studies of Sc_2_C_2_@C_82_-*C*_s_(6) revealed that the rotation of the cluster became significant at the NMR time-scale only at temperatures above 370 K.^59^ For Y_2_S@C_82_-*C*_3v_(8), BOMD simulations show that at room temperature the cluster rotates around the *C*_3_ axis (Fig. 3b). A similar conclusion on the rotation of the Sc_2_S cluster was drawn earlier for Sc_2_S@C_82_-*C*_3v_(8).^45^

**Fig. 4:**
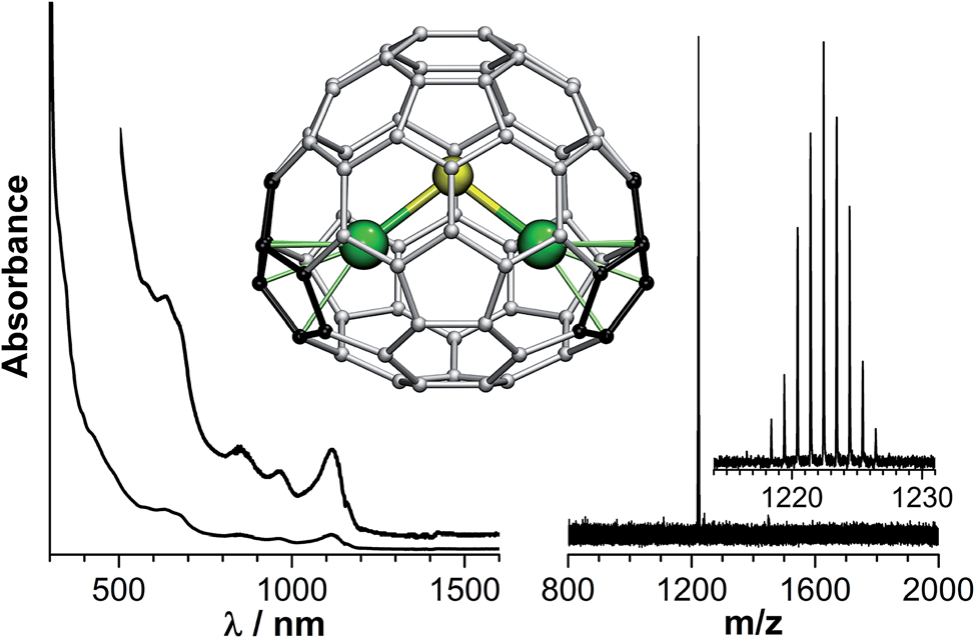
UV-vis-NIR absorption spectrum (left) and positive-ion LDI mass-spectrum (right) of Dy_2_S@C_72_. The molecular structure of Dy_2_S@C_72_-*C*_s_(10528) is shown in the middle, and adjacent pentagon pairs are highlighted in black.

Assignment of the structure of Dy_2_S@C_72_ is based on the close similarity of its absorption spectrum (Fig. 4) to that of the non-IPR Sc_2_S@C_72_-*C*_s_(10528) reported by Echegoyen *et al.*^62^ DFT calculations of different cage isomers of Y_2_S@C_72_ also show that C_72_-*C*_s_(10528) is the most energetically favorable cage isomer for Y_2_S@C_72_ (see ESI†). The second most stable isomer, Y_2_S@C_72_-*C*_s_(10616), is 42 kJ mol^–1^ less stable. Thus, based on the absorption spectra and DFT calculations, we assign the structure of isolated Dy_2_S@C_72_ to the non-IPR C_72_-*C*_s_(10528) cage isomer. In this structure, the metal atoms are coordinated to adjacent pentagon pairs, and the cluster is tightly fixed inside the fullerene.

### Single-crystal X-ray diffraction

**Fig. 5:**
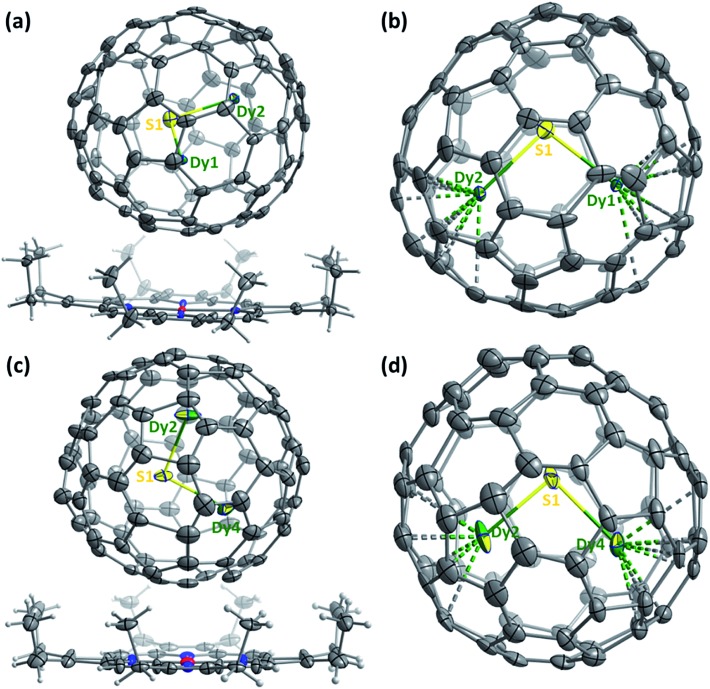
(a) Relative orientation of the Ni^II^(OEP) and Dy_2_S@C_82_ molecules in the Dy_2_S@C_82_-*C*_s_(6)·Ni^II^(OEP)·2C_7_H_8_ cocrystal; only one orientation of the C_82_-*C*_s_(6) cage together with the major site of the Dy_2_S cluster are shown, solvent molecules are omitted for clarity; (b) major site of the Dy_2_S cluster within the *C*_s_(6)-C_82_ cage. Selected geometry parameters: Dy1–S1, 2.465(5) Å; Dy2–S1, 2.518(5) Å; Dy1–S1–Dy2, 98.3(2)°. (c) Relative orientation of the Ni^II^(OEP) and Dy_2_S@C_82_ molecules in the Dy_2_S@C_82_-*C*_3v_(8)·Ni^II^(OEP)·2C_7_H_8_ cocrystal; only one orientation of the C_82_-*C*_3v_(8) cage together with the major site of the Dy_2_S cluster are shown, solvent molecules are omitted for clarity; (d) major site of the Dy_2_S cluster within the C_82_-*C*_3v_(8) cage. Selected geometry parameters: Dy2–S1, 2.437(11) Å; Dy4–S1, 2.511(9) Å; Dy2–S1–Dy4, 94.4(2)°. Displacement parameters are shown at the 30% probability level.

The molecular structures of Dy_2_S@C_82_-*C*_s_(6) and Dy_2_S@C_82_-*C*_3v_(8) are further corroborated by single-crystal X-ray diffraction of the cocrystal Dy_2_S@C_82_·Ni^II^(OEP)·2C_7_H_8_ (Fig. 5), obtained by layering a toluene solution of Ni^II^(OEP) (OEP = octaethylporphyrin) over a CS_2_ solution of the fullerene following the procedure developed in ref. 63. After the two solutions diffused together over a period of one month, small black crystals suitable for X-ray crystallographic study formed. X-ray diffraction data collection for the crystal was carried out at 100 K at the BESSY storage ring (BL14.3, Berlin-Adlershof, Germany)^64^ using a MAR225 CCD detector, *λ* = 0.89429 Å. Processing the diffraction data was done with the XDSAPP2.0 suite.^65^ The structure was solved by direct methods and refined using all data (based on *F*^2^) by SHELX 2016.^66^ Hydrogen atoms were located in a difference map, added geometrically, and refined with a riding model. The data can be obtained free of charge from The Cambridge Crystallographic Data Centre with CCDC No. 1546957 and 1551313.†

The asymmetric unit cells of both crystals contain a half of the Ni^II^(OEP) molecule and two halves of the C_82_-*C*_s_(6) or C_82_-*C*_3v_(8) cage. The fully ordered Ni^II^(OEP) molecule is perpendicular to the crystal mirror plane, so the intact molecule was generated by combining the existing half-molecule with its mirror image. Complete fullerene cages in both crystals were generated by combining one of the halves of the fullerene cage with the mirror image of the other. Accordingly, the occupancies of the two cage orientations in both crystals are 0.50 and 0.50, respectively.

For Dy_2_S@C_82_-*C*_s_(6), two symmetry-related sulfur positions with 0.50/0.50 occupancies were refined. Dy is disordered over 9 sites with occupancies of 2 × 0.35 (Dy1), 2 × 0.34 (Dy2), 0.11 (Dy3), 0.11 (Dy4), 2 × 0.09 (Dy5), and 0.23 (Dy6) (Dy1, Dy2, and Dy5 are located in general positions and their sites are duplicated by the crystallographic mirror plane, see ESI† for details). The major configuration of the cluster shown in Fig. 5b (Dy1–S–Dy2, 68% of all Dy_2_S sites) corresponds to the lowest energy structure found by DFT (Fig. 2e) and is similar to that in the crystal structure of Sc_2_S@C_82_-*C*_s_(6).^67^

For Dy_2_S@C_82_-*C*_3v_(8), S is disordered over 3 sites with occupancies of 2 × 0.31 (S1) and 0.38 (S2). Dy is disordered over 12 sites with occupancies of 0.25 (Dy1), 0.38 (Dy2), 2 × 0.24 (Dy3), 2 × 0.18 (Dy4), 2 × 0.16 (Dy5), 2 × 0.04 (Dy6), and 2 × 0.07 (Dy7) (S1, Dy3, Dy4, Dy5, Dy6 and Dy7 are located in general positions and their sites are duplicated by the crystallographic mirror plane, see ESI† for details). Two configurations of the cluster (Dy2–S1–Dy4 shown in Fig. 5d, and Dy1–S1–Dy3) covering 61% of all Dy_2_S sites have the same orientation of the cluster inside the cage as in the lowest energy conformer found by DFT (Fig. 2f). This configuration is also similar to the major site (60%) of the Sc_2_S cluster in the co-crystal of Sc_2_S@C_82_-*C*_3v_(8) with Ni^II^(OEP).^67^

### Magnetic properties of Dy-clusterfullerenes

**Fig. 6:**
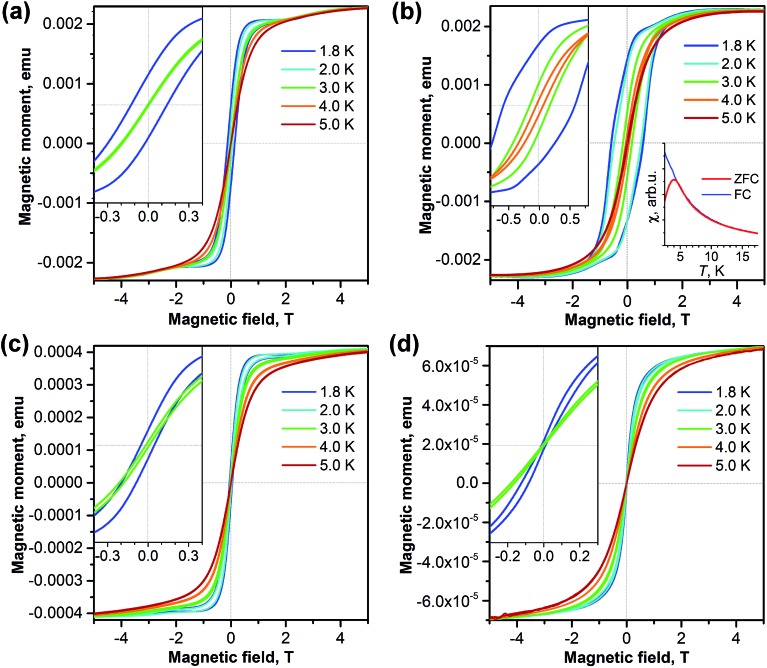
Magnetization curves for (a) Dy_2_S@C_82_-*C*_s_(6), (b) Dy_2_S@C_82_-*C*_3v_(8), (c) Dy_2_C_2_@C_82_-*C*_s_(6), and (d) Dy_2_S@C_72_-*C*_s_(10528) measured at *T* = 1.8–5 K with the magnetic field sweep rate of 8.33 mT s^–1^. The inset in each panel zooms into the region near zero-field. In (b), determination of the blocking temperature of Dy_2_S@C_82_-*C*_3v_(8) as the peak in the susceptibility of the zero-field-cooled (ZFC) sample is also shown (magnetic field 0.2 T, temperature sweep rate 5 K min^–1^).

The isolation of two isomers of Dy_2_S@C_82_ and the isomer of the carbide Dy_2_C_2_@C_82_ with the same carbon cage as one of the sulfide clusterfullerenes allows us to address the question of how the carbon cage and type of internal cluster affect the magnetic properties of EMFs. Fig. 6 shows magnetization curves for each sample measured in the temperature range from 1.8 to 5 K. Quite remarkable is the difference between the two isomers of Dy_2_S@C_82_. The *C*_s_ isomer exhibits narrow hysteresis at 1.8 K (coercive field 0.12 T), which closes at 3 K. The hysteresis of the *C*_3v_ isomer is significantly broader at 1.8 K (coercive field 0.58 T, Fig. 6b), and the closing temperature is between 4 and 5 K. For Dy_2_S@C_82_-*C*_3v_ we could also measure the blocking temperature *T*_B_ = 4 K as the temperature of the peak in the susceptibility of the zero-field-cooled (ZFC) sample (Fig. 6b); for other samples the *T*_B_ values are near 2 K, which is too low to be reliably measured. Another SMM characteristic, the temperature at which the relaxation time of magnetization is 100 s, is determined for Dy_2_S@C_82_-*C*_3v_ to be *T*_B100_ = 2 K.

The magnetization behavior of Dy_2_C_2_@C_82_-*C*_s_ is similar to that of the isostructural sulfide. The hysteresis is narrower but closes at a slightly higher temperature (Fig. 6c). Finally, Dy_2_S@C_72_ has the smallest opening of hysteresis among all studied samples (Fig. 6d). Thus, all four studied clusterfullerenes exhibited hysteresis of magnetization below 3 K and can be classified as single molecule magnets. Importantly, we observe considerably different SMM properties of sulfide clusterfullerenes with different fullerene cages.

### Dynamics of the relaxation of magnetization

**Fig. 7:**
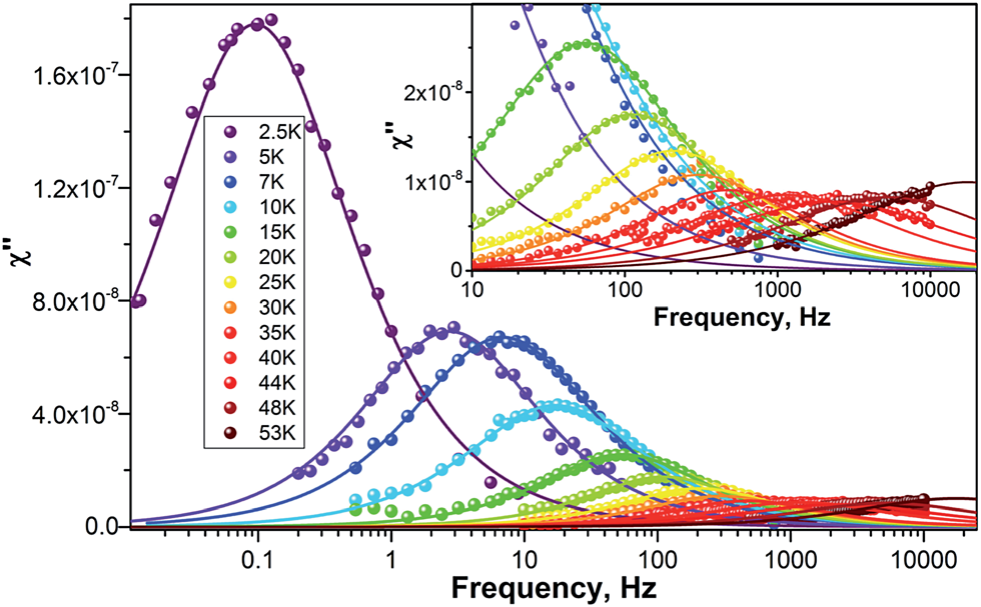
*χ*″ of Dy_2_S@C_82_-*C*_s_ measured at different temperatures as a function of AC frequency. Dots are experimental points, lines are results of the fit with a generalized Debye model.

To study the dynamics of the relaxation of magnetization at temperatures up to 60–70 K, we performed AC-susceptibility measurements for Dy_2_C_2_@C_82_ and the two isomers of Dy_2_S@C_82_ (the amount of isolated Dy_2_S@C_72_ was not sufficient for such measurements). Characteristic temperature-dependent peaks in the out-of-phase susceptibility were found for all samples. As an example, Fig. 7 shows *χ*″ susceptibility for Dy_2_S@C_82_-*C*_s_; analogous data for other compounds are available in the ESI.† Magnetization relaxation times *τ* shorter than 10 s were determined from the AC-data using a generalized Debye model (see Cole–Cole plots in the ESI†). The longer *τ* values at the lowest temperatures were determined directly by measuring the relaxation of magnetization in a DC mode.

**Fig. 8:**
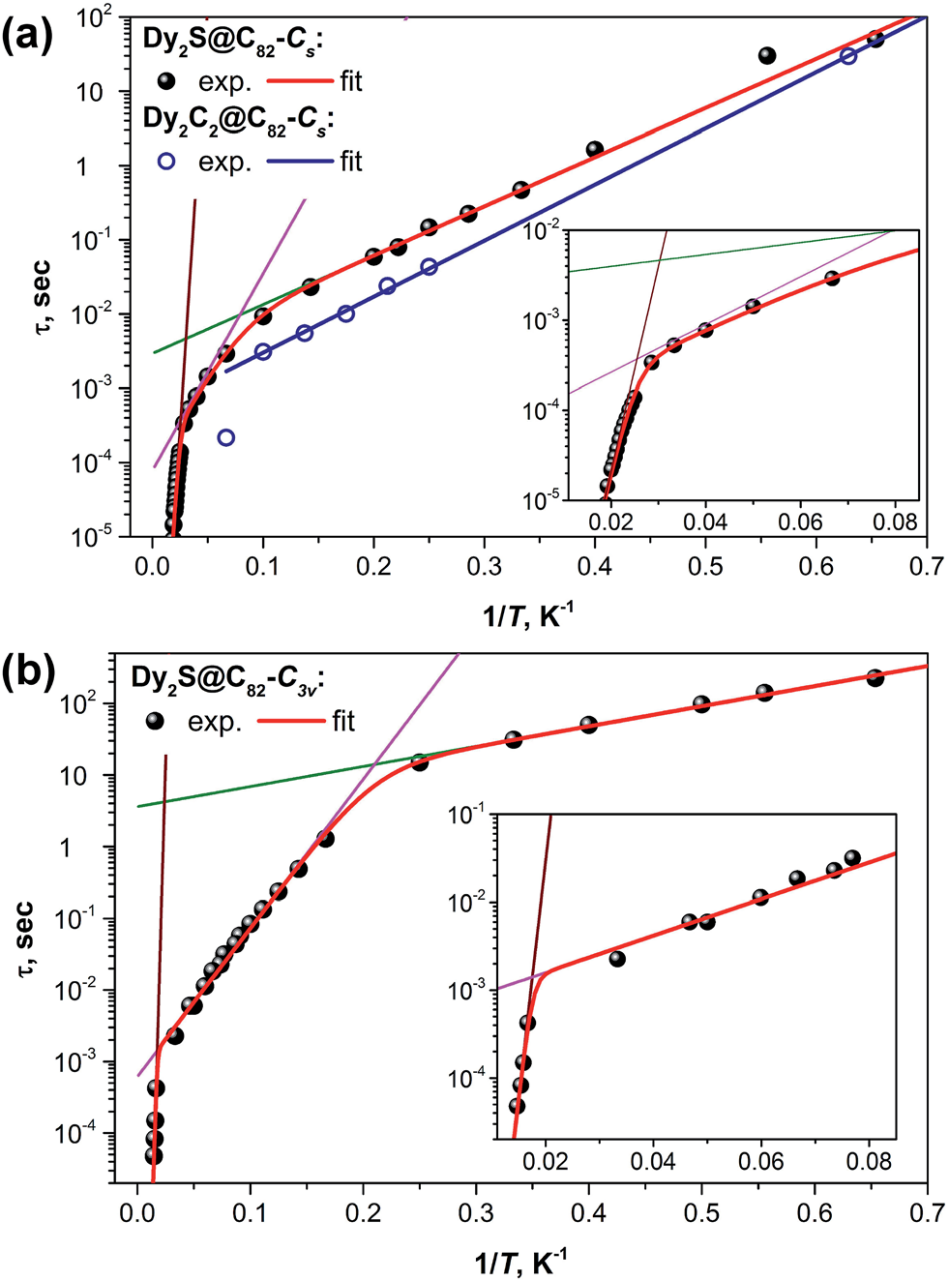
Magnetization relaxation times of (a) Dy_2_C_2_@C_82_-*C*_s_ and Dy_2_S@C_82_-*C*_s_ and (b) Dy_2_S@C_82_-*C*_3v_. Dots are experimental points, red lines are results of a global fit with three Orbach processes; green, magenta, and brown lines represent contributions of individual Orbach processes. For Dy_2_C_2_@C_82_-*C*_s_ with a limited number of data points, a single Orbach process was considered (blue line). Insets show enhancement of the high-temperature range for Dy_2_S@C_82_-*C*_s_ and Dy_2_S@C_82_-*C*_3v_. Fitting of the magnetization relaxation of Dy_2_S@C_82_-*C*_s_ with two Orbach processes and one Raman process is shown in the ESI.†

Fig. 8 shows the plots of magnetization relaxation times of Dy_2_S@C_82_-*C*_s_, Dy_2_C_2_@C_82_-*C*_s_, and Dy_2_S@C_82_-*C*_3v_ as a function of reciprocal temperature. The two isomers of Dy_2_S@C_82_ exhibit strikingly different relaxation dynamics, which in both cases can be described as a combination of Orbach relaxation processes *via* two or three thermal barriers. The relaxation rate for the Orbach relaxation mechanism is the exponential function of the reciprocal temperature and the energy of an excited state, which defines the effective relaxation barrier *U*^eff^:

1 *τ*_Orbach_^–1^ = *τ*_0_^–1^ exp(–*U*^eff^/*T*)

In the log(*τ*) *vs.* 1/*T* coordinates, relaxation *via* the Orbach mechanism appears as a straight line. A combination of several Orbach relaxation processes and relaxation *via* the Raman mechanism would be then described by the equation:2
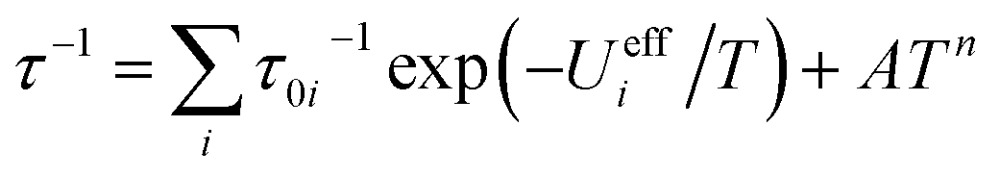
where index *i* runs through all processes, and the term *AT*^*n*^ describes the rate of the relaxation *via* a Raman mechanism. Table 1 lists *U*eff*i*, *τ*_0*i*_, and other parameters determined by fitting experimental relaxation times by eqn (2). For Dy_2_S@C_82_-*C*_3v_, the best fit is obtained with three Orbach processes. For Dy_2_C_2_@C_82_-*C*_s_, the limited set of data allowed only fitting with a single Orbach process. For Dy_2_S@C_82_-*C*_s_, equally good fits were obtained with either three Orbach processes, or two Orbach processes and a Raman relaxation (see ESI†). Observation of several Orbach relaxation pathways is rather unusual but not unimaginable. Two linear regimes in the temperature dependence of relaxation rates have been observed for several 3d–4f SMMs. In these complexes, the low-temperature process corresponds to the relaxation *via* exchange excited states, whereas the higher-energy barrier is due to the Orbach relaxation *via* the crystal-field excited state of the lanthanide ions.^23^,^68^,^69^

**Table 1:** Parameters of Orbach and Raman relaxation processes in Dy-EMFs and the temperature range where these processes play the main role in the relaxation of the magnetization^*a*^

	Dy_2_C_2_@C_82_-*C*_s_	Dy_2_S@C_82_-*C*_s_^*b*^	Dy_2_S@C_82_-*C*_s_^*c*^	Dy_2_S@C_82_-*C*_3v_	Dy_2_ScN@C_80_-*I*_h_^*d*^
*U* eff 1	17.4 ± 0.2	15.2 ± 0.3	18.0 ± 0.5	6.5 ± 0.5	10.7 ± 0.3
*τ*_01_	(5.2 ± 0.3) × 10^–4^	(2.9 ± 0.3) × 10^–3^	(1.6 ± 0.2) × 10^–3^	3.6 ± 0.8	11.9 ± 1.5
*T*_range_	1.6–10	1.6–10	1.8–15	1.6–4	1.8–5
*U* eff 2		61 ± 6		48 ± 1	
*τ*_02_		(7.8 ± 2.3) × 10^–5^		(6.2 ± 0.6) × 10^–4^	
*T*_range_		15–35		5–47	
*A*			(2.5 ± 0.6) × 10^–3^		
*n*			3.97 ± 0.08		
*T*_range_			≤1.6, 20–43		
*U* eff 3		523 ± 35	696 ± 86	1232 ± 160	1735 ± 21
*τ*_03_		(6.0 ± 4.4) × 10^–10^	(2.5 ± 4.3) × 10^–11^	(0.6 ± 1.5) × 10^–12^	(2.4 ± 0.8) × 10^–15^
*T*_range_ ^*e*^		40–53	47–53	47–70	63–76

^*a*^Effective barriers *U*eff*i* are given in Kelvin, *τ*_0*i*_ values are given in seconds.

^*b*^Modelling magnetization relaxation rate of Dy_2_S@C_82_-*C*_s_ with three Orbach processes.

^*c*^Modelling magnetization relaxation rate of Dy_2_S@C_82_-*C*_s_ with two Orbach processes and an intermediate Raman process; for Dy_2_S@C_82_-*C*_3v_, the Raman relaxation mechanism could not give an acceptable fit (see ESI).

^*d*^From ref. 39.

^*e*^The highest temperature of the range is determined by the frequency and sensitivity limits of the PPMS system.

At the lowest accessible temperatures (1.6–5 K), all three Dy-EMFs exhibit a linear regime with a relatively small barrier, *U*eff1, presumably corresponding to the energy difference between the states with ferromagnetically and antiferromagnetically coupled Dy ions (it has contributions from both dipolar and exchange interactions, see more detailed discussion below). Magnetization relaxation pathways proceeding through excited “exchange states” are well documented for 3d–4f complexes, albeit usually with much shorter *τ*_0_ values than those observed in EMFs.^20^,^21^,^23^,^68^–^71^ In Dy_2_S@C_82_-*C*_3v_, the *U*eff1 barrier amounts to 6.5 K *versus* 15.2 K in Dy_2_S@C_82_-*C*_s_ and 17.4 K in Dy_2_C_2_@C_82_-*C*_s_. At the same time, Dy_2_S@C_82_-*C*_3v_ has the longest attempt time *τ*_01_ of 3.6 s, which is 3–4 orders of magnitude longer than that of the EMFs with the *C*_s_ cage isomer (2.9 ms in Dy_2_S@C_82_-*C*_s_ and 0.5 ms in Dy_2_C_2_@C_82_-*C*_s_). Thus, due to the smaller barrier, the *C*_3v_ isomer has a moderate inclination in log(*τ*) *vs.* 1/*T* and hence smaller variation of the relaxation rate with temperature, whereas its much longer *τ*_01_ value leads to the considerably longer magnetization relaxation times. The difference between the two isomers of Dy_2_S@C_82_ reaches two orders of magnitude near 5 K. In due turn, the magnetization of Dy_2_C_2_@C_82_-*C*_s_ relaxes *ca.* two times faster than that of the isostructural Dy_2_S@C_82_-*C*_s_ showing that the acetylide C_2_^2–^ central unit in the Dy_2_C_2_ cluster is inferior for the SMM properties compared to the sulfide ion S^2–^ in the Dy_2_S cluster within the same fullerene cage. This finding agrees with our earlier study of Dy_2_TiC@C_80_ and Dy_2_TiC_2_@C_80_, which also showed that the single carbide ion in the endohedral cluster leads to much better SMMs than the C_2_ unit.^42^ The best EMF-SMM molecule so far is the nitride clusterfullerene Dy_2_ScN@C_80_-*I*_h_. It also has a *U*eff1 barrier of 10.5 K and a long *τ*_01_ value of 12 s (see Table 1).^39^ Thus, the comparison between sulfide, carbide, and nitride clusterfullerenes with two Dy atoms shows that their magnetization relaxation dynamics at low temperatures is determined by the Orbach process with the “exchange” barrier. The height of the barrier appears to be less important than the attempt time, which varies by several orders of magnitude between the EMFs. The best SMM in the series is not the EMF with the largest exchange barrier, but the molecule with the longest *τ*_01_ value.

Above 5 K, the magnetization relaxation mechanisms for the *C*_3v_ and *C*_s_ isomers become significantly different. Between 5 and 47 K, the magnetization relaxation of Dy_2_S@C_82_-*C*_3v_ is driven by another Orbach process with *U*eff2 = 48 K and *τ*_02_ = 0.36 ms. As will be discussed below in more detail, this barrier is too small to be assigned to one of the crystal-field (CF) states, and the *τ*_02_ value is likewise too long for the Orbach processes *via* CF states normally observed for Dy-SMMs. Above 47 K and up to the instrumental frequency limit at 70 K, the magnetization relaxation of Dy_2_S@C_82_-*C*_3v_ is determined by the energy barrier of 1232 K and the corresponding *τ*_03_ value of 0.6 × 10^–12^ s. Unfortunately, the measurements in this temperature range and frequencies, with the small amount of the available sample, are performed near the sensitivity limit of the PPMS system, which leads to large uncertainties in the determined values. Yet, there is no doubt that the barrier is rather high, but smaller than the barrier of the analogous relaxation process in Dy_2_ScN@C_80_, 1735 K. For comparison, the highest thermal relaxation barrier among lanthanide-only dimers, 721 K, was reported recently by Gao *et al.* for hydroxide-bridged five-coordinate Dy^III^ dimer.^18^ The highest barrier among non-fullerene polynuclear Dy complexes is 888 K,^72^ whereas in single-ion Dy SMMs, the largest reported barrier is 1815 K.^13^

For the *C*_s_ isomers, the linear regime with the “exchange” barrier is operative up to *ca.* 10–15 K. Above *ca.* 45 K, Dy_2_S@C_82_-*C*_s_ exhibits a high-energy Orbach process with the parameters typical for the relaxation *via* a CF state, *i.e.* large *U*eff3 of several hundred K and a *τ*_03_ value in the range of 10^–10^–10^–11^ s. However, the relaxation dynamics between the temperature ranges of the two Orbach processes, *i.e.* 15–40 K, are not uniquely defined. Equally good fits were obtained for either an intermediate Orbach process (*U*eff2 = 61 K and *τ*_02_ = 0.08 ms; see Fig. 8a) or for the Raman relaxation process (*A* = 2.5 ms K^–*n*^ and *n* = 3.97, see Fig. S20†). The choice of either an Orbach or Raman process also affects the parameters of other Orbach processes, especially the *U*eff3 and *τ*_03_ values (Table 1). The high energy Orbach process is observed at AC frequencies close to the frequency and sensitivity limits of the instrument, which significantly affects the accuracy of the fit and leads to large uncertainties for the *U*eff3 and *τ*_03_ values. It is very likely that the third linear regime for Dy_2_S@C_82_-*C*_s_ is not fully reached at accessible temperatures, and that the actual energy barrier for the relaxation *via* CF state is higher.

To summarize, although Dy_2_S@C_82_-*C*_3v_ and Dy_2_S@C_82_-*C*_s_ have a similar structure of the encapsulated Dy_2_S cluster, the differences in their fullerene cages have paramount effect on the magnetization relaxation dynamics. In the whole temperature range accessible for our measurements, relaxation times of the *C*_3v_ isomer are considerably longer than those of the *C*_s_ isomer, from a factor of 5 to two orders of magnitude. The difference in the relaxation behavior of Dy_2_S@C_82_-*C*_s_ and Dy_2_C_2_@C_82_-*C*_s_ is not as pronounced as between the isomers of Dy_2_S@C_82_, which shows that the influence of the cage isomerism may be stronger than the influence of the central atom(s) in the endohedral clusters.

Few SMMs with sulfur-ligated Dy have been reported so far,^73^–^75^ and all of them have substantially faster relaxation times and smaller relaxation barriers than in the Dy_2_S@C_82_ system reported in this work. In the EMF molecules, sulfur bears a substantially larger negative charge and the Dy–S sulfur distances are at the same time much shorter, which altogether leads to a stronger crystal field in sulfide clusterfullerenes.

### Effective spin Hamiltonian for di-nuclear Dy EMFs

The system with two Dy centers with magnetic moments *J*_1,2_ weakly coupled through exchange/dipolar interactions can be described by the following effective spin Hamiltonian:

3 *Ĥ*_tot_ = *Ĥ*_CF1_ + *Ĥ*_CF2_ – 2*j*_12_*Ĵ*_1_*Ĵ*_2_

where the *Ĥ*_CF*i*_ terms are single-ion crystal-field Hamiltonians, and the last term describes the exchange and dipolar interactions between the two Dy centers (rather unfortunately, both the exchange coupling and total magnetic moment of lanthanide are traditionally designated as *J*, so we use the small letter *j* for the coupling and the capital *J* for the momentum). In the spirit of the Lines model, both exchange and dipolar interactions are modelled here by a single isotropic coupling parameter *j*_12_. We will first describe *ab initio* computations for the single-ion CF parameters in sulfide clusterfullerenes and compare them to other EMFs, then proceed to the discussion of the coupling parameter *j*_12_, and then comment on the spectrum of the total effective spin Hamiltonian.

### *Ab initio* calculations of single-ion magnetic anisotropy in Dy-EMFs

Single-point *ab initio* calculations discussed in this section were performed using a complete active space self-consistent field with spin–orbit interactions (CASSCF/SO-RASSI level of theory) as implemented in MOLCAS 8.0.^76^ In all systems, Dy^3+^ has a ^6^H_15/2_ ground state multiplet, resulting in eight low-lying Kramers doublets. The active space of the CASSCF calculations includes nine active electrons and seven active orbitals (*e.g.* CAS (9,7)). The single ion CF-parameters were then obtained with the use of the SINGLE_ANISO module^77^ and transferred to the PHI code^78^ for further pseudospin analysis of single ion as well total spin states. The crystal structures of EMFs often exhibit strong disorder of the cage and cluster positions, thus limiting the use of X-ray determined geometries for accurate analysis of the crystal field splitting. Besides, crystal structures are not always available. In this work, molecular geometries were optimized by DFT for Y analogs, and then one of the Y ions was replaced by Dy for the subsequent *ab initio* calculations. Table 2 lists the CF energy levels for each Dy ion in Dy_2_S@C_72_, the two isomers of Dy_2_S@C_82_, and Dy_2_C_2_@C_82_-*C*_s_. Note that the term “crystal field” is somewhat ambiguous here since *de facto* we discuss splitting of the *J*_z_ levels by the intramolecular interactions between the Dy ion and surrounding ions. The term “crystal field” is inherited in the field of SMMs from the earlier studies of lanthanide solids and broadly used in the literature, and we follow this convention.

**Table 2:** The energies of the single-ion crystal-field states (Kramers doublets, KD) in Dy clusterfullerenes^*a*^

KD	Dy_2_S@C_72_	Dy_2_C_2_@C_82_-*C*_s_		Dy_2_S@C_82_-*C*_s_		Dy_2_S@C_82_-*C*_3v_	
		Dy1	Dy2	Dy1	Dy2	Dy1	Dy2
1	0	0	0	0	0	0	0
2	181	225	185	228	221	269	295
3	398	354	393	381	450	424	459
4	572	469	551	512	622	549	593
5	658	588	668	648	723	653	701
6	691	688	747	764	799	743	788
7	765	742	791	848	857	806	858
8	876	806	868	913	905	881	967
*d*(Dy–X^*b*^), Å	2.456	2.313	2.329	2.484	2.506	2.509	2.486
∠(Dy–X^*b*^–Dy)°	104.7	112.1		99.4		97.2	

^*a*^Energies are given in cm^–1^, the conversion factor of cm^–1^ to Kelvin units is 1.439.

^*b*^X is either a sulfide ion in sulfide clusterfullerenes or a center of the acetylide unit in Dy_2_C_2_@C_82_.

The ground states of the Dy ions in all studied EMFs feature a highly anisotropic *g*-tensor with *g*_*zz*_ near 19.8–19.9 and vanishingly small *g*_*xx*_ and *g*_*yy*_ components (see ESI† for more details), which corresponds to the “pure” state with *J*_*z*_ = ±15/2. The overall CF splitting (Δ*E*_1–8_ hereafter) is in the range of 810–970 cm^–1^. The smallest energy difference between the ground and the first excited state, Δ*E*_1–2_ = 181 cm^–1^, is found in Dy_2_S@C_72_; for all other EMFs the Δ*E*_1–2_ energies are larger and reach 295 cm^–1^ for one of the Dy centers in Dy_2_S@C_82_-*C*_3v_. These values are sufficiently high to conclude that the magnetic properties of these EMFs at liquid helium temperatures are determined solely by the ground state and intramolecular exchange/dipolar coupling between magnetic moments.

Although the central non-metal ion is the main “source” of magnetic anisotropy, the CF splitting in sulfide clusterfullerenes is not a simple function of the metal-sulfur distance. With a considerably shorter Dy–S distance, Dy_2_S@C_72_ has the smallest Δ*E*_1–2_ energy gap among the studied sulfide clusterfullerenes. Likewise, with almost identical Dy–S bond lengths and cluster geometry, the Δ*E*_1–2_ values in Dy_2_S@C_82_-*C*_3v_ are larger than those in Dy_2_S@C_82_-*C*_s_. Thus, despite the relatively small charges of individual carbon atoms, the fullerene cage (and in particular, the coordination mode of Dy ions to the nearby carbons) also plays a certain role, which may have a critical effect on the difference between the otherwise similar isomers.

The influence of the non-metal species on the magnetic anisotropy can be clearly seen from the comparison of Dy_2_C_2_@C_82_-*C*_s_ and Dy_2_S@C_82_-*C*_s_. The Dy atoms in both molecules have virtually identical metal-cage coordination. Besides, the orientations of the anisotropy axes for each metal center are also very similar (along the Dy–S axes in Dy_2_S@C_82_ and along the axes connecting Dy and the midpoint between the two carbons in Dy_2_C_2_@C_82_). Finally, the acetylide unit and the sulfide ion have the same formal charge, –2. But in the Dy_2_S cluster, the negative charge is localized on the single sulfur atom, whereas in the Dy_2_C_2_ cluster the charge is shared between the two carbons. As a result, the CF splitting in the carbide clusterfullerene is systematically smaller than that in the sulfide clusterfullerene by 10%.

To place these results into a broader context, we performed *ab initio* calculations for Dy centers in other di-Dy clusterfullerenes known to exhibit SMM properties, including Dy_2_ScN@C_80_-*I*_h_, Dy_2_TiC@C_80_-*I*_h_, and Dy_2_TiC_2_@C_80_-*I*_h_. Also studied were hypothetical DyNC@C_82_-*C*_2_(5), DyNC@C_76_-*C*_2v_(19138), and Dy_2_O@C_82_-*C*_3v_(8), whose synthesis appears feasible based on the literature reports on analogous EMFs with other metals (such as cyano clusterfullerenes TbNC@C_76_ (ref. 44) and three cage isomers of TbNC@C_82_,^43^,^79^ or oxide clusterfullerenes Sc_2_O@C_2*n*_,^80^–^82^ Y_2_O@C_2*n*_, and Lu_2_O@C_2*n*_ (ref. 83)).

**Fig. 9:**
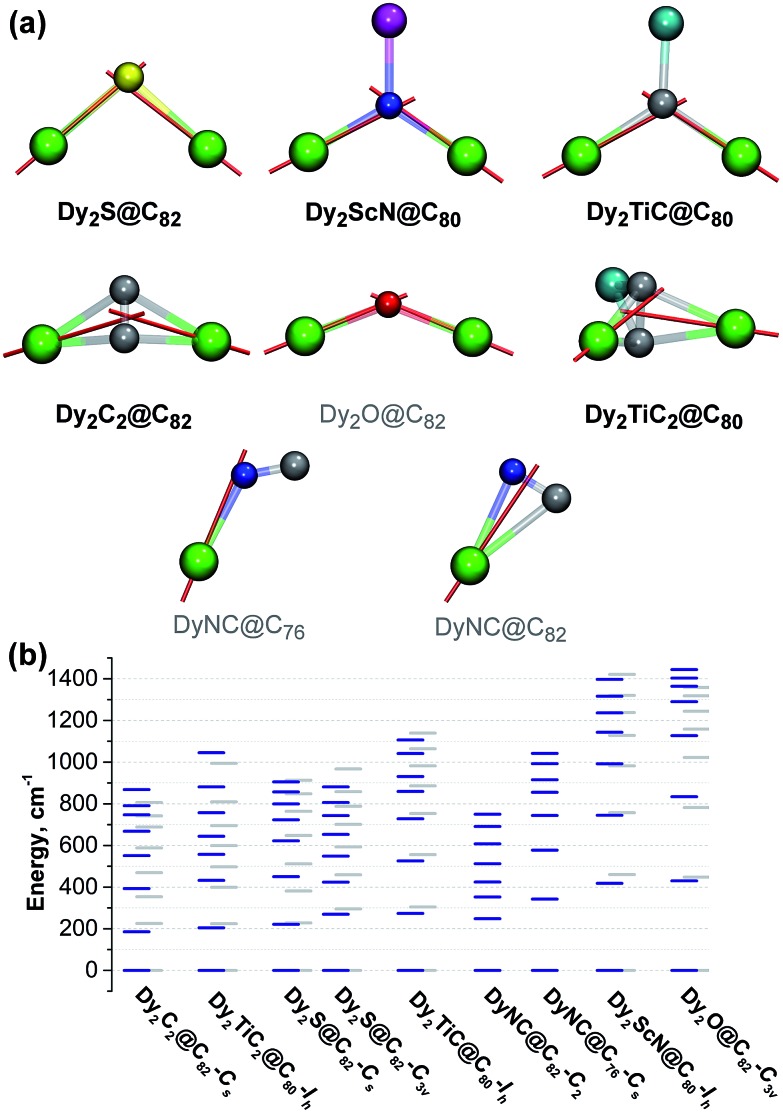
(a) Endohedral clusters in selected clusterfullerenes and magnetic anisotropy axes (shown as red lines) for each Dy center according to *ab initio* calculations. Color code: Dy – green, Sc – magenta, Ti – cyan, S – yellow, C – gray, N – blue, and O – red; carbon cages are omitted for clarity. The compounds not studied experimentally are marked in gray. (b) Computed energies of CF states in different clusterfullerenes (when the molecule has two Dy ions, the energies for each center are given in blue and gray).

In clusterfullerenes with a single non-metal atom, the magnetic anisotropy axis is aligned along the bond connecting Dy to the central atom, sometimes with a slight deviation of a few grad (Fig. 9). In carbide clusterfullerenes with an acetylide unit, the anisotropy axis is directed towards the point between the two carbon atoms, whereas in clusterfullerenes with CN^–^ ions the axis is directed towards more negatively charged nitrogen, but significantly deviates from the metal-nitrogen axis.

Among the EMFs with experimentally studied magnetic properties, Dy_2_ScN@C_80_-*I*_h_ has the largest Δ*E*_1–2_ and Δ*E*_1–8_ values (418/460 and 1397/1421 cm^–1^, respectively; similar values were predicted for this molecule by Chibotaru *et al.*^32^). The Dy–N distances in Dy_2_ScN@C_80_, 2.107/2.111 Å, are much shorter than the Dy–S distances in clusterfullerenes, whereas the formal charge of the nitride ion is higher, which altogether explains the substantially larger CF splitting. Dy_2_TiC@C_80_ is very similar to Dy_2_ScN@C_80_ in its charge distribution and has slightly longer bonds between Dy and the central carbon (2.176/2.192 Å) than in the nitride clusterfullerene. Nonetheless, it has considerably smaller Δ*E*_1–2_ and Δ*E*_1–8_ splitting (273/304 and 1106/1139 cm^–1^, respectively) than in Dy_2_ScN@C_80_-*I*_h_, but still slightly higher than in the sulfide clusterfullerenes. In Dy_2_TiC_2_@C_80_-*I*_h_, the CF splitting is much smaller (204/224 and 1045/994 cm^–1^ for Δ*E*_1–2_ and Δ*E*_1–8_, respectively), which makes it similar to Dy_2_C_2_@C_82_-*C*_s_.

Interestingly, although none of the relaxation processes described in the EMF-SMMs so far involve the first CF excited state, there is an empirical correlation between the strength of the EMF-SMM and the Δ*E*_1–2_ gap. Dy_2_ScN@C_80_-*I*_h_ is the best EMF-SMM so far followed by Dy_2_TiC@C_80_-*I*_h_, which is comparable to Dy_2_S@C_82_-*C*_3v_. Dy_2_S@C_82_-*C*_s_ has a smaller Δ*E*_1–2_ energy than the *C*_3v_ isomer and exhibits poorer SMM properties, and Dy_2_C_2_@C_82_-*C*_s_ is inferior to Dy_2_S@C_82_-*C*_s_. If this correlation holds for other EMFs, then the oxide clusterfullerene Dy_2_O@C_82_-*C*_3v_ may become a superior SMM than Dy_2_ScN@C_80_ as it has the largest Δ*E*_1–2_ and Δ*E*_1–8_ values (430/448 and 1358/1444 cm^–1^, respectively) in the whole group of computed EMFs. The reasons are the short Dy–O distances (2.041/2.029 Å) and rather large Dy–O–Dy angle of 134°. Even larger CF splitting was predicted recently in mixed-metal Dy–Sc and Dy–Lu oxide clusterfullerenes by Rajaraman *et al.*^84^ Thus, Dy-oxide clusterfullerenes seem to be a reasonable target for the SMM-EMF synthesis. Dy-cyano clusterfullerenes are expected to be comparable to sulfide clusterfullerenes in terms of the CF splitting. Flexible cluster geometry from almost linear in MNC@C_76_ to triangular in MNC@C_82_ leads to a large variation of the CF splitting (Fig. 9).

To evaluate the effect of dynamical correlation on CF splitting, a series of additional calculations were performed for simpler model systems, in which all cage carbon atoms were replaced by point charges corresponding to their formal charges in the respective EMFs. When 18 sextets and 15 quartets are used in the CASSCF computations, the model gives a reasonable agreement with full-molecule calculations at only a fraction of the computational cost. Subsequent multi-reference configuration interaction (MRCI) calculations were then performed with 18 sextets and 8 quartets using the Molpro code.^85^ MRCI calculations show that dynamic correlation increases the Δ*E*_1–2_ energy by *ca.* 10–15% (see ESI†). We can tentatively suggest that due to the lack of dynamical correlation, the CASSCF calculations for Dy-EMFs described above underestimate the CF splitting in a similar manner.

The strength of the molecular magnet is determined not only by the CF splitting, but also by the transition probabilities between the states with opposite spin, which are determined by transverse components (*g*_*xx*_ and *g*_*yy*_) of the *g*-tensor. Our calculations show that the nature of the central atom(s) and the cluster geometry strongly affect the transverse components of the *g*-tensor (see Tables S6–S15† for transition probabilities between single-ion states in all computed Dy-clusterfullerenes). The clusters with compact single non-metal atoms, such as oxide and nitride clusterfullerenes, have the smallest transverse components for several lowest excited states, which leads to the low transition probabilities. Our recent experimental study of the relaxation mechanism in Dy_2_ScN@C_80_-*I*_h_ revealed that the Orbach relaxation process observed at high temperatures corresponds to the relaxation *via* the fifth Kramers doublet.^39^ On the other hand, the clusters with diatomic central units have a considerably higher transverse component of the *g*-tensor, which substantially increases transition probabilities for lower-energy KDs. For C_2_ and CN central units, larger transverse components are observed already for the ground state (which may be another reason for the poorer SMM properties of carbide clusterfullerenes). Sulfide clusterfullerenes with relatively large sulfide ions are inferior to oxide and nitride clusterfullerenes, but are better than carbide and cyano-clusterfullerenes.

### Exchange and dipolar interactions in Dy-EMFs

The low-temperature relaxation dynamics of all three EMF-SMMs with two Dy ions are determined by the exchange/dipolar barrier *U*eff1. That is, due to the dipolar and exchange interactions between the Dy ions, the ground and the first excited state of the dinuclear system are the states in which Dy ions in their single-ion ground state (*J*_z_ = ±15/2) are coupled ferromagnetically and antiferromagnetically, respectively, and the Orbach process proceeds *via* the antiferromagnetic state. Knowing the *U*eff1 values, the *j*_12_ coupling constants in eqn (2) can be computed by matching the lowest excited state energy (Table 3).

**Table 3:** Exchange and dipolar coupling parameters in di-Dy EMFs

	*U* eff 1 , K	*j* exp 12 , cm^–1^	α, °	Δ*E*^dip^, K	*j* dip 12 , cm^–1^	*j* ex 12 ,^*a*^ cm^–1^
Dy_2_S@C_72_			78.8	3.4	0.051	0.013
Dy_2_S@C_82_-*C*_s_	15.2	0.220	77.6	3.6	0.051	0.136
Dy_2_S@C_82_-*C*_3v_	6.5	0.104	78.9	3.7	0.059	0.063
Dy_2_C_2_@C_82_-*C*_s_	17.4	0.175	71.8	3.7	0.025	0.108
Dy_2_ScN@C_80_	10.5	0.073	62.8	4.6	0.031	0.128
Dy_2_TiC@C_80_			62.3	4.4	0.029	0.159
Dy_2_TiC_2_@C_80_			71.8	5.0	0.050	0.073
Dy_2_O@C_82_-*C*_3v_			45.7	4.4	0.018	0.110

^*a*^To compute exchange coupling constants, *j*ex12(Gd–Gd) values in Gd-EMF analogs were first computed using Orca code^86^ at the PBE0/TZVP-DKH^87^,^88^ level within the broken-symmetry approximation, and then scaled by a factor of 25/49 cos(*α*).

Dipolar contributions to *U*eff1 energies (Table 3) are calculated straightforwardly using the equation:4
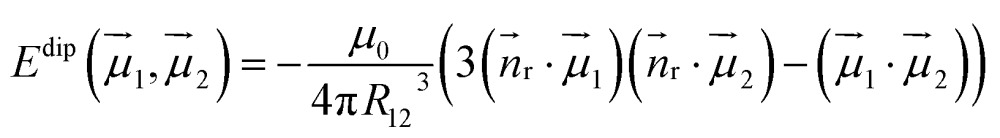
where *n*_r_ is the normal of the radius vector connecting two magnetic moments *µ*_1_ and *µ*_2_, and *R*_12_ is the distance between them. The angles between the moments are taken from *ab initio* calculations. The Δ*E*^dip^ values listed in Table 3 show that for all EMFs the dipolar contribution is in the range of 3.4–5 K. Δ*E*^dip^ constitutes roughly a half of the *U*eff1 barriers in Dy_2_S@C_82_-*C*_3v_ and Dy_2_ScN@C_80_, but is below 25% of that in Dy_2_S@C_82_-*C*_s_ and Dy_2_C_2_@C_82_-*C*_s_. From Δ*E*^dip^, the dipolar contribution *j*dip12 to the *j*_12_ constant in eqn (3) is computed by scaling with the factor of 15^2^ cos(*α*), where *α* is the angle between the anisotropy axes of individual Dy ions.

The calculation of the exchange contribution to the coupling constant for Dy is not straightforward, and it is a common praxis to use Gd analogs to estimate *j*ex12. For the latter, exchange coupling constants are computed using broken-symmetry approximation at the DFT level, and the different spin moments of Dy and Gd as well as non-collinearity of magnetic moments is accounted for by multiplying with the factor of 25/49 cos(*α*).^32^ The values calculated this way for Dy-EMFs (Table 3) are in reasonable agreement with experiment. Similar *j*ex12 values are predicted for all other Dy-EMFs, showing that the extent of the exchange/dipolar coupling between the magnetic moment of Dy ions in clusterfullerenes is not dramatically changing with variation of the central atom(s). Remarkably, the *j*ex12 value in Dy_2_S@C_82_-*C*_3v_ is predicted to be considerably smaller than that in the *C*_s_ isomer.

Once the CF and coupling parameter in the spin Hamiltonian are known or estimated, the solution of eqn (3) allows simulation of the magnetization curves. Low-temperature experimental magnetization curves (Fig. 6 and S23†) have peculiarities at 1.5–2 T, whose presence is caused by exchange/dipolar interactions and hence can be used to verify the computational model. For Dy_2_C_2_@C_82_-*C*_s_ and Dy_2_S@C_82_-*C*_s_, the use of *j*_12_ parameters fitted to match the experimental *U*eff1 values (0.175 and 0.220 cm^–1^, respectively) leads to good agreement between simulated and experimental curves, confirming the assignment of *U*eff1 to the exchange/dipolar barrier. However, for Dy_2_S@C_82_-*C*_3v_, the agreement with experiment is less satisfactory (Fig. S23†). To match the experimental magnetization curve, the *j*_12_ parameter should be increased from 0.104 cm^–1^ to 0.18 cm^–1^, which amounts to the calculated *U*eff1 barrier of 11 K. The discrepancy between experimental and calculated *U*eff1 is likely to be caused by the not-well defined geometry of the Dy_2_S cluster as exchange parameters are very sensitive to the Dy–S–Dy angle, and for the *C*_3v_ isomer the cluster is not fixed in one positon but is rather disordered between several ones.

### Orbach relaxation *via* CF states

The large *U*eff3 barriers of hundreds of K observed in both isomers of Dy_2_S@C_82_ are indicative of the relaxation *via* CF states. It is usually assumed that in polynuclear systems, the Orbach mechanism involves CF states of the individual lanthanide ion. In Dy_2_S@C_82_-*C*_3v_, *ab initio* CF splitting and transition probability calculations show that the barriers for the relaxation *via* individual CF states of single Dy ions unperturbed by interaction with another Dy center may be expected in the range of up to 1000 K (corresponding to the fifth Kramer doublet), whereas the total CF splitting is exceeding 1300 K (Fig. 9). Within the limits of rather high experimental uncertainties, the experimental value is in line with this expectation. For Dy_2_S@C_82_-*C*_s_, *ab initio* calculations show that relaxation *via* lower-energy Kramers doublets can also be efficient (see Table S6†). This expectation is also in line with the lower *U*eff3 value observed for this compound experimentally (Table 1), but currently impossible higher-frequency AC measurements would be necessary to confirm the *U*eff3 barrier in Dy_2_S@C_82_-*C*_s_.

### Intermediate barrier

The nature of the Orbach relaxation processes in Dy_2_S@C_82_ isomers with barriers of 50–60 K cannot be explained based on the energy spectrum of the Hamiltonian (3). The *U*eff2 values are clearly above the exchange/dipolar barrier and yet well below the energies of the CF-derived excited states. Besides, *τ*_02_ values are also much longer than would be expected for the relaxation through CF excited states.

Multiple studies of the electron spin-lattice relaxation times in salts of transition metals and lanthanides starting from the early 1960s and later on revealed that relaxation *via* the Raman mechanism in the presence of the so-called localized phonon of frequency *ω* (usually associated with defects in those studies) can take an exponential form proportional to exp(–*ħω*/*k*_B_*T*).^89^–^91^ In other words, it can be described as an Orbach relaxation process with the energy barrier corresponding to the phonon excited state. Orbach relaxation processes with barriers corresponding to the frequencies of molecular vibrations were also observed in N@C_60_ (ref. 92) and other paramagnetic solids and host–guest systems.^93^–^95^ Very recently, Sanvito *et al.* studied the role of phonons in the under-barrier spin relaxation of SMMs and found that an anharmonic phonon with finite linewidth may result in the Arrhenius behavior with the barrier corresponding to a half of the phonon frequency.^96^

**Fig. 10:**
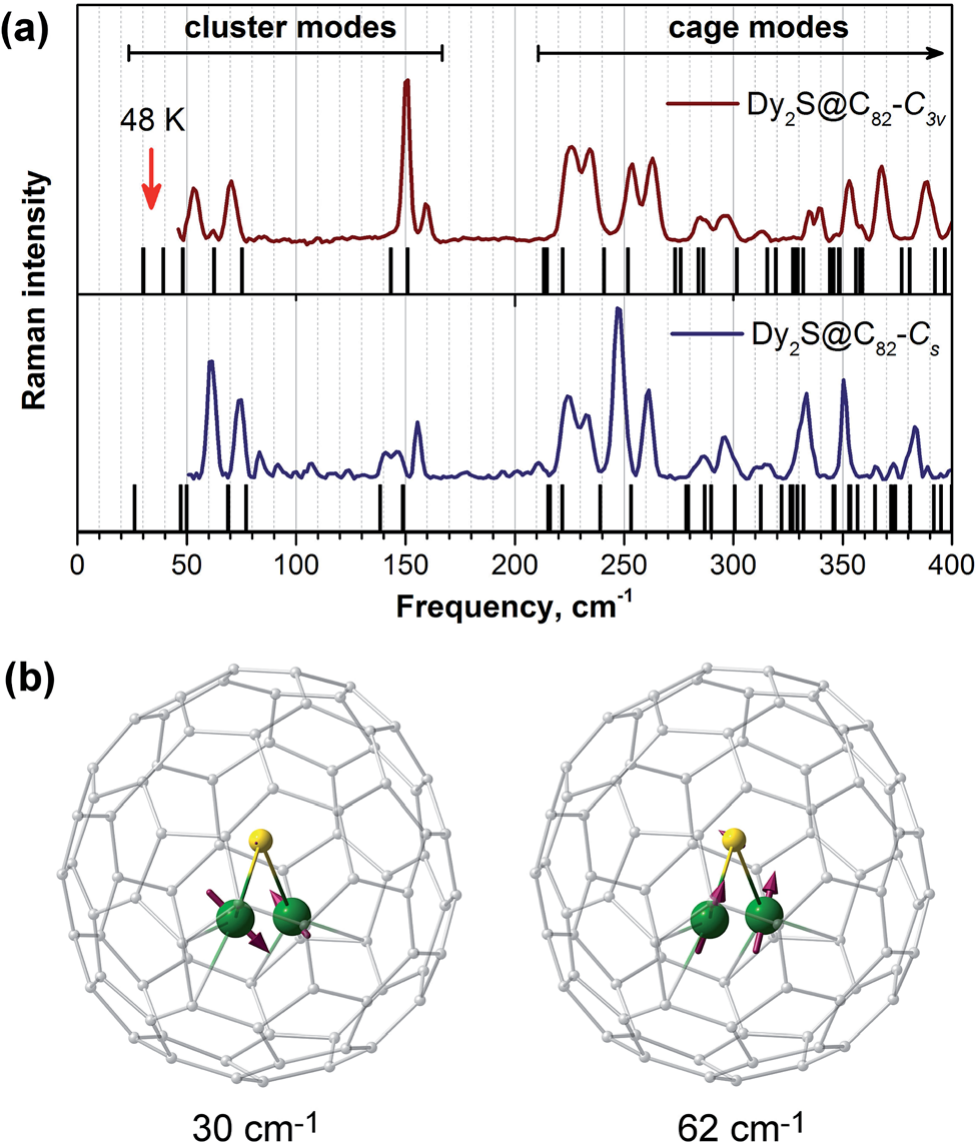
(a) Low-energy part of the Raman spectra of Dy_2_S@C_82_-*C*_3v_ (top) and Dy_2_S@C_82_-*C*_s_ (bottom) compared to DFT-computed vibrational frequencies of individual molecules (black lines). (b) Atomic displacements of the two vibrations of the Dy_2_S cluster in Dy_2_S@C_82_-*C*_3v_.

To our knowledge, the possibility of Orbach relaxation *via* an excited phonon state has not been widely considered for SMMs. Usually, SMMs have rather high vibrational density of states in the low frequency range due to the presence of “floppy” fragments and side chains in the ligands. However, fullerene molecules are quite rigid, and their lowest frequency vibrations occur above 200 cm^–1^. In EMFs, encapsulated clusters with heavy lanthanide atoms have few low-frequency vibrational modes due to frustrated rotations and translation as well as internal cluster vibrations. In the Raman spectra of Dy_2_S@C_82_ isomers shown in Fig. 10a, the cage (above 220 cm^–1^) and the cluster (between 50 and 160 cm^–1^) vibrational features are well separated. The frequencies of ∼40 cm^–1^ corresponding to *U*eff2 values lie outside the accessible range of our spectrometer, but DFT computations show the presence of cluster vibrations in this frequency range, mainly of the librational character (in Dy_2_S@C_82_-*C*_3v_ such modes are predicted at 30, 39, and 48 cm^–1^). It is reasonable to suggest that the lowest-frequency librational mode may be responsible for the Orbach relaxation process with a barrier of 48 K (33 cm^–1^). On the other hand, if following Sanvito *et al.* we suggest that the observed barrier corresponds to a half of the phonon frequency,^96^ then the relaxation of magnetization in Dy_2_S@C_82_ may be induced by the mixed translation/deformation mode of the Dy_2_S cluster with the calculated frequency of 62 cm^–1^ (Fig. 10b).

Thus, shielding of endohedral species by the carbon cage not only stabilizes the otherwise “improper” endohedral species (none of the clusters discussed in this work can exist outside the fullerene), but also isolates the Dy spin system from the lattice phonon bath, resulting in a kind of phonon bottlenecking at low temperatures. When the local vibrational modes gain certain thermal population, a new Orbach relaxation pathway is open and the rate of relaxation is accelerated. The sparse vibrational density of states in EMFs may be the reason for the long relaxation times that these molecules exhibit at low temperatures. Further studies of the low-frequency vibrational density of states as well as development of a rigorous theory of the spin-phonon relaxation in SMMs are required to confirm this hypothesis.

## Conclusions

In this article, we report a new method for the selective synthesis of sulfide clusterfullerenes. We utilized the suppressing influence of hydrogen on the empty fullerene formation and performed arc-discharge synthesis in the presence of methane. Under optimized conditions, and with the use of dysprosium sulfide as a source of metal and sulfur, Dy_2_S@C_2*n*_ clusterfullerenes could be synthesized with a high degree of selectivity, and molecular structures of the most abundant Dy-sulfide clusterfullerenes, Dy_2_S@C_82_ isomers with *C*_s_(6) and *C*_3v_(8) cage symmetry, were elucidated by single-crystal X-ray diffraction. The yield of carbide clusterfullerenes appears to be much lower when sulfur is present in the system. This finding shows that the clusterfullerenes with other central atoms may also be selectively synthesized this way with the proper choice of arc-discharge conditions.

The magnetic properties of Dy-sulfide clusterfullerenes, Dy_2_S@C_82_-*C*_s_ and Dy_2_S@C_82_-*C*_3v_, and of one Dy-carbide clusterfullerene, Dy_2_C_2_@C_82_-*C*_s_, have been further studied by DC and AC magnetometry and *ab initio* calculations. All molecules were found to be single molecule magnets with hysteresis of magnetization below 3–4 K, and with substantially different cage- and cluster-dependent relaxation rates. Among the two isomers of Dy_2_S@C_82_, the one with the *C*_3v_(8) carbon cage is a far more superior SMM than the analogous molecule with the *C*_s_(6) carbon cage, whereas among the two EMFs with the C_82_-*C*_s_(6) fullerene cage, the sulfide clusterfullerene Dy_2_S@C_82_ has longer relaxation times than the carbide clusterfullerene Dy_2_C_2_@C_82_. *Ab initio* calculations for different types of clusterfullerenes showed that the clusters with a single non-metal ion are more preferable for the better SMM performance than the clusterfullerenes with diatomic non-metal units, and oxide clusterfullerenes were found to have the highest crystal field splitting.

Dynamic magnetic studies showed that the relaxation of magnetization in Dy_2_S@C_82_ isomers unprecedentedly involves three Orbach processes operative at different temperatures. Below 5–10 K, the dominant process is the relaxation *via* the exchange/dipolar excited state with antiferromagnetic coupling of Dy ions. At temperatures above 40–50 K, Orbach relaxation *via* crystal-field excited states with relative energies exceeding 500 K is observed. The CF barriers in sulfide clusterfullerenes are among the highest magnetization relaxation barriers observed in dinuclear Dy-SMMs so far. For the intermediate temperatures, we have discovered an unusual Orbach process, whose energy barrier of 50–60 K corresponds to the intramolecular vibrations of the EMF molecules involving librational motions of the endohedral cluster.

## Supplementary Material

Supplementary information

Crystal structure data

## References

[cit1] Sessoli R., Gatteschi D., Caneschi A., Novak M. A. (1993). Nature.

[cit2] Ishikawa N., Sugita M., Ishikawa T., Koshihara S., Kaizu Y. (2003). J. Am. Chem. Soc..

[cit3] Feltham H. L. C., Brooker S. (2014). Coord. Chem. Rev..

[cit4] Layfield R. A. (2014). Organometallics.

[cit5] Habib F., Murugesu M. (2013). Chem. Soc. Rev..

[cit6] Zhang P., Zhang L., Tang J. (2015). Dalton Trans..

[cit7] Dreiser J. (2015). J. Phys.: Condens. Matter.

[cit8] Woodruff D. N., Winpenny R. E. P., Layfield R. A. (2013). Chem. Rev..

[cit9] Luzon J., Sessoli R. (2012). Dalton Trans..

[cit10] Rinehart J. D., Long J. R. (2011). Chem. Sci..

[cit11] Sorace L., Benelli C., Gatteschi D. (2011). Chem. Soc. Rev..

[cit12] Liddle S. T., van Slageren J. (2015). Chem. Soc. Rev..

[cit13] Ding Y.-S., Chilton N. F., Winpenny R. E. P., Zheng Y.-Z. (2016). Angew. Chem., Int. Ed. Engl..

[cit14] Gupta S. K., Rajeshkumar T., Rajaraman G., Murugavel R. (2016). Chem. Sci..

[cit15] Chilton N. F. (2015). Inorg. Chem..

[cit16] Rinehart J. D., Fang M., Evans W. J., Long J. R. (2011). Nat. Chem..

[cit17] Huang W., Shen F.-X., Wu S.-Q., Liu L., Wu D., Zheng Z., Xu J., Zhang M., Huang X.-C., Jiang J., Pan F., Li Y., Zhu K., Sato O. (2016). Inorg. Chem..

[cit18] Xiong J., Ding H.-Y., Meng Y.-S., Gao C., Zhang X.-J., Meng Z.-S., Zhang Y.-Q., Shi W., Wang B.-W., Gao S. (2017). Chem. Sci..

[cit19] Guo Y.-N., Xu G.-F., Wernsdorfer W., Ungur L., Guo Y., Tang J., Zhang H.-J., Chibotaru L. F., Powell A. K. (2011). J. Am. Chem. Soc..

[cit20] Zou H.-H., Sheng L.-B., Liang F.-P., Chen Z.-L., Zhang Y.-Q. (2015). Dalton Trans..

[cit21] Langley S. K., Wielechowski D. P., Vieru V., Chilton N. F., Moubaraki B., Chibotaru L. F., Murray K. S. (2014). Chem. Sci..

[cit22] Andruh M., Costes J. P., Diaz C., Gao S. (2009). Inorg. Chem..

[cit23] Li X.-L., Min F.-Y., Wang C., Lin S.-Y., Liu Z., Tang J. (2015). Inorg. Chem..

[cit24] Rosado Piquer L., Sanudo E. C. (2015). Dalton Trans..

[cit25] Liu K., Shi W., Cheng P. (2015). Coord. Chem. Rev..

[cit26] Popov A. A., Yang S., Dunsch L. (2013). Chem. Rev..

[cit27] Lu X., Feng L., Akasaka T., Nagase S. (2012). Chem. Soc. Rev..

[cit28] Yang S., Liu F., Chen C., Jiao M., Wei T. (2011). Chem. Commun..

[cit29] Wolf M., Muller K. H., Skourski Y., Eckert D., Georgi P., Krause M., Dunsch L. (2005). Angew. Chem., Int. Ed..

[cit30] Zhang Y., Krylov D., Rosenkranz M., Schiemenz S., Popov A. A. (2015). Chem. Sci..

[cit31] Zhang Y., Krylov D., Schiemenz S., Rosenkranz M., Westerstrom R., Dreiser J., Greber T., Buchner B., Popov A. A. (2014). Nanoscale.

[cit32] Vieru V., Ungur L., Chibotaru L. F. (2013). J. Phys. Chem. Lett..

[cit33] Cimpoesu F., Dragoe N., Ramanantoanina H., Urland W., Daul C. (2014). Phys. Chem. Chem. Phys..

[cit34] Svitova A. L., Krupskaya Y., Samoylova N., Kraus R., Geck J., Dunsch L., Popov A. A. (2014). Dalton Trans..

[cit35] Hermanns C. F., Bernien M., Krüger A., Schmidt C., Waßerroth S. T., Ahmadi G., Heinrich B. W., Schneider M., Brouwer P. W., Franke K. J., Weschke E., Kuch W. (2013). Phys. Rev. Lett..

[cit36] Náfrádi B., Antal Á., Pásztor Á., Forró L., Kiss L. F., Fehér T., Kováts É., Pekker S., Jánossy A. (2012). J. Phys. Chem. Lett..

[cit37] Westerström R., Dreiser J., Piamonteze C., Muntwiler M., Weyeneth S., Brune H., Rusponi S., Nolting F., Popov A., Yang S., Dunsch L., Greber T. (2012). J. Am. Chem. Soc..

[cit38] Westerström R., Dreiser J., Piamonteze C., Muntwiler M., Weyeneth S., Krämer K., Liu S.-X., Decurtins S., Popov A., Yang S., Dunsch L., Greber T. (2014). Phys. Rev. B: Condens. Matter Mater. Phys..

[cit39] Krylov D. S., Liu F., Avdoshenko S. M., Spree L., Weise B., Waske A., Wolter A. U. B., Büchner B., Popov A. A. (2017). Chem. Commun..

[cit40] Westerström R., Uldry A.-C., Stania R., Dreiser J., Piamonteze C., Muntwiler M., Matsui F., Rusponi S., Brune H., Yang S., Popov A., Büchner B., Delley B., Greber T. (2015). Phys. Rev. Lett..

[cit41] Dreiser J., Westerström R., Zhang Y., Popov A. A., Dunsch L., Krämer K., Liu S.-X., Decurtins S., Greber T. (2014). Chem.–Eur. J..

[cit42] Junghans K., Schlesier C., Kostanyan A., Samoylova N. A., Deng Q., Rosenkranz M., Schiemenz S., Westerström R., Greber T., Büchner B., Popov A. A. (2015). Angew. Chem., Int. Ed. Engl..

[cit43] Liu F., Gao C.-L., Deng Q., Zhu X., Kostanyan A., Westerström R., Wang S., Tan Y.-Z., Tao J., Xie S.-Y., Popov A. A., Greber T., Yang S. (2016). J. Am. Chem. Soc..

[cit44] Liu F., Wang S., Gao C.-L., Deng Q., Zhu X., Kostanyan A., Westerström R., Jin F., Xie S.-Y., Popov A. A., Greber T., Yang S. (2017). Angew. Chem., Int. Ed. Engl..

[cit45] Dunsch L., Yang S., Zhang L., Svitova A., Oswald S., Popov A. A. (2010). J. Am. Chem. Soc..

[cit46] Chen N., Chaur M. N., Moore C., Pinzon J. R., Valencia R., Rodriguez-Fortea A., Poblet J. M., Echegoyen L. (2010). Chem. Commun..

[cit47] Dunsch L., Krause M., Noack J., Georgi P. (2004). J. Phys. Chem. Solids.

[cit48] Dunsch L., Georgi P., Krause M., Wang C. R. (2003). Synth. Met..

[cit49] Yang S., Zhang L., Zhang W., Dunsch L. (2010). Chem.–Eur. J..

[cit50] Liu F., Guan J., Wei T., Wang S., Jiao M., Yang S. (2013). Inorg. Chem..

[cit51] Svitova A. L., Popov A. A., Dunsch L. (2013). Inorg. Chem..

[cit52] Jiao M., Zhang W., Xu Y., Wei T., Chen C., Liu F., Yang S. (2012). Chem.–Eur. J..

[cit53] Stevenson S., Thompson M. C., Coumbe H. L., Mackey M. A., Coumbe C. E., Phillips J. P. (2007). J. Am. Chem. Soc..

[cit54] Junghans K., Rosenkranz M., Popov A. A. (2016). Chem. Commun..

[cit55] Junghans K., Ghiassi K. B., Samoylova N. A., Deng Q., Rosenkranz M., Olmstead M. M., Balch A. L., Popov A. A. (2016). Chem.–Eur. J..

[cit56] Deng Q., Junghans K., Popov A. A. (2015). Theor. Chem. Acc..

[cit57] Svitova A. L., Ghiassi K., Schlesier C., Junghans K., Zhang Y., Olmstead M., Balch A., Dunsch L., Popov A. A. (2014). Nat. Commun..

[cit58] Samoylova N. A., Avdoshenko S. M., Krylov D. S., Thompson H. R., Kirkhorn A., Rosenkranz M., Schiemenz S., Ziegs F., Wolter A. U. B., Yang S., Stevenson S., Popov A. A. (2017). Nanoscale.

[cit59] Lu X., Nakajima K., Iiduka Y., Nikawa H., Mizorogi N., Slanina Z., Tsuchiya T., Nagase S., Akasaka T. (2011). J. Am. Chem. Soc..

[cit60] Inoue T., Tomiyama T., Sugai T., Okazaki T., Suematsu T., Fujii N., Utsumi H., Nojima K., Shinohara H. (2004). J. Phys. Chem. B.

[cit61] Iiduka Y., Wakahara T., Nakajima K., Tsuchiya T., Nakahodo T., Maeda Y., Akasaka T., Mizorogi N., Nagase S. (2006). Chem. Commun..

[cit62] Chen N., Beavers C. M., Mulet-Gas M., Rodriguez-Fortea A., Munoz E. J., Li Y.-Y., Olmstead M. M., Balch A. L., Poblet J. M., Echegoyen L. (2012). J. Am. Chem. Soc..

[cit63] Olmstead M. M., Costa D. A., Maitra K., Noll B. C., Phillips S. L., Van Calcar P. M., Balch A. L. (1999). J. Am. Chem. Soc..

[cit64] Mueller U., Förster R., Hellmig M., Huschmann F. U., Kastner A., Malecki P., Pühringer S., Röwer M., Sparta K., Steffien M., Ühlein M., Wilk P., Weiss M. S. (2015). Eur. Phys. J. Plus.

[cit65] Sparta K. M., Krug M., Heinemann U., Mueller U., Weiss M. S. (2016). J. Appl. Crystallogr..

[cit66] Sheldrick G. (2015). Acta Crystallogr., Sect. C: Struct. Chem..

[cit67] Mercado B. Q., Chen N., Rodriguez-Fortea A., Mackey M. A., Stevenson S., Echegoyen L., Poblet J. M., Olmstead M. M., Balch A. L. (2011). J. Am. Chem. Soc..

[cit68] Mondal K. C., Sundt A., Lan Y., Kostakis G. E., Waldmann O., Ungur L., Chibotaru L. F., Anson C. E., Powell A. K. (2012). Angew. Chem., Int. Ed. Engl..

[cit69] Li X.-L., Min F.-Y., Wang C., Lin S.-Y., Liu Z., Tang J. (2015). Dalton Trans..

[cit70] Langley S. K., Wielechowski D. P., Vieru V., Chilton N. F., Moubaraki B., Abrahams B. F., Chibotaru L. F., Murray K. S. (2013). Angew. Chem., Int. Ed. Engl..

[cit71] Li J., Wei R.-M., Pu T.-C., Cao F., Yang L., Han Y., Zhang Y.-Q., Zuo J.-L., Song Y. (2017). Inorg. Chem. Front..

[cit72] Blagg R. J., Ungur L., Tuna F., Speak J., Comar P., Collison D., Wernsdorfer W., McInnes E. J. L., Chibotaru L. F., Winpenny R. E. P. (2013). Nat. Chem..

[cit73] Tuna F., Smith C. A., Bodensteiner M., Ungur L., Chibotaru L. F., McInnes E. J. L., Winpenny R. E. P., Collison D., Layfield R. A. (2012). Angew. Chem., Int. Ed. Engl..

[cit74] Liu S.-S., Lang K., Zhang Y.-Q., Yang Q., Wang B.-W., Gao S. (2016). Dalton Trans..

[cit75] Cao W., Gao C., Zhang Y.-Q., Qi D., Liu T., Wang K., Duan C., Gao S., Jiang J. (2015). Chem. Sci..

[cit76] Aquilante F., Autschbach J., Carlson R. K., Chibotaru L. F., Delcey M. G., De Vico L., Galván I. F., Ferré N., Frutos L. M., Gagliardi L., Garavelli M., Giussani A., Hoyer C. E., Li Manni G., Lischka H., Ma D., Malmqvist P. Å., Müller T., Nenov A., Olivucci M., Pedersen T. B., Peng D., Plasser F., Pritchard B., Reiher M., Rivalta I., Schapiro I., Segarra-Martí J., Stenrup M., Truhlar D. G., Ungur L., Valentini A., Vancoillie S., Veryazov V., Vysotskiy V. P., Weingart O., Zapata F., Lindh R. (2016). J. Comput. Chem..

[cit77] Chibotaru L. F., Ungur L. (2012). J. Chem. Phys..

[cit78] Chilton N. F., Anderson R. P., Turner L. D., Soncini A., Murray K. S. (2013). J. Comput. Chem..

[cit79] Liu F., Wang S., Guan J., Wei T., Zeng M., Yang S. (2014). Inorg. Chem..

[cit80] Zhang M., Hao Y., Li X., Feng L., Yang T., Wan Y., Chen N., Slanina Z., Uhlik F., Cong H. (2014). J. Phys. Chem. C.

[cit81] Mercado B. Q., Stuart M. A., Mackey M. A., Pickens J. E., Confait B. S., Stevenson S., Easterling M. L., Valencia R., Rodriguez-Fortea A., Poblet J. M., Olmstead M. M., Balch A. L. (2010). J. Am. Chem. Soc..

[cit82] Tang Q., Abella L., Hao Y., Li X., Wan Y., Rodríguez-Fortea A., Poblet J. M., Feng L., Chen N. (2016). Inorg. Chem..

[cit83] Stevenson S., in Endohedral Fullerenes. From Fundamentals to Applications, ed. S. Yang and C.-R. Wang, World Scientific, Singapore, 2014, pp. 179–210.

[cit84] Singh M. K., Rajaraman G. (2016). Chem. Commun..

[cit85] Werner H.-J., Knowles P. J., Knizia G., Manby F. R., Schütz M. (2012). Wiley Interdiscip. Rev.: Comput. Mol. Sci..

[cit86] Neese F. (2012). Wiley Interdiscip. Rev.: Comput. Mol. Sci..

[cit87] Pantazis D. A., Neese F. (2009). J. Chem. Theory Comput..

[cit88] Pantazis D. A., Chen X.-Y., Landis C. R., Neese F. (2008). J. Chem. Theory Comput..

[cit89] Klemens P. G. (1962). Phys. Rev..

[cit90] Mills D. L. (1966). Phys. Rev..

[cit91] Walker M. B. (1967). Phys. Rev..

[cit92] Morton J. J. L., Tyryshkin A. M., Ardavan A., Porfyrakis K., Lyon S. A., Briggs G. A. D. (2006). J. Chem. Phys..

[cit93] Goslar J., Hoffmann S. K., Hilczer W. (2002). Solid State Commun..

[cit94] Hoffmann S. K., Goslar J. (2015). J. Phys.: Condens. Matter.

[cit95] Goslar J., Lijewski S., Hoffmann S. K., Jankowska A., Kowalak S. (2009). J. Chem. Phys..

[cit96] Lunghi A., Totti F., Sessoli R., Sanvito S. (2017). Nat. Commun..

