# Supplementary material for: Selective arc-discharge synthesis of Dy2S-clusterfullerenes and their isomer-dependent single molecule magnetism
Source: Chem Sci. 2017 Jun 30;8(9):6451–65. doi: 10.1039/c7sc02395b (PMC5734629; doi:10.1039/c7sc02395b)
Supplement: Supplementary file 1 [file SC-008-C7SC02395B-s001.pdf]

Electronic Supplementary Material (ESI) for Chemical Science.
This journal is © The Royal Society of Chemistry 2017

Selective arc-discharge synthesis of Dy_2S-clusterfullerenes and their isomer-dependent single molecule magnetism

Chia-Hsiang Chen,[1$] Denis S. Krylov,[1$] Stanislav M. Avdoshenko,[1] Fupin Liu,[1] Lukas Spree,[1] Ravi Yadav,[1] Antonis Alvertis,[1] Liviu Hozoi,[1] Konstantin Nenkov,[1] Aram Kostanyan,[2] Thomas Greber,[2] Anja Wolter-Giraud,[1] Alexey A. Popov[1]*

Supporting Information

Experimental Section S2

Synthesis and isolation of sulfide and carbide clusterfullerenes S3

Synthesis of Dy-sulfide EMFs by using different sulfur sources S5

Optimization of the conditions of synthesis of $Dy_2S@C_{82}$ S6

HPLC chromatograms of purified EMFs S7

DFT computations of $Y_2S@C_{82}$ isomers S8

Relative energies of C_{72}^{4-} and $Y_2S@C_{72}$ isomers S9

Additional crystallographic data S10

Magnetic properties of Dy-EMFs: χT products S15

AC measurements of $Dy_2S@C_{82}$-C_s S16

AC measurements of $Dy_2S@C_{82}$-C_{3v} S17

AC measurements of $Dy_2C_2@C_{82}$-C_s S18

Relaxation times S19

Magnetization relaxation dynamics in $Dy_2C_2@C_{82}$-C_s S22

Magnetization relaxation dynamics in $Dy_2C_2@C_{82}$-C_{3v} S23

Ab initio calculation with Molcas S24

Ab initio calculations with Molpro S42

Broken-symmetry calculations of di-Gd analogs S46

Energy spectrum of the total spin Hamiltonian S47

Magnetization curves at T = 3K: experiment versus simulation S48

DFT-optimized Cartesian coordinates of the molecules S49

Experimental Section

General methods.

Graphite rods (6 x 10 mm) were used as received. Vis-NIR spectra were recorded from 400 to 2000 nm in carbon disulfide by using a 1.0 cm quartz cell in a Shimadzu 3100 spectrophotometer. ***MALDI mass spectra*** were recorded on a Bruker autoflex mass-spectrometer. ***UV-vis-NIR absorption spectra*** were measured in toluene solution at room temperature with Shimadzu 3100 spectrophotometer. ***Raman spectra*** were recorded at 78 K on a T 64000 triple spectrometer (Jobin Yvon) using a 647 nm excitation wavelength of the Kr laser. For Raman measurements, the samples were drop-casted from toluene solutions onto single-crystal KBr disks.

DC magnetization measurements were performed using a Quantum Design VSM MPMS3 magnetometer. Pure $Dy_2ScN@C_{80}$ samples were drop casted from toluene solution into a standard propylene VSM capsule (note that co-crystallziation with NiOEP was used only for X-ray diffraction studies, whereas magnetic properties were studied for the pristine $Dy_2ScN@C_{80}$). To measure relaxation times, the sample was first magnetized to saturation in the field of 5 Tesla, then the field was sept to 0 T as fast as possible, and then the decay of magnetization was followed. Average relaxation times were then determined from stretched exponential fitting of the decay curves. ***AC measurements*** were performed with three devices: Quantum Design MPMS XL magnetometer for the low-frequency range (< 10 Hz), Quantum Design VSM MPMS3 magnetometer in the middle-frequency range (10-1000 Hz), and PPMS system for the high-frequency range (0.5-10 kHz).

Synthesis and isolation of sulfide and carbide clusterfullerenes

A graphite rod was core-drilled, packed with a mixture of Dy_2S_3 and graphite powder (molar ratio of Dy : S : C = 1 : 1.5 : 10). The graphite rod was then vaporized in a Krätschmer-Huffman-type fullerene generator with an arc current of 100 A under 230 mbar helium and 20 mbr methane. The collected soot was Soxhlet-extracted with carbon disulfide for 12 h. The extract was dried by CS_2 distillation. The solid residue was dissolved in toluene and filtered. The desired compound $Dy_2S@C_{72}$-C_s(10528), $Dy_2S@C_{82}$-C_s(6), $Dy_2S@C_{82}$-C_{3v}(8) and $Dy_2C_2@C_{82}$-C_s(6) were isolated from empty fullerenes and other endohedral fullerenes by two-stage HPLC process. In the first stage, the toluene solution of the extract was separated by using a HPLC monitored using a UV detector at 320 nm and a linear combination of two analytical 4.6 mm × 250 mm Buckyprep columns (Nacalai Tesque, Japan) with toluene as the mobile phase, and fraction **A**, **B**, **C** and **D** were collected (see Figure. S1a). Fraction **A** mainly contains $Dy_2S@C_{72}$-C_s(10528), fraction **B** mainly contains $Dy_3N@C_{80}$ and fraction **D** mainly contains $Dy_2S@C_{82}$-C_{3v}(8). In the second stage, fraction **C** was subjected to recycling HPLC with a 10 × 250 mm Buckyprep column (Nacalai Tesque, Japan), resulting in the isolation of $Dy_2S@C_{82}$-C_s(6) and $Dy_2C_2@C_{82}$-C_s(6) (see Figure S1b).

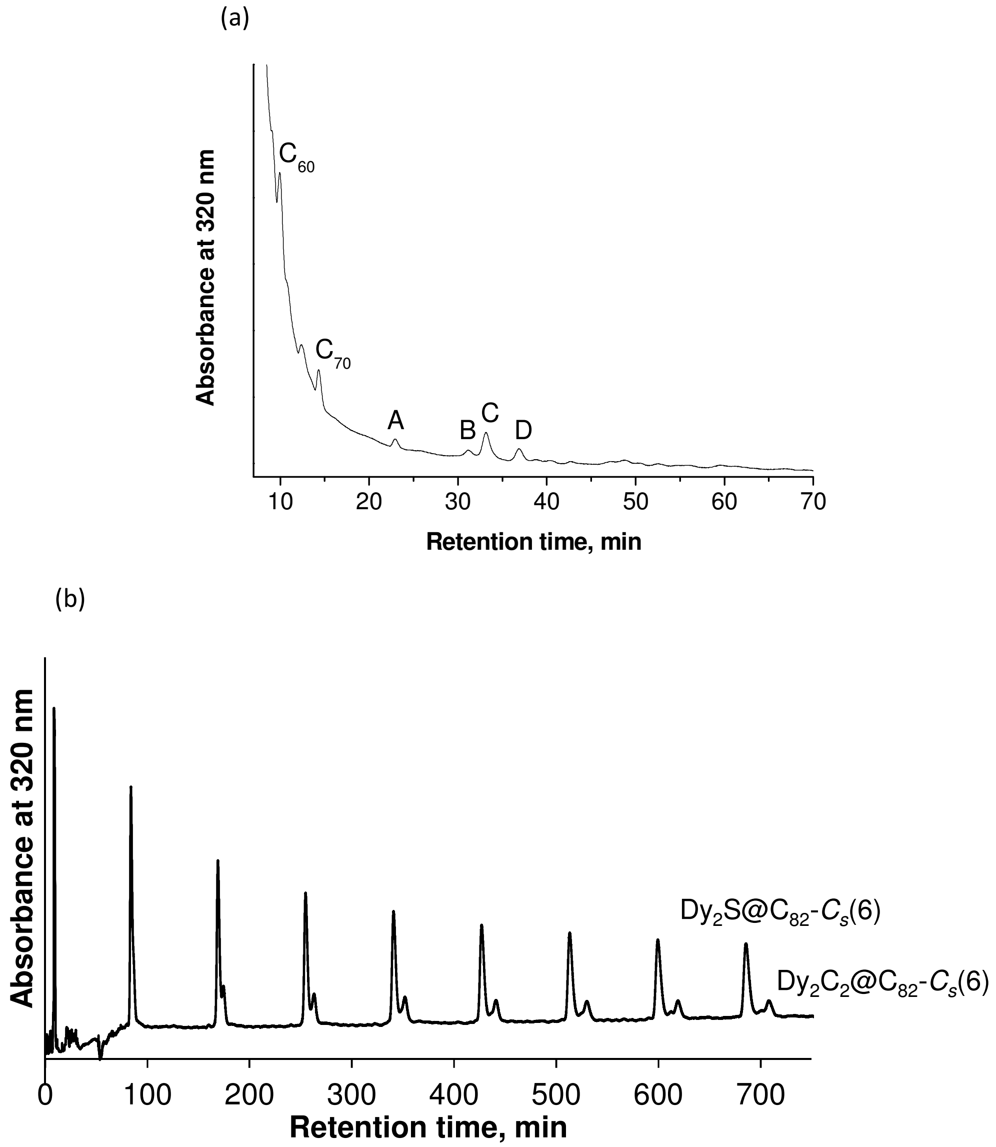

Figure S1. Isolation scheme of Dy sulfide and carbide clusterfullerenes. (a) HPLC chromatogram of the fullerene extract obtained on linear combination of two analytical 4.6 mm × 250 mm Buckyprep columns with λ = 320 nm, a flow rate of 1.6 mL/min, and toluene as the eluent at 40 °C. (b) Recycling HPLC chromatogram of fraction **C** obtained on a 10 mm × 250 mm Buckyprep column with λ= 320 nm, a flow rate of 1 mL/min, and toluene as the eluent at 25 °C.

Synthesis of Dy-sulfide EMFs by using different sulfur sources

To find the better solid sulfur source for the synthesis of the Dy-sulfide EMFs, the graphite rods were core-drilled, packed with a mixture of Dy powder, graphite powder and different solid sulfur sources (molar ratio of Dy : S : C = 1 : 1.5 : 10). We used the different combinations of Dy source and solid sulfur sources (i) mixture of Dy powder with elementary sulfur; (ii) mixture of Dy powder with dibenzyl sulfide; (iii) Dy_2S_3; to study the yield of Dy-sulfide EMFs. The graphite rods were then vaporized in a Krätschmer-Huffman-type fullerene generator with an arc current of 100 A under 237 mbar helium and 13 mbr methane. The collected soot was Soxhlet-extracted with carbon disulfide for 12 h. The extract was dried by CS_2 distillation. The solid residue was dissolved in toluene and filtered. The Dy-sulfide EMFs were isolated from empty fullerenes and other endohedral fullerenes by HPLC. Fig. S2 presents the chromatograms of a series of fullerene extracts obtained from the different combinations of Dy powder and solid sulfur sources, and the fraction **A** mainly contains $Dy_3N@C_{80}$, fraction **B** contains $Dy_2S@C_{82}$-$C_s(6)$ and $Dy_2C_2@C_{82}$-$C_s(6)$, and fraction **C** mainly contains $Dy_2S@C_{82}$-$C_{3v}(8)$. The results indicate the Dy_2S_3 as sulfur source can produce more $Dy_2S@C_{82}$, and suppress the $Dy_3N@C_{80}$ forming.

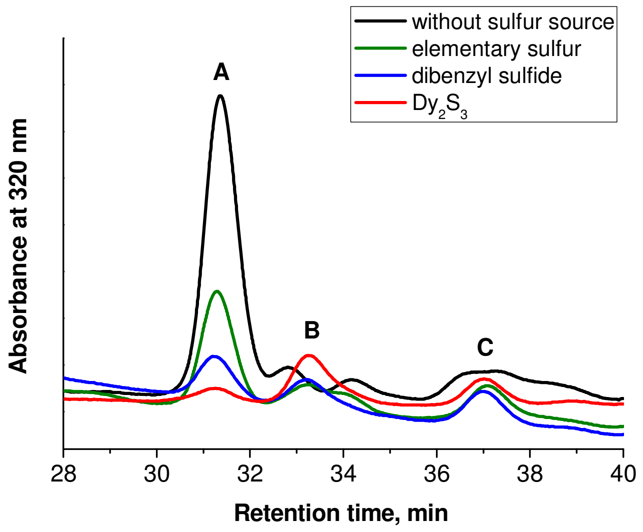

Figure S2. HPLC chromatograms of the extracts synthesized from the different combinations of Dy source and solid sulfur sources (i) Dy powder without sulfur source; (ii) mixture of Dy powder with elementary sulfur; (iii) mixture of Dy powder with dibenzyl sulfide; (iv) Dy_2S_3; (Linear combination of two 4.6 x 250 mm Buckyprep columns; flow rate 1.6 ml/min; toluene as eluent; 40°C).

Optimization of the conditions of synthesis of $Dy_2S@C_{82}$

To optimize the synthesis of Dy-sulfide EMFs by varying the molar ratio of Dy_2S_3 and graphite powder. Fig. S3 presents the chromatograms of a series of fullerene extracts obtained under different molar ratios of Dy:S:C ranging from 0.5:0.75:10 to 3:4.5:10. At relatively low molar ratios of Dy:S:C from 0.5:0.75:10, the yield of the fraction **A** ($Dy_3N@C_{80}$) is higher than the others, but the yield of fraction **B** ($Dy_2S@C_{82}$-C_s(6) and $Dy_2C_2@C_{82}$-C_s(6)) and fraction **C** ($Dy_2S@C_{82}$-C_{3v}(8)) is relatively low. When the molar ratio increases to 1:1.5:10, the relative yield of Dy-sulfide EMFs increases. However, the molar ratio increase to 3:4.5:10, led to decrease the yield of Dy-based EMFs, indicating the higher amount of Dy_2S_3 suppressing the formation of Dy-based EMFs. Fig. S3 shows the effect of the molar ratios of Dy:S:C on the yield of fraction **B** and fraction **C**, which contains the new sulfide clusterfullerenes $Dy_2S@C_{82}$-C_s(6) and $Dy_2S@C_{82}$-C_{3v}(8), and clearly points to the optimum molar ratio of Dy:S:C of 1:1.5:10.

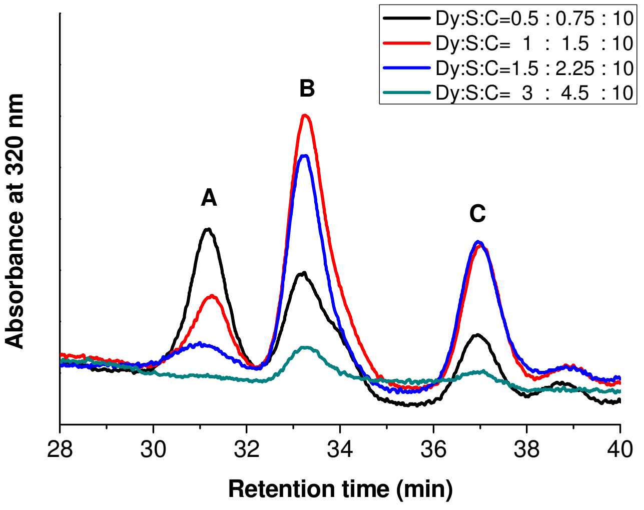

Figure S3. (I) HPLC chromatograms of the Dy_2S_3/C extracts obtained with different molar ratio of Dy:S:C (Linear combination of two 4.6 x 250 mm Buckyprep columns; flow rate 1.6 ml/min; toluene as eluent (mobile phase); 40°C). Fraction A is $Dy_3N@C_{80}$, fraction B is the mixture of the $Dy_2S@C_{82}$(I) and $Dy_2C_2@C_{82}$ and fraction C is $Dy_2S@C_{82}$(II)

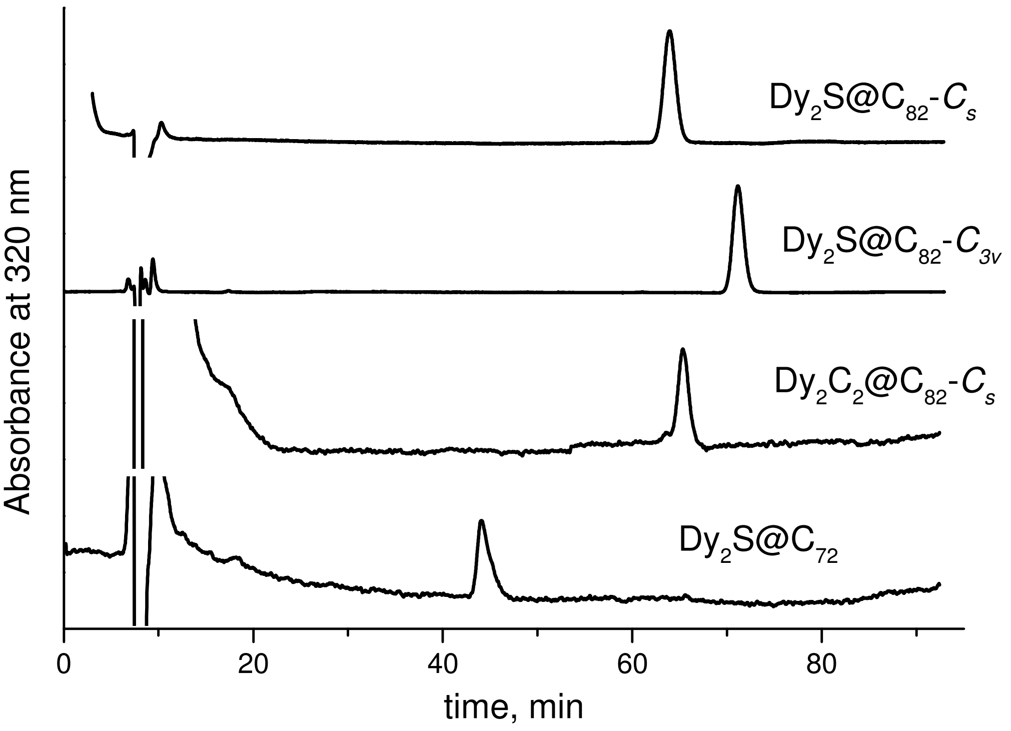

Figure S4. HPLC chromatograms of purified clusterfullerenes. Features near 10 min are due t the solvent. Besides, in the curve of $Dy_2C_2@C_{82}$-C_s, a jump of the baseline can be seen at 20 min (this region is not relevant for this work as no fullerenes elute in this range)

DFT computations of $Y_2S@C_{82}$ isomers

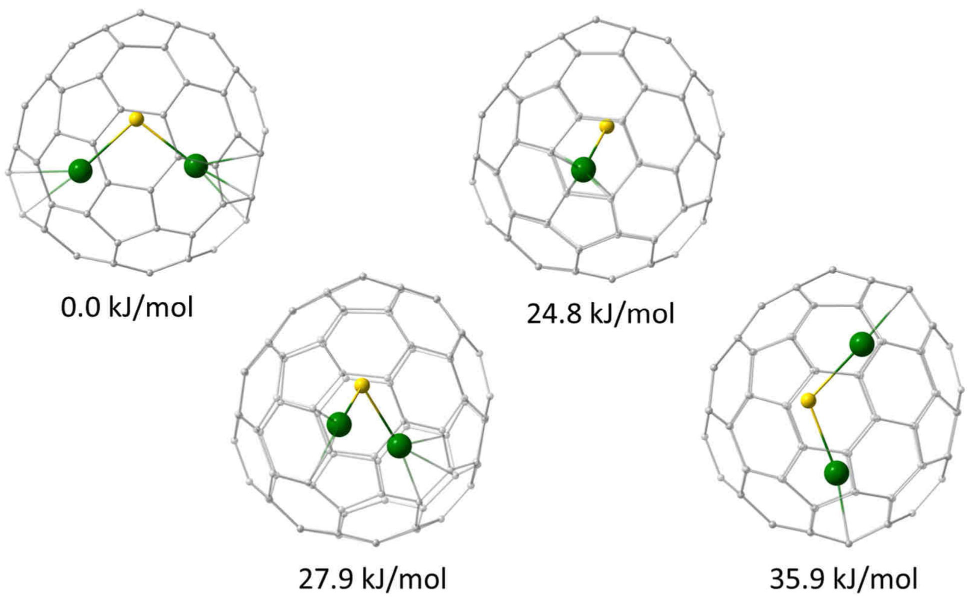

Figure S5. The lowest energy conformers of $Y_2S@C_{82}$-C_s(6) with their relative energies

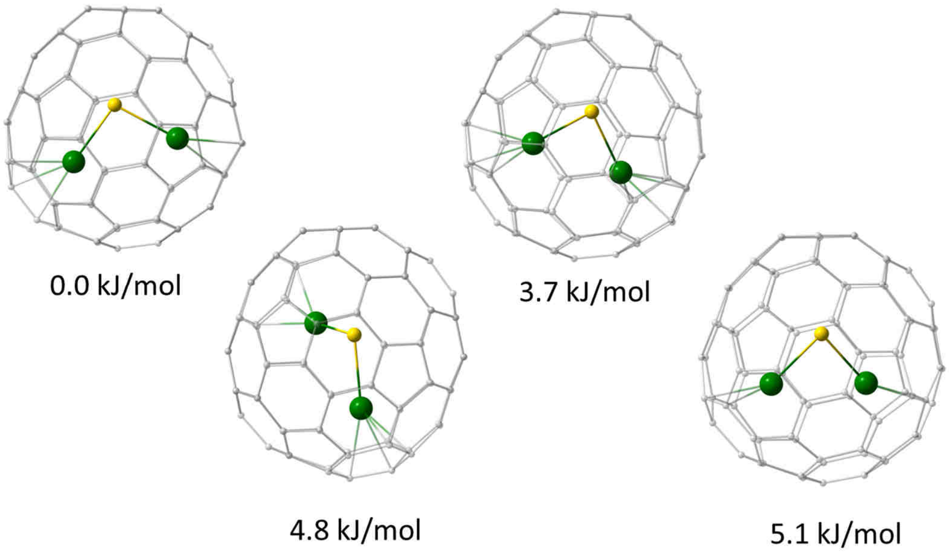

Figure S6. The lowest energy conformers of $Y_2S@C_{82}$-C_{3v}(8) with their relative energies

Table S1. Relative energies and HOMO-LUMO gaps of C_{72}^{4-}, $Y_2S@C_{72}$

		C_{72}^{4-}		$Y_2S@C_{72}$	
sym	N	E, kJ/mol	gap (eV)	E, kJ/mol	gap (eV)
C_s	10528	0.0	0.668	0.0	0.99
D_2	10611	5.8	0.484	86.4	0.34
C_s	10616	17.1	0.818	41.6	0.90
C_{2v}	11188	29.5	0.201	97.8	0.31
C_1	10610	34.8	0.540	80.1	0.63
C_1	10538	62.3	0.447	67.9	0.66
C_2	10626	61.8	0.644	133.6	0.43
C_2	10554	78.6	0.374	164.8	0.21
C_1	10557	69.4	0.494	98.7	0.48
C_2	10612	59.2	0.070	90.7	0.30

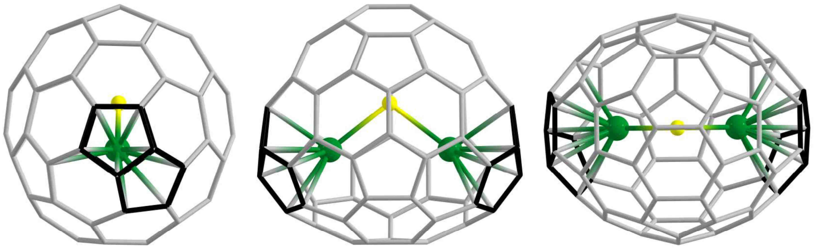

$Y_2S@C_{72}$-C_2(10528)

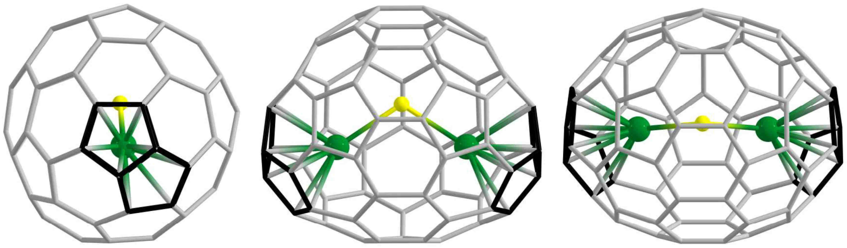

$Y_2S@C_{72}$-C_2(10616)

Figure S7. DFT-optimized molecular structure of the two most stable isomers of $Y_2S@C_{72}$.

Additional crystallographic data

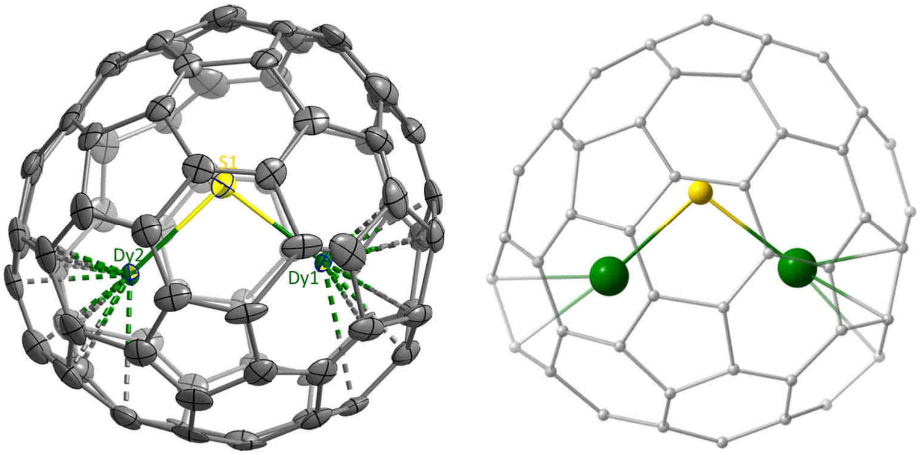

Figure S8. Left: Drawing showing one orientation of the C_s(6)-C_{82} fullerene cage with the major Dy_2S sites with 30% thermal ellipsoids. Selected geometry parameters: Dy1–S1, 2.465(5) Å; Dy2–S1, 2.518(5) Å; Dy1–S1–Dy2, 98.3(2)°. Right: the lowest energy conformer of $Y_2S@C_{82}$-C_s(6) according to DFT calculations. Selected geometry parameters: Y1–S, 2.484 Å; Y2–S, 2.506 Å; Y1–S–Y2, 99.4°.

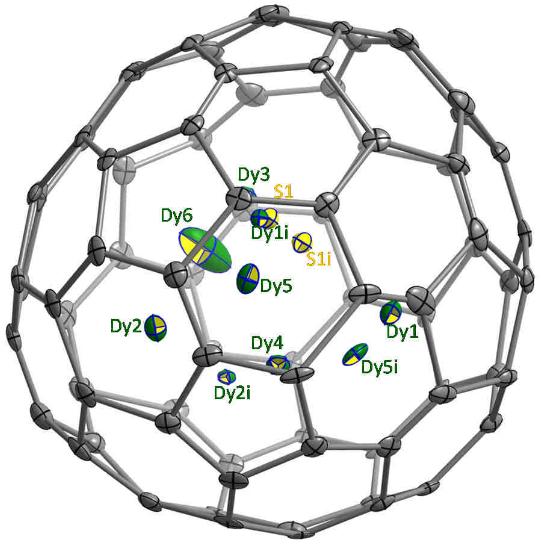

Figure S9. Drawing showing one orientation of the C_s(6)-C_{82} fullerene cage together with a half of the Dy_2S disordered sites with 10% thermal ellipsoids, another half of the Dy_2S disordered sites could be generated by the crystallographic mirror plane. The fractional occupancies are 0.5, 0.348(2), 0.3425(17), 0.113(3), 0.106(3), 0.088(2), and 0.225(4) for S1, Dy1, Dy2, Dy3, Dy4, Dy5, and Dy6, respectively. Note that Dy3, Dy4, and Dy6 locates on the crystallographic mirror plane.

As shown in Figure S9, the encapsulated Dy_2S cluster suffered severer disorders. As many as 9 sites (with occupancies 0.348(2), 0.3425(17), 0.113(3), 0.106(3), 0.088(2), and 0.225(4) for Dy1, Dy2, Dy3, Dy4, Dy5, and Dy6, respectively, another 3 sites are generated by the crystallographic mirror plane images of Dy1, Dy2 and Dy5) for the Dy are detected. However, only two sites for the S were detected, which locates on the normal position, the two S sites are symmetric according to the crystallographic mirror plane. Although there exists severer disorders for the encapsulated Dy atoms, the main sites occupied a relative high probabilities as 0.348(2) and 0.3425(17), so it's reasonable to take the Dy1-S1-Dy2 as representative for the discussion of the encapsulated Dy_2S cluster (Fig. S10).

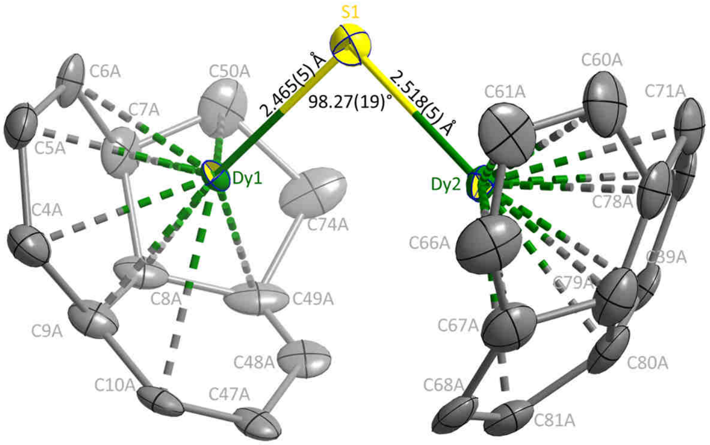

Figure S10. Major site of the Dy_2S cluster within the $C_s(6)$-C_{82} cage with X-ray determined bond lengths, bond angles and the interactions of the Dy atoms with the closest portions of the cage are shown. Displacement parameters are shown at the 30% probability level.

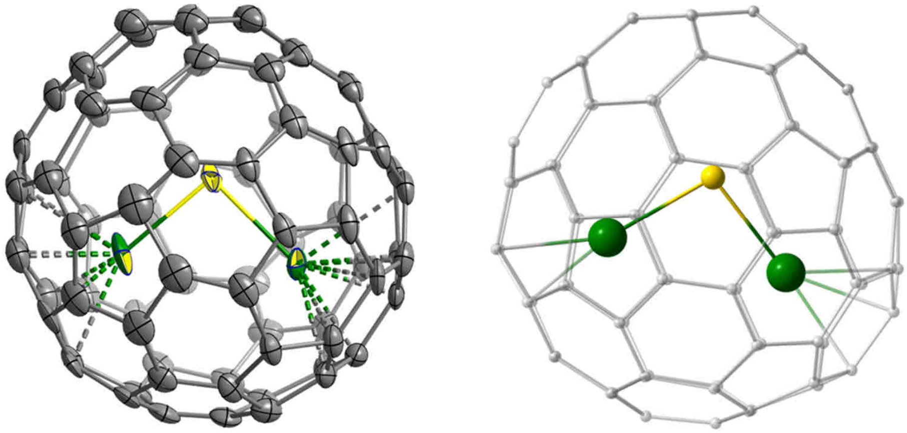

Figure S11. Left: Drawing showing one orientation of the C_{82}-C_{3v}(8) fullerene cage with the major Dy_2S site with 30% thermal ellipsoids. Selected geometry parameters: Dy2–S1, 2.437(11) Å; Dy4–S1, 2.511(9) Å; Dy2–S1–Dy4, 94.4 (2)°. Right: the lowest energy conformer of $Y_2S@C_{82}$-C_{3v}(8) according to DFT calculations. Selected geometry parameters: "Y2"–S, 2.486 Å; "Y4"–S, 2.509 Å; Y1–S–Y2, 97.2°.

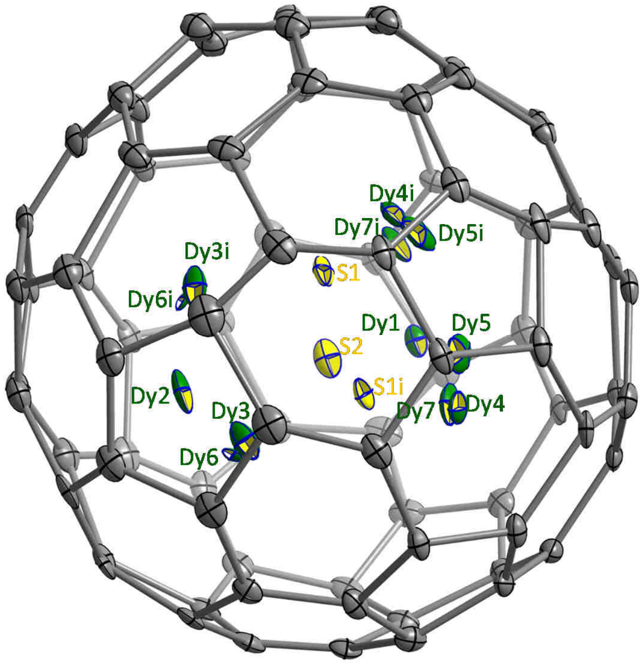

Figure S12. Drawing showing one orientation of the C_{82}-C_{3v}(8) fullerene cage together with the Dy_2S disordered sites with 10% thermal ellipsoids.

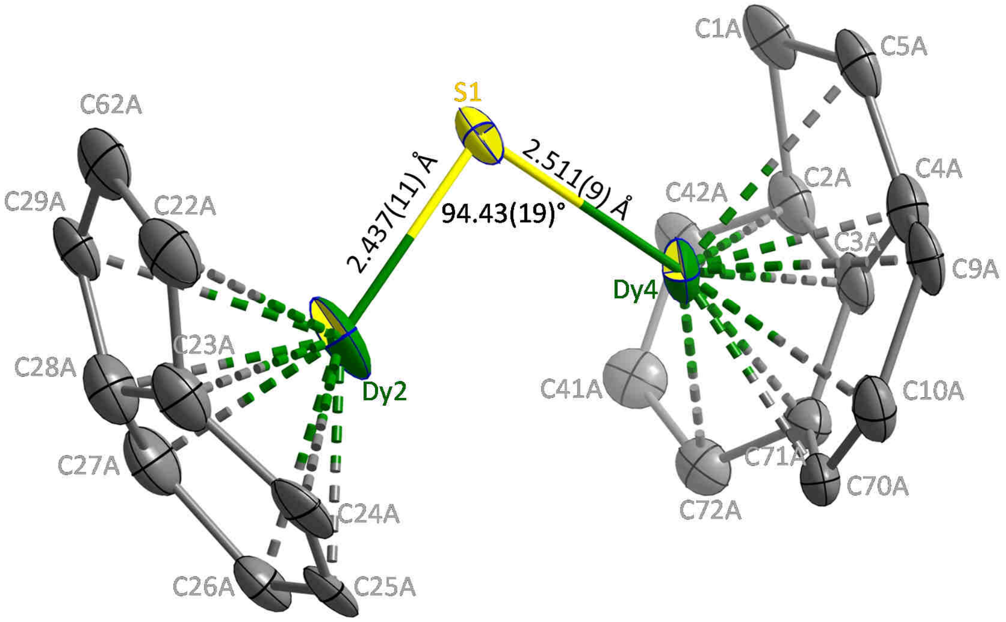

Figure S13. Major site of the Dy_2S cluster within the C_{3v}(8)-C_{82} cage with X-ray determined bond lengths, bond angles and the interactions of the Dy atoms with the closest portions of the cage are shown. Displacement parameters are shown at the 30% probability level.

Table S2. Crystal data

Crystal	$Dy_2S@C_s(6)-C_{82}\cdot Ni^{II}(OEP)\cdot 2C_7H_8$	$Dy_2S@C_{3v}(8)-C_{82}\cdot Ni^{II}(OEP)\cdot 2C_7H_8$
Formula	C132 H59 Dy2 N4 Ni S	C132 H59 Dy2 N4 Ni S
Formula weight	2116.60	2117.61
Color, habit	Black, block	Black, block
Crystal system	monoclinic	monoclinic
Space group	*C 2/m*	*C 2/m*
a, Å	25.340(5)	25.250(5)
b, Å	14.770(3)	14.860(3)
c, Å	20.550(4)	20.460(4)
α, deg	90	90
β, deg	97.13(3)	97.74(3)
γ, deg	90	90
Volume, $Å^3$	7632(3)	7607(3)
Z	4	4
T, K	100	100
Radiation (λ, Å)	Synchrotron Radiation (0.89429)	Synchrotron Radiation (0.89429)
Unique data (R_{int})	9024 (0.055)	8627 (0.2753)
Parameters	1046	1082
Restraints	1283	956
Observed data ($I > 2\sigma(I)$)	5751	4462
$R1^a$ (observed data)	0.1181	0.1630
$wR2^b$ (all data)	0.3635	0.4406
CCDC NO.	1546957	1551313

[a]For data with $I > 2\sigma(I)$, $R1=1||F_O|-|F_C||/\sum|F_O|$. [b]For all data, $wR2 =\{\sum[w(F_O^2-F_C^2)^2]\})/\sum[w(F_O^2)^2]\}^{1/2}$.

Magnetic properties of Dy-EMFs: χT products

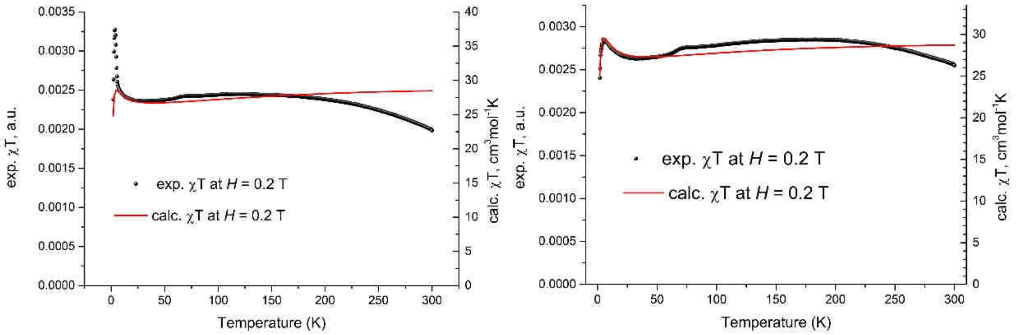

Figure S14. Experimental χT product (dots) and simulated (lines) for $Dy_2S@C_{82}$-C_s (right) and $Dy_2S@C_{82}$-C_{3v} (left). Experimental curves were partially corrected for diamagnetic contribution, but the latter still remains and leads to the decrease of the χT values at higher temperature. Sharp feature in the curve of $Dy_2S@C_{82}$-C_{3v} (left) is due to the slow relaxation at lower temperatures. The shape of the curves unambiguously points to the ferromagnetic coupling of Dy moments in the ground state.

AC measurements of $Dy_2S@C_{82}$-C_s

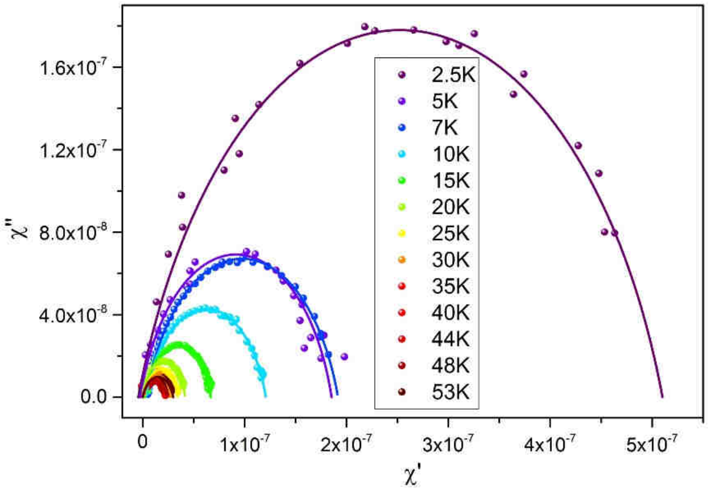

Figure S15. Cole-Cole plot for $Dy_2S@C_{82}$-C_s. Dots are experimental points, line are fit of the data with generalized Debye model

AC measurements of $Dy_2S@C_{82}$-C_{3v}

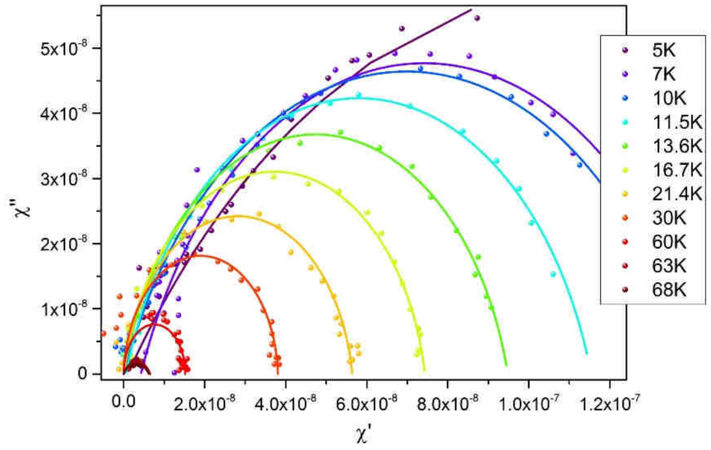

Figure S16. Cole-Cole plot for $Dy_2S@C_{82}$-C_{3v}. Dots are experimental points, line are fit of the data with generalized Debye model

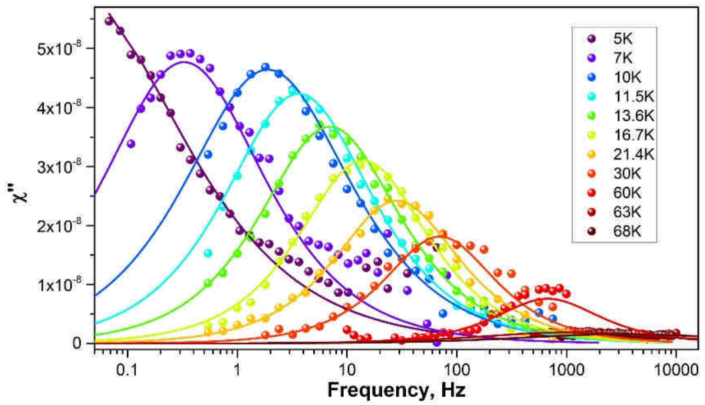

Figure S17. χ" of $Dy_2S@C_{82}$-C_{3v} measured at different temperatures as a function of AC frequency. Dots are experimental points, lines are results of the fit with generalized Debye model.

AC measurements of $Dy_2C_2@C_{82}$-C_s

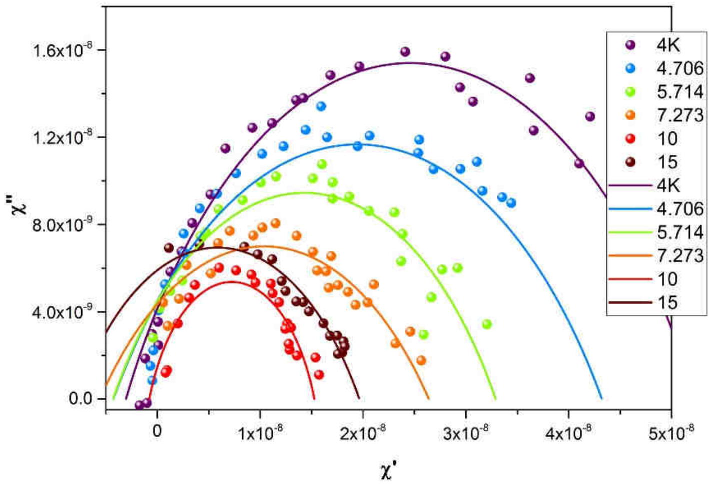

Figure S18. Cole-Cole plot for $Dy_2C_2@C_{82}$-C_s. Dots are experimental points, line are fit of the data with generalized Debye model

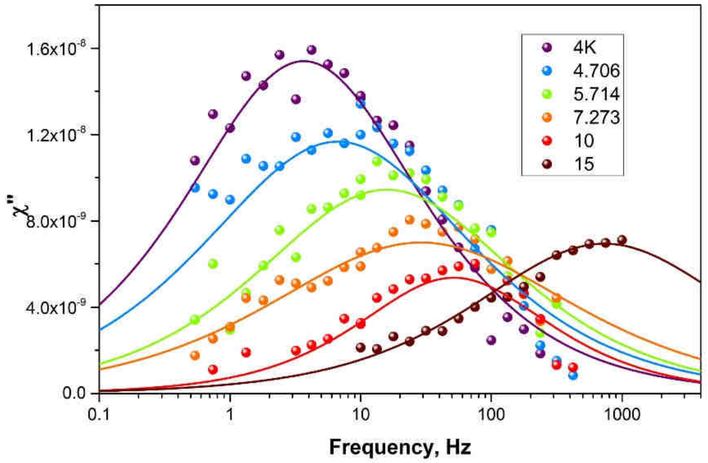

Figure S19. χ" of $Dy_2C_2@C_{82}$-C_s measured at different temperatures as a function of AC frequency. Dots are experimental points, lines are results of the fit with generalized Debye model.

Table S3a. Magnetization relaxation times of $Dy_2S@C_{82}$-C_s from DC-measurements (stretched exponential fit)

T, K	τ, sec	St-dev(τ), sec	β
1.53	50.38	0.22	0.74
1.8	29.95	0.25	0.72

Table S3b. Magnetization relaxation times of $Dy_2S@C_{82}$-C_s from AC-measurements

T, K	τ, sec	Stdev(τ), sec	α
2.5	1.64036	0.12296	0.23
3	0.47118	0.08138	0.23
3.5	0.22451	0.02218	0.19
4	0.14729	0.01306	0.23
4.5	0.07948	0.00624	0.23
5	0.05901	0.00271	0.18
7	0.02311	2.30E-04	0.21
10	0.00929	9.09E-05	0.20
15	0.0029	6.17E-05	0.15
20	0.00142	2.27E-05	0.11
25	7.72E-04	1.23E-05	0.12
30	5.24E-04	1.09E-05	0.12
35	3.36E-04	1.25E-05	0.11
40	1.39E-04	4.56E-06	0.21
41	1.18E-04	2.68E-06	0.22
42	1.00E-04	1.70E-06	0.22
43	8.20E-05	1.81E-06	0.27
44	7.00E-05	1.38E-06	0.26
45	5.90E-05	4.76E-06	0.30
46	4.69E-05	1.07E-06	0.25
47	3.71E-05	8.57E-07	0.24
48	3.04E-05	9.01E-07	0.22
49	2.51E-05	7.52E-07	0.22
50	2.20E-05	6.88E-07	0.20
52	1.44E-05	6.94E-07	0.23
53	9.09E-06	7.31E-07	0.25

Table S4a. Magnetization relaxation times of $Dy_2S@C_{82}$-C_{3v} from DC-measurements (stretched exponential fit)

T, K	τ, sec	Stdev(τ), sec	β
1.53	225.51	0.08	0.84
1.8	140.06	0.10	0.86
2	97.12	0.11	0.86
2.5	49.84	0.11	0.85
3	31.04	0.09	0.86
4	14.92	0.08	0.85

Table S4b. Magnetization relaxation times of $Dy_2S@C_{82}$-C_{3v} from AC-measurements (fitting with a generalized Debye model)

T, K	τ, sec	St-dev(τ), sec	α
6	1.27899	0.17262	0.31
7	0.48583	0.03623	0.24
8	0.23443	0.01845	0.16
9	0.13366	0.01032	0.18
10	0.08387	0.00331	0.25
11	0.05761	0.00462	0.18
11.5	0.04401	0.00111	0.19
13	0.0316	0.0014	0.09
13.6	0.02287	3.60E-04	0.16
15	0.01843	0.00115	0.06
16.7	0.01134	2.49E-04	0.11
20	0.006	9.00E-04	0.05
21.4	0.00599	1.15E-04	0.10
30	0.00227	8.58E-05	0.03
60	4.23E-04	1.80E-04	0.45
63	1.49E-04	1.17E-05	0.33
65	8.24E-05	1.23E-05	0.49
68	4.77E-05	4.55E-06	0.39
70	1.15E-05	3.88E-06	0.40

Table S5. Magnetization relaxation times of $Dy_2C_2@C_{82}$-C_s from AC-measurements (fitting with generalized Debye model)

T, K	τ, sec	St-dev(τ), sec	α
4	0.04334	0.00343	0.35
4.706	0.02387	0.00238	0.42
5.714	0.01003	8.12E-04	0.40
7.273	0.00548	8.12E-04	0.47
10	0.00308	2.85E-04	0.25
15	2.17E-04	1.28E-04	0.41

Magnetization relaxation dynamics in $Dy_2C_2@C_{82}$-C_s

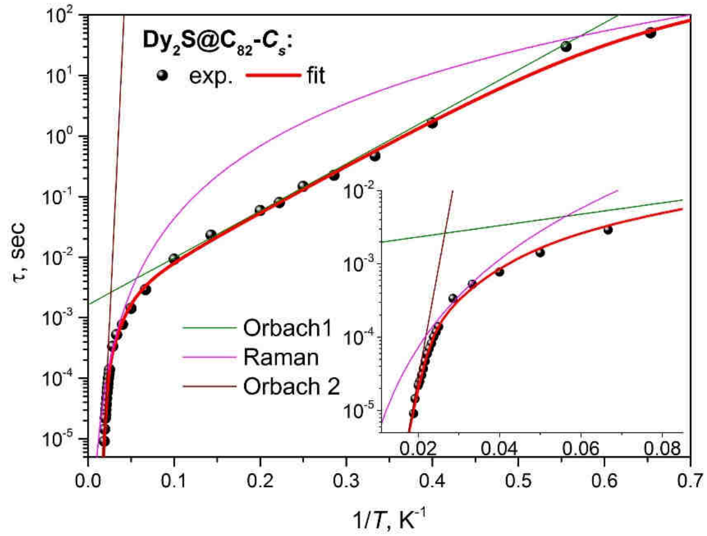

Figure S20. Magnetization relaxation times of $Dy_2C_2@C_{82}$-C_s (dots) fitted with a combination of two Orbach and one Raman relaxation processes (red line). Also shown are contributions of individual processes.

Parameters of the fit

		$Dy_2S@C_{82}$-C_s
Orbach 1	U_1^{eff}	18.0±0.5
	τ_{01}	(1.6 ± 0.2)·10^{-3}
	T_{range}	1.8–15
Raman	A	(2.5 ± 0.6)·10^{-3}
	n	3.97 ± 0.08
	T_{range}	≤1.6, 20–43
Orbach 2	U_3^{eff}	696 ± 86
	τ_{03}	(2.5 ± 4.3)·10^{-11}
	T_{range}	47–53

Magnetization relaxation dynamics in $Dy_2C_2@C_{82}$-C_{3v}

As described in the main text, magnetization relaxation times of were fitted with three Orbach processes. When full set of data on $Dy_2C_2@C_{82}$-C_{3v} was fitted with two Orbach and one Raman processes, the only possible fit was in which high-energy and intermediate Orbach processes remain almost intact, whereas the low-energy Orbach process is replaced by Raman process. Thus, the ”intermediate” Orbach process with the barrier of 50 K and untypical τ_0 value still remained as an outcome of that fit. We also tried to remove the points from the low-temperature linear regime and fit the remaining data with one Orbach and one Raman process. As shown in Fig. S21, this combination cannot provide a satisfactory description of experimental points. Besides, parameters for the high-temperature Orbach process are not realistic (U_3^{eff} of 2632 K with very large uncertainty of 7531 K). We thus conclude that the most reasonable description of the whole set of experimental data points is obtained by a combination of three Orbach processes.

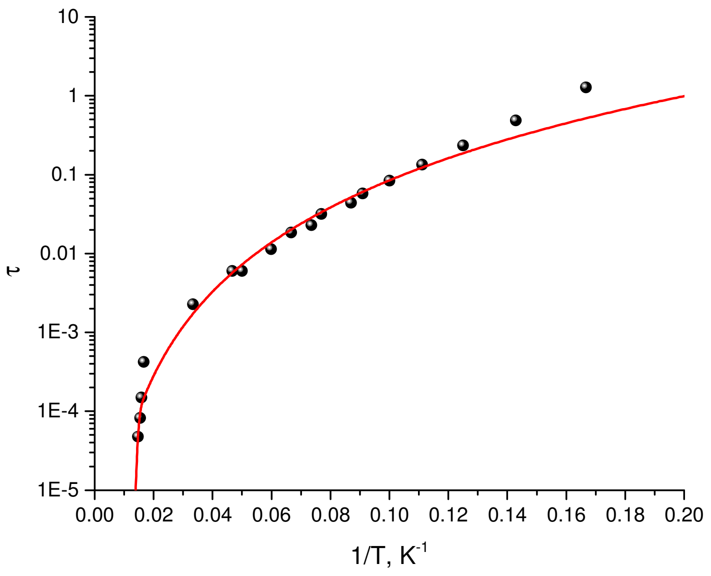

Figure S21. Magnetization relaxation times of $Dy_2C_2@C_{82}$-C_{3v} (dots) fitted with a combination of one Orbach and one Raman relaxation processes (red line). The low temperature linear regime was not included the fit.

Ab initio calculations

The next 17 pages show the results of single-ion CF calculations for individual Dy centers in a series of Dy-EMFs using the quantum chemistry package MOLCAS 8.0. Each Dy(III) atom in the system was considered independently, while the second metal in the system was f-electron free (Yttrium). Single-point complete active space self-consistent field calculations with spin-orbit interactions (CASSCF/SO-RASSI) were employed to derive ab initio values. The active space of the CASSCF calculations includes nine active electrons and seven active orbitals (e.g. CAS (9,7)). All 21 sextets, 224 quartets, and 490 doublets were included in the state-averaged CASSCF procedure. All sextets, quartets, and 350 doublets were further mixed by spin-orbit coupling in the RASSI procedure. Extended atomic natural relativistic basis sets (ANO-RCC) of VDZ-quality were used for all atoms. The single ion magnetic properties and CF-parameters were calculated with the SINGLE_ANISO module, and then used for further analysis with the PHI code.

For each Dy center, the following information is shown below

1) Table with energies and pseudospin g-tensor as calculated by SINGLE_ANISO module
2) Diagram with the energy levels and transition probabilities (the latter are coded by the lines connecting the levels – the thicker the line, the higher the probability) computed with the use of PHI code. The x axis is the projection of the g-tensor for a given state onto z axis of the ground state.
3) Description of the states in the basis of m_J vectors, also computed with the use of the PHI code.

Table S6a: $Dy_2S@C_{82}$-C_s, Dy1

state	g_x	g_y	g_z	E, cm^{-1}
1	0.0002	0.0003	19.9650	0.0
2	0.0841	0.1302	17.0652	230.7
3	0.7347	0.9324	13.5916	380.2
4	0.5223	1.7535	10.5329	507.2
5	0.9391	3.2439	8.0043	644.9
6	3.8986	5.0971	7.6777	765.1
7	1.9265	3.6483	14.6724	850.9
8	0.2946	0.5340	19.6667	912.1

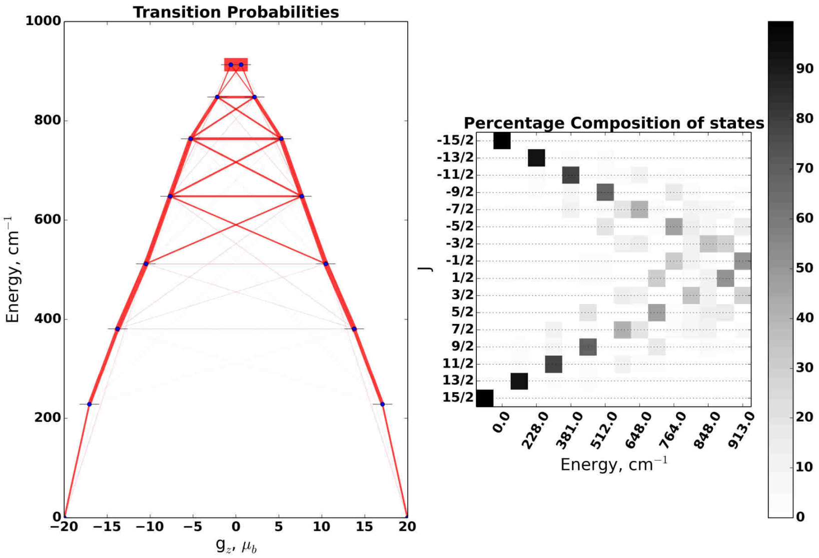

Table S6b: $Dy_2S@C_{82}$-C_s, Dy2

state	g_x	g_y	g_z	E, cm^{-1}
1	0.0032	0.0038	19.7650	0.0
2	0.0035	0.0044	17.0344	221.6
3	0.0207	0.0448	14.3340	447.4
4	0.1675	0.2221	11.3647	620.5
5	2.0854	2.2280	8.1007	721.4
6	2.4208	2.5672	5.4223	797.3
7	6.7258	5.4888	1.5485	856.9
8	0.7307	4.5589	15.8178	906.7

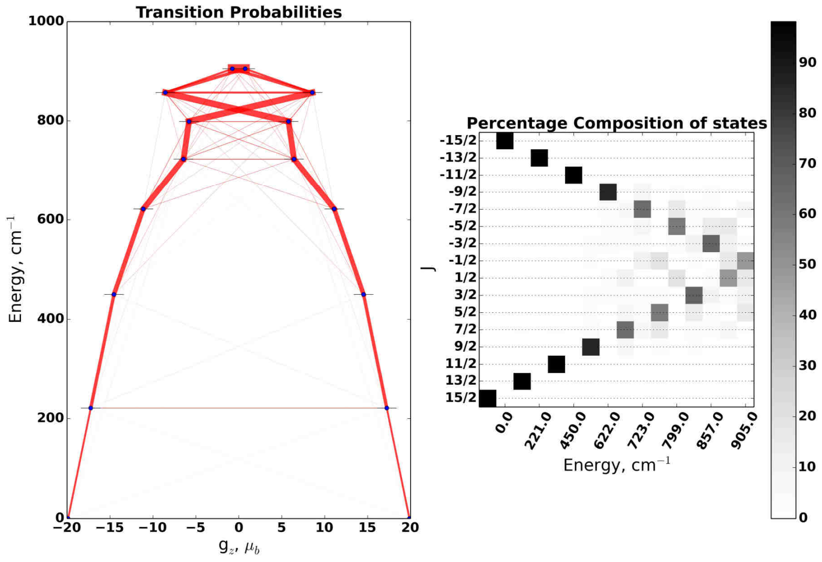

Table S7a: $Dy_2S@C_{82}$-C_{3v}, Dy1

state	g_x	g_y	g_z	*E, cm*$^{-1}$
1	0.0003	0.0005	19.8411	0.0
2	0.0251	0.0318	17.3085	267.8
3	0.3805	0.4850	14.3019	423.1
4	1.3665	1.8706	11.3274	547.2
5	2.0251	3.8422	9.8286	650.6
6	8.1833	5.8844	0.6785	740.4
7	2.8356	4.9427	11.3273	805.0
8	0.5616	2.1737	17.4047	882.2

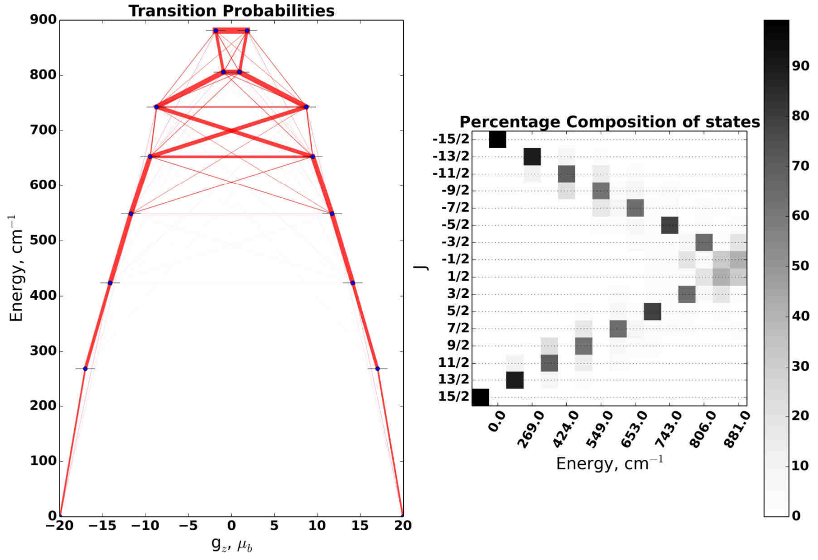

Table S7b: $Dy_2S@C_{82}$-C_{3v}, Dy2

state	g_x	g_y	g_z	*E, cm*$^{-1}$
1	0.0006	0.0007	19.8566	0.0
2	0.0229	0.0280	17.2624	293.4
3	0.3115	0.3856	14.1632	458.2
4	0.5479	1.0213	11.2205	590.4
5	2.5290	3.4277	9.4193	699.0
6	8.4728	5.0828	0.3987	786.0
7	2.1471	5.2201	12.3239	856.7
8	0.2711	0.8407	18.5977	967.9

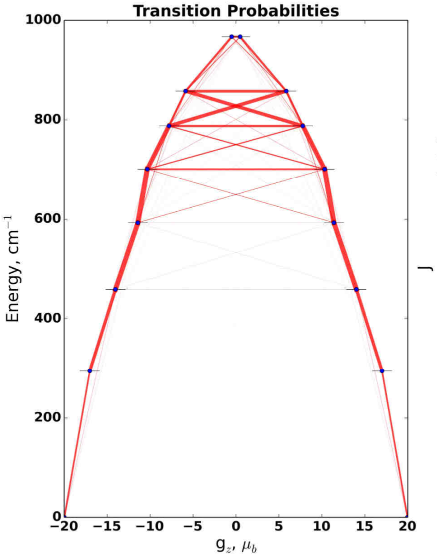

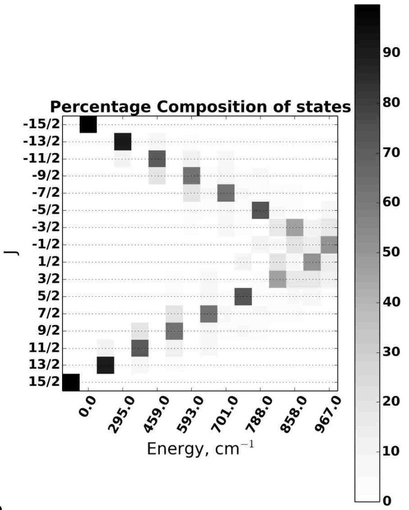

Table S8: $Dy_2S@C_{72}$-C_s, Dy1 (2)

state	g_x	g_y	g_z	*E, cm^{-1}*
1	0.0077	0.0107	19.6228	0.0
2	0.0273	0.0342	17.1878	179.8
3	0.2840	0.3472	14.6272	393.3
4	0.1340	0.8610	11.2324	569.5
5	0.8784	4.0037	8.0353	656.3
6	8.3461	5.4630	1.4531	689.8
7	0.4366	1.2023	14.3796	761.9
8	0.3081	0.9567	18.5030	877.1

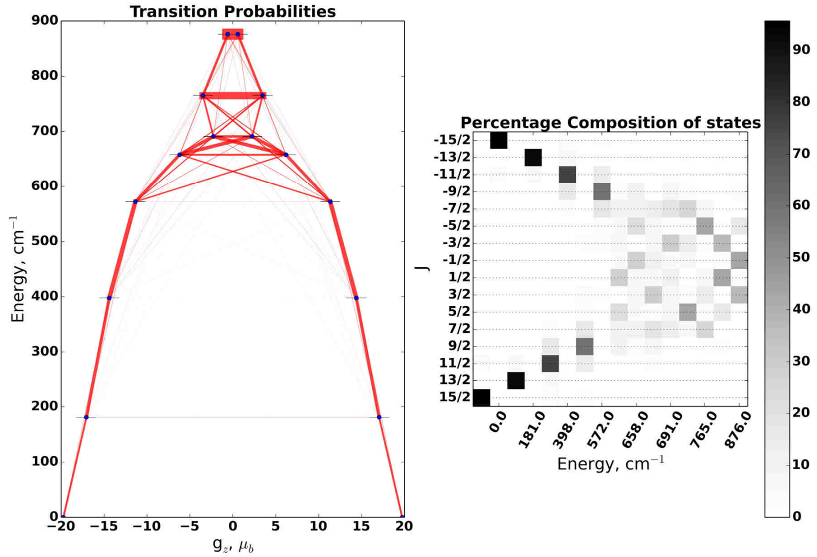

Table S9a: $Dy_2C_2@C_{82}$-C_s, Dy1

state	g_x	g_y	g_z	E, cm^{-1}
1	0.0001	0.0006	19.8733	0.0
2	0.0828	0.1247	17.2062	224.9
3	0.8722	1.0754	13.7486	353.0
4	1.3719	2.7118	10.5445	466.1
5	8.2024	4.7073	0.8863	586.1
6	1.7303	4.0732	7.1872	687.8
7	10.7800	7.4716	2.6236	743.2
8	0.4752	1.3697	18.8274	806.2

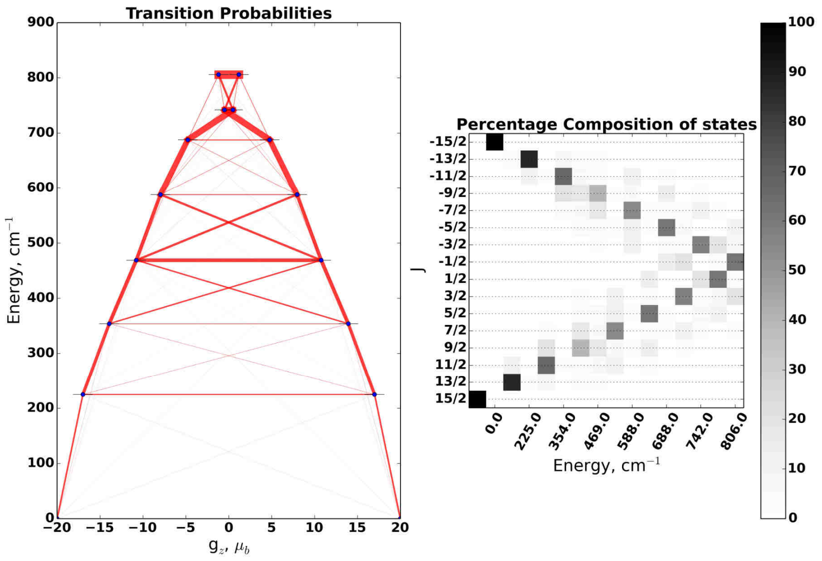

Table S9b: $Dy_2C_2@C_{82}$-C_s, Dy2

state	g_x	g_y	g_z	*E, cm*$^{-1}$
1	0.0029	0.0042	19.7548	0.0
2	0.0043	0.0063	16.9920	186.5
3	0.0972	0.1438	14.0191	390.7
4	0.3982	0.6668	11.6661	547.3
5	0.0653	1.2825	8.1660	665.8
6	0.6442	3.8172	7.0104	747.9
7	11.4802	7.2910	1.5846	791.3
8	0.2472	0.8666	18.9443	868.5

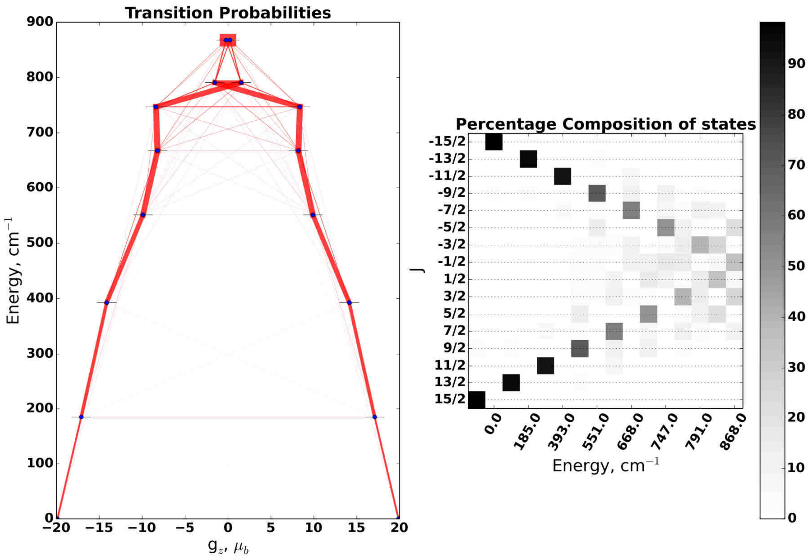

Table S10a: $Dy_2ScN@C_{80}$-I_h, Dy1

state	g_x	g_y	g_z	*E, cm*$^{-1}$
1	0.0000	0.0001	19.8785	0.0
2	0.0039	0.0043	17.1703	457.1
3	0.0691	0.0789	14.3218	754.8
4	1.1260	1.3284	11.3961	980.0
5	3.2091	4.0556	9.5690	1124.4
6	0.1966	3.8837	9.5904	1234.3
7	2.1280	4.3568	12.2452	1319.6
8	0.3556	1.1934	18.1672	1422.7

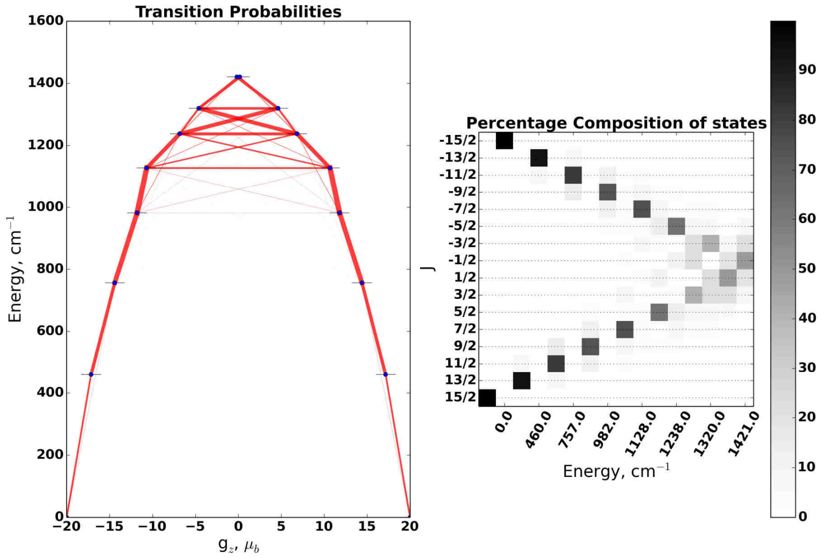

Table S10b: $Dy_2ScN@C_{80}$-I_h, Dy2

state	g_x	g_y	g_z	*E, cm^{-1}*
1	0.0001	0.0001	19.8679	0.0
2	0.0017	0.0020	17.1476	417.5
3	0.0846	0.0984	14.2677	741.3
4	0.6415	0.7382	11.4081	989.0
5	3.2286	3.3287	9.1099	1140.7
6	2.2116	3.9233	10.1780	1233.0
7	1.5964	2.7165	13.3475	1316.3
8	0.3963	1.4520	17.9062	1399.3

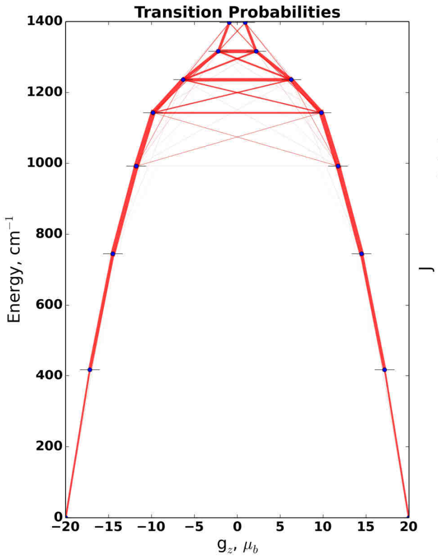

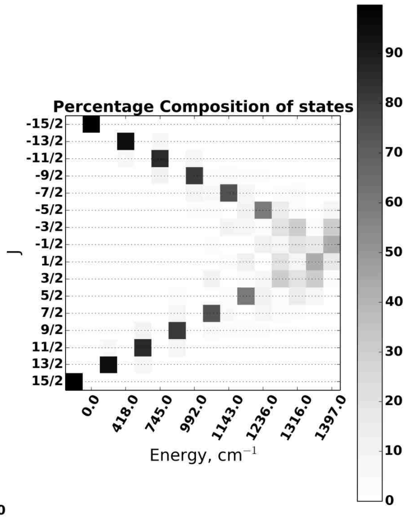

Table S11a: $Dy_2TiC@C_{80}$-I_h, Dy1

state	g_x	g_y	g_z	*E, cm^{-1}*
1	0.0005	0.0006	19.8278	0.0
2	0.0087	0.0093	17.0824	272.3
3	0.0748	0.0888	14.2896	523.6
4	0.3412	0.4182	11.7657	726.9
5	3.2420	5.1767	8.2079	856.8
6	2.0703	5.7603	9.9189	929.1
7	0.0118	0.7105	14.8941	1042.2
8	0.3165	1.0965	18.4267	1105.9

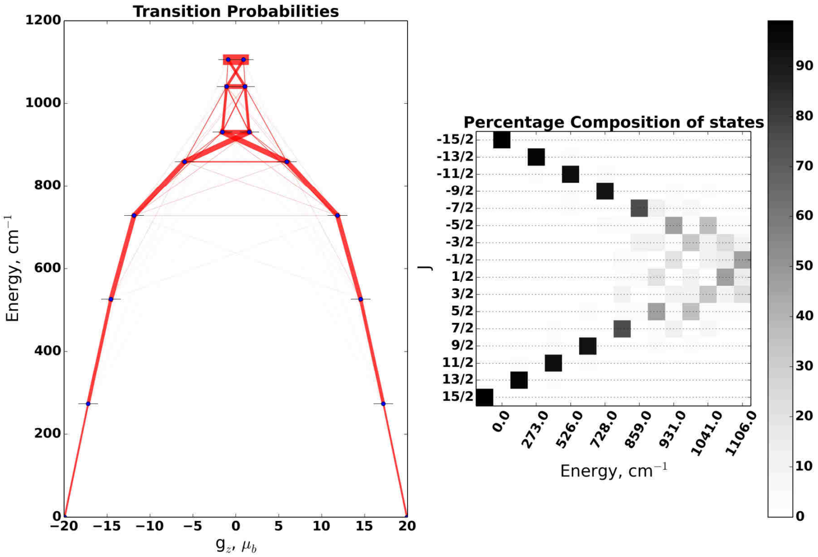

Table S11b: $Dy_2TiC@C_{80}$-I_h, Dy2

state	g_x	g_y	g_z	*E, cm^{-1}*
1	0.0001	0.0002	19.8469	0.0
2	0.0025	0.0028	17.1786	303.2
3	0.0593	0.0669	14.3630	554.3
4	0.8165	0.9521	11.5390	750.9
5	2.1178	3.1157	9.1804	883.9
6	2.8306	4.2942	9.1932	980.5
7	2.0593	2.9636	13.7880	1064.2
8	0.3815	1.2100	18.5205	1139.3

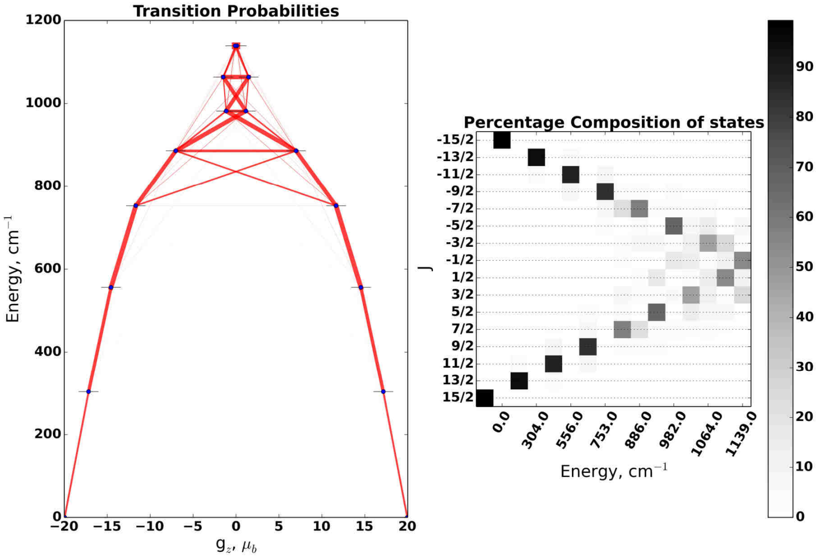

Table S12a: $Dy_2TiC_2@C_{80}$-I_h, Dy1

state	g_x	g_y	g_z	*E, cm^{-1}*
1	0.0042	0.0071	19.5749	0.0
2	0.0239	0.0315	16.9255	201.9
3	0.5713	0.8695	14.0681	429.2
4	4.3505	5.0484	11.1540	554.4
5	0.4335	3.0978	9.5535	641.2
6	1.4689	2.0459	13.9297	756.1
7	0.0984	0.1821	17.0100	880.2
8	0.0399	0.0699	19.5803	1043.9

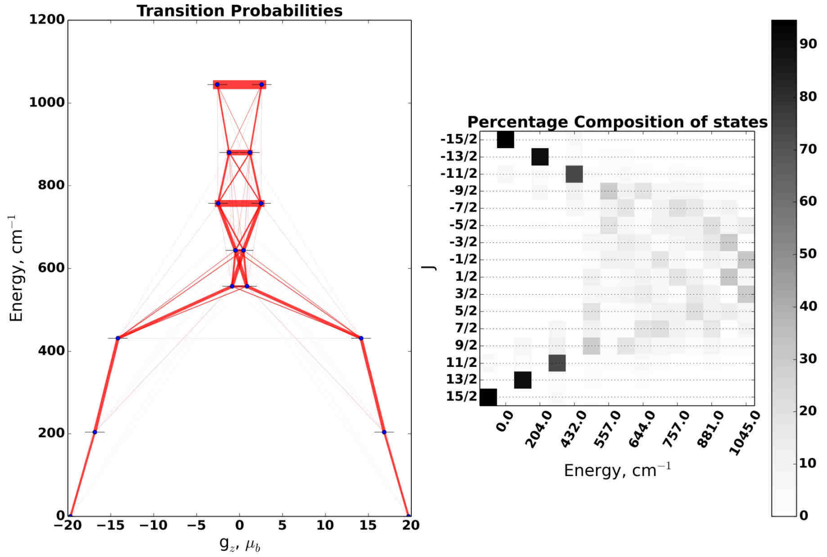

Table S12b: $Dy_2TiC_2@C_{80}$-I_h, Dy2

state	g_x	g_y	g_z	*E, cm^{-1}*
1	0.0090	0.0149	19.6211	0.0
2	0.1459	0.1971	17.1762	219.9
3	1.4283	1.7793	14.0427	397.4
4	0.8833	3.7171	11.4327	494.5
5	7.6868	7.2162	2.1518	595.9
6	3.0825	3.7779	11.8127	694.5
7	0.7290	1.1355	16.7917	806.9
8	0.0586	0.1015	19.6664	992.8

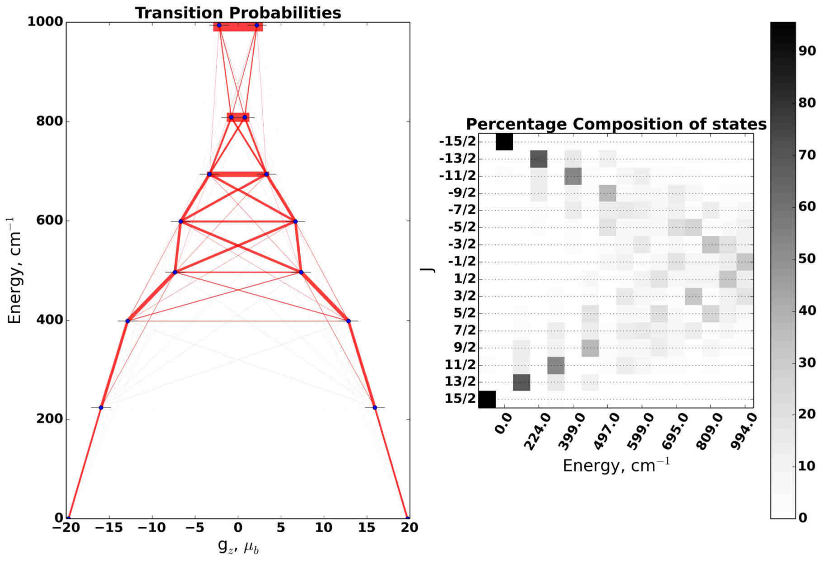

Table S13a: $Dy_2O@C_{82}$-C_{3v}, Dy1

state	g_x	g_y	g_z	*E, cm*$^{-1}$
1	0.0000	0.0001	19.8711	0.0
2	0.0013	0.0016	17.0800	431.1
3	0.0539	0.0632	14.2214	824.3
4	0.5290	0.5673	11.4596	1121.3
5	1.2017	1.7787	9.2466	1285.5
6	7.5724	6.0239	2.9820	1362.9
7	0.5283	3.1929	14.8079	1397.4
8	0.0327	2.1566	17.3251	1443.9

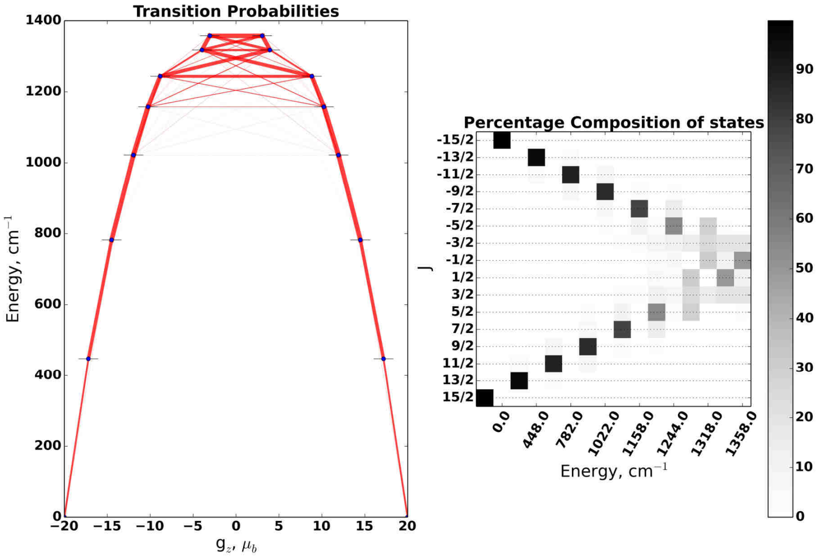

Table S13b: $Dy_2O@C_{82}$-C_{3v}, Dy2

state	g_x	g_y	g_z	*E, cm*$^{-1}$
1	0.0001	0.0001	19.8761	0.0
2	0.0017	0.0019	17.1128	448.3
3	0.0470	0.0550	14.2836	780.3
4	0.6089	0.7300	11.8772	1021.1
5	1.0687	2.5330	11.1699	1151.8
6	2.0072	5.1298	9.3784	1237.2
7	2.3687	3.5746	7.0463	1313.0
8	11.100	9.6597	1.2358	1355.6

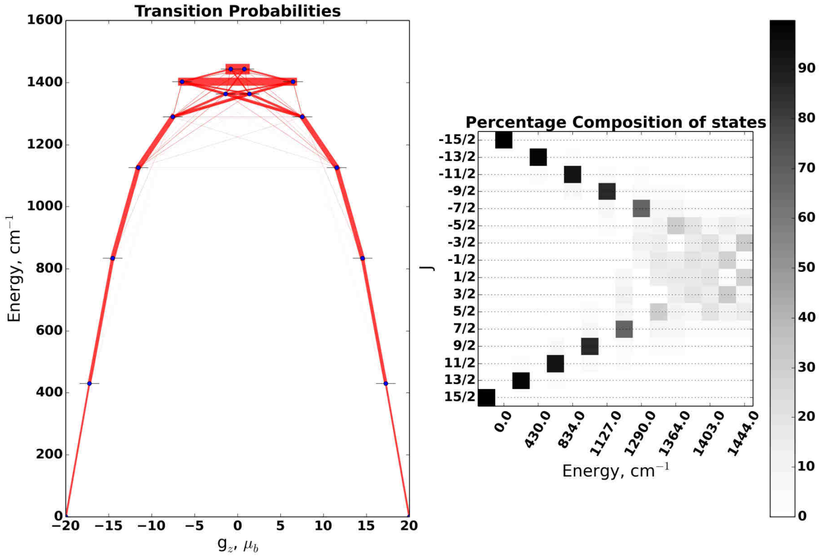

Table S14: DyNC@C_{76}, Dy1

state	g_x	g_y	g_z	*E, cm^{-1}*
1	0.0000	0.0004	19.8082	0.0
2	0.0056	0.0059	17.2831	339.4
3	0.3332	0.4173	14.3282	573.8
4	0.8143	1.3162	11.3947	740.5
5	2.4617	3.3928	10.8220	854.0
6	8.8718	8.3874	1.8679	908.8
7	2.3735	2.8798	7.6196	991.3
8	12.3544	8.2757	0.9525	1044.5

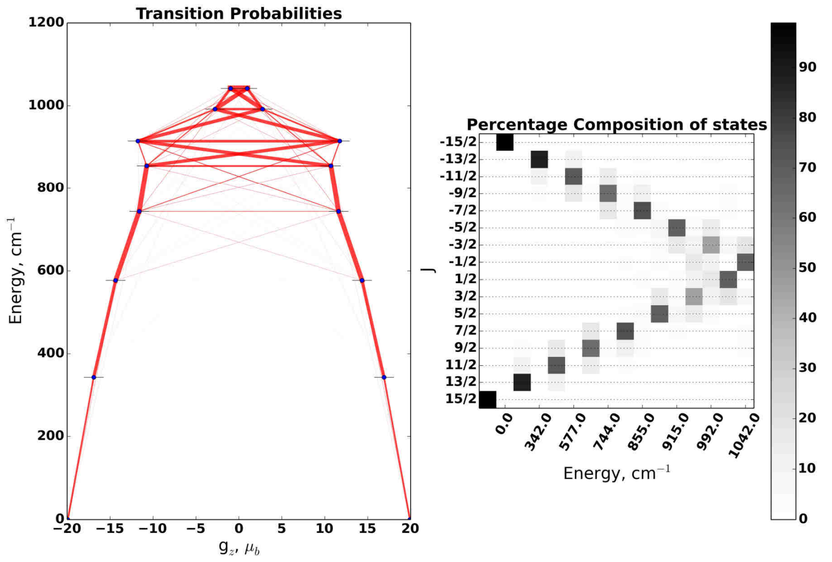

Table S15: DyNC@C_{82}-C_2, Dy1

state	g_x	g_y	g_z	*E, cm*$^{-1}$
1	0.0016	0.0026	19.8734	0.0
2	0.1267	0.1901	16.9939	247.4
3	0.7901	0.8621	13.9500	351.0
4	1.0140	2.6629	11.4908	426.9
5	8.7465	5.5293	1.8069	517.5
6	8.7062	5.2576	1.5912	607.3
7	2.0194	2.5119	11.8006	688.0
8	0.6700	2.6061	17.4310	743.7

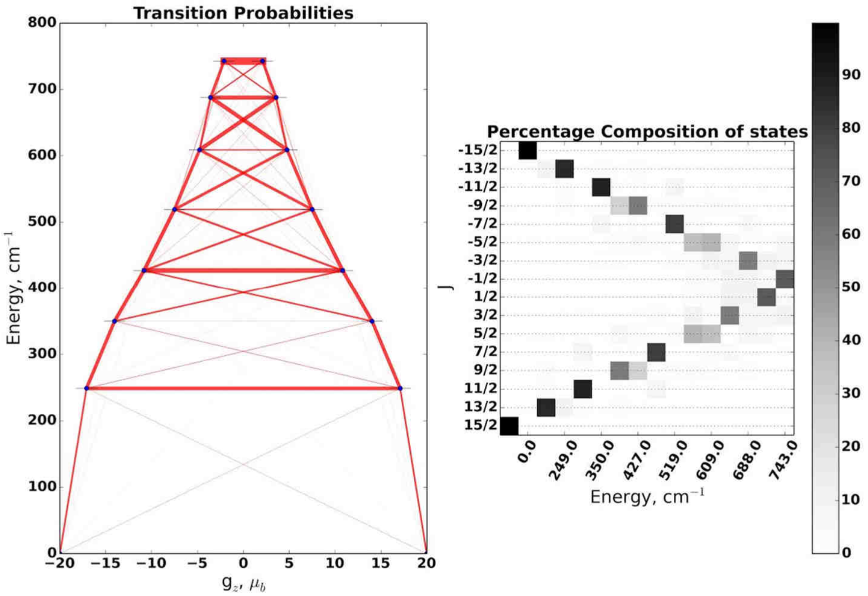

Ab initio calculations with Molpro

To evaluate the influence of dynamic correlation, a series of additional *ab initio* calculations were performed for selected EMFs using the quantum chemistry package MOLPRO [1]. Only the endohedral cluster was considered in *ab initio* calculations, whereas the carbon atoms of the fullerene cage were substituted by a set of point charges. It was suggested that the formal charge of the cage (e.g., −4 for $Dy_2S@C_{82}$, or −6 for $Dy_2ScN@C_{80}$, or −2 for $DyNC@C_{82}$) was distributed uniformly between all carbon atoms. One Dy (III) atom was explicitly considered as f^9 site while the second f-electron site was substituted by a closed shell Lu^{3+} (f^{14}) ion. The Dy and Lu sites were described by energy-consistent relativistic pseudopotentials along with valence basis sets of quintuple-zeta quality [2]. All-electron basis sets of triple-zeta quality were used for carbon [3], nitrogen [3], sulphur [4], titanium [5] or scandium [5].

CASSCF calculations with nine electrons and seven 4f orbitals in the active space were used to generate multiconfiguration wave functions in the initial step. The CASSCF optimization was carried out for all 21 sextets and low-lying 39 quartets. Both at the CASSCF and MRCI [6,7] level, 18 sextets and 8 quartets were considered in the spin-orbit calculations. The spin-orbit treatement was carried out as described in Ref. [8]. The 4f, 5s, 5p orbitals of the lanthanides and all orbitals of the outer-most shell for the other atoms in the cluster were correlated in the MRCI-SD treatement. Dy f^9 g-factors were computed following the approach proposed by Bolvin [9] (see also Refs. [10,11]).

In the following tables we provide energies (in cm^{-1}) for the lowest four Kramers doublets obtained by both CASSCF and MRCI spin-orbit calculations. g factors associated with each pair are also provided in the corresponding row. We focused on the low-lying excited states below ~600 cm^{-1}, where we find reasonable agreement at the CASSCF level with "full-molecule" computations carried out using MOLCAS. For the relative energies of these excited states, we find correlation-induced corrections of up to 10-15% at the MRCI-SD level. The effect of the MRCI-SD treatment is however minor in the case of the g factors for the ground and the first excited states.

[1] H. J. Werner, P. J. Knowles, G.Knizia, F. R. Manby & M.Schütz "Molpro: a general-purpose quantum chemistry program package." Wiley Rev: Comp. Mol. Sci. 2, 242–253 (2012).

[2] X. Cao, M. Dolg, "Valence basis sets for relativistic energy-consistent small-core lanthanide pseudopotentials" J. Chem. Phys. 115, 7348 (2001)

[3] T.H. Dunning Jr., "Gaussian basis sets for use in correlated molecular calculations. I. The atoms boron through neon and hydrogen" J. Chem. Phys. 90, 1007 (1989)

[4] D.E. Woon and T.H. Dunning Jr., "Gaussian basis sets for use in correlated molecular calculations. III. The atoms aluminum through argon" J. Chem. Phys. 98, 1358 (1993).

[5] N.B. Balabanov and K.A. Peterson, "Systematically convergent basis sets for transition metals. I. All-electron correlation consistent basis sets for the 3d elements Sc-Zn" J. Chem. Phys. 123, 064107 (2005)
[6] H.-J. Werner & P. J. Knowles An efficient internally contracted multiconfiguration-reference configuration interaction method. J. Chem. Phys. 89, 5803–5814 (1988).
[7] P. J. Knowles & H.-J. Werner Internally contracted multiconfiguration-reference configuration interaction calculations for excited states. Theor. Chim. Acta 84, 95–103 (1992).
[8] A. Berning, M. Schweizer, H.-J. Werner, P. J. Knowles & P. Palmieri Spin-orbit matrix elements for internally contracted multireference configuration interaction wavefunctions. Mol. Phys. 98, 1823–1833 (2000).
[9] H. Bolvin, An alternative approach to the g-matrix: Theory and applications. Chem. Phys. Chem. **7**, 1575 (2006).
[10] N. A . Bogdanov. et al. . Orbital reconstruction in nonpolar tetravalent transition-metal oxide layers. Nat. Commun. 6, 7306 (2015)
[11] R. Yadav, N. A. Bogdanov, V. M. Katukuri, S. Nishimoto, J. van den Brink, and L. Hozoi, "Kitaev exchange and field-induced quantum spin-liquid states in honeycomb α-RuCl3 ," Sci. Rep. 6, 37925 (2016).

Table S16. The energies (in cm^{-1}) of KDs for Dy1 in $Dy_2C_2@C_{82}$-C_s computed using CASSCF and MRCI approaches. Also shown the set of energies from full molecule CASSCF calculations

CAS, full	CAS(18s15q)	CAS(18s8q)	MRCI(18s8q)
0	0	0	0
225	203.4	203.5	238.3
354	375.2	378.8	433.3
469	525.9	535.7	598.8

It can be seen that the CAS(18s15q) approach with point charges instead of carbon atoms gives reasonable agreement with full-molecule computations.

Table S17a: Dy_2C_2@C_{82}-C_s, Dy1 **CASSCF**

state	g_{xx}	g_{yy}	g_{zz}	E, cm
1	0.0000	0.0000	19.9351	0.0
2	0.0002	0.0002	17.1821	203.4
3	0.0040	0.0039	14.4731	375.2
4	0.2043	0.3952	11.7504	525.9

Table S17b: Dy_2C_2@C_{82}-C_s, Dy1 **MRCI**

state	g_{xx}	g_{yy}	g_{zz}	E, cm
1	0.0000	0.0000	19.9316	0.0
2	0.0002	0.0001	17.1617	238.3
3	0.0333	0.0269	14.3932	433.3
4	0.1479	0.1054	11.7391	598.8

Table S18a: $Dy_2ScN@C_{80}$-$\boldsymbol{I_h}$, Dy1 **CASSCF**

state	g_{xx}	g_{yy}	g_{zz}	E, cm
1	0.0006	0.0004	19.9011	0.0
2	0.0005	0.0017	16.9852	408.4

Table S18b: $Dy_2ScN@C_{80}$-$\boldsymbol{I_h}$, Dy1 **MRCI**

state	g_{xx}	g_{yy}	g_{zz}	E, cm
1	0.0001	0.0008	19.8913	0.0
2	0.0012	0.0011	16.9717	443.4

Table S19a: $Dy_2TiC@C_{80}$-$\boldsymbol{I_h}$, Dy1 **CASSCF**

state	g_{xx}	g_{yy}	g_{zz}	E, cm
1	0.0018	0.0022	19.9419	0.0
2	0.0217	0.0203	17.1158	310.5

Table S19b: $Dy_2TiC@C_{80}$-$\boldsymbol{I_h}$, Dy1 **MRCI**

state	g_{xx}	g_{yy}	g_{zz}	E, cm
1	0.0011	0.0017	19.9319	0.0
2	0.0228	0.0197	17.0918	343.7

Broken-symmetry calculations of di-Gd analogs

Exchange coupling parameters j_{12}^{ex}(Gd-Gd) in Gd-EMF analogs of di-Dy EMFs were computed using Orca code at the PBE0/TZVP-DKH level within the broken-symmetry approximation, they correspond to the Hamiltonian:

$$H = -2j_{12}S_1 \cdot S_2$$

Table S20. Exchange coupling parameters in Gd analogs of di-Dy EMFs.

	j_{12}^{ex}(Gd-Gd), cm^{-1}
$Gd_2S@C_{72}$	0.13
$Gd_2S@C_{82}$-C_s	1.24
$Gd_2S@C_{82}$-C_{3v}	0.64
$Gd_2C_2@C_{82}$-C_s	0.68
$Gd_2ScN@C_{80}$	0.55
$Gd_2TiC@C_{80}$	0.67
$Gd_2TiC_2@C_{80}$	0.46
$Gd_2O@C_{82}$-C_{3v}	0.31

Energy spectrum of the total spin Hamiltonian

Excited states of the di-Dy systems in the low-energy part of the spectrum are roughly additive of the values of the constituent single-ions. That is, 4 states with $J_z = \pm 15/2$ for both Dy ions in FM and AFM arrangement, $\left|\pm 15/2, \pm 15/2\right\rangle$, are followed by 8 $\left|\pm 15/2, \pm 13/2\right\rangle$ and $\left|\pm 13/2, \pm 15/2\right\rangle$ states, in which one of the Dy centers has $J_z = \pm 13/2$. For instance, in $Dy_2S@C_{82}$-C_s, the energies of mixed $\left|\pm 15/2, \pm 13/2\right\rangle$ and $\left|\pm 13/2, \pm 15/2\right\rangle$ states span the range of 213–234 cm^{-1} (310–340 K), which matches the energies of CF states of single ions (see Table 2) with an addition of the energy difference between FM and AFM coupled systems. The density of states is then increasing dramatically at higher energies as more and more mixed states become available in the middle part of the spectrum (Fig. S22).

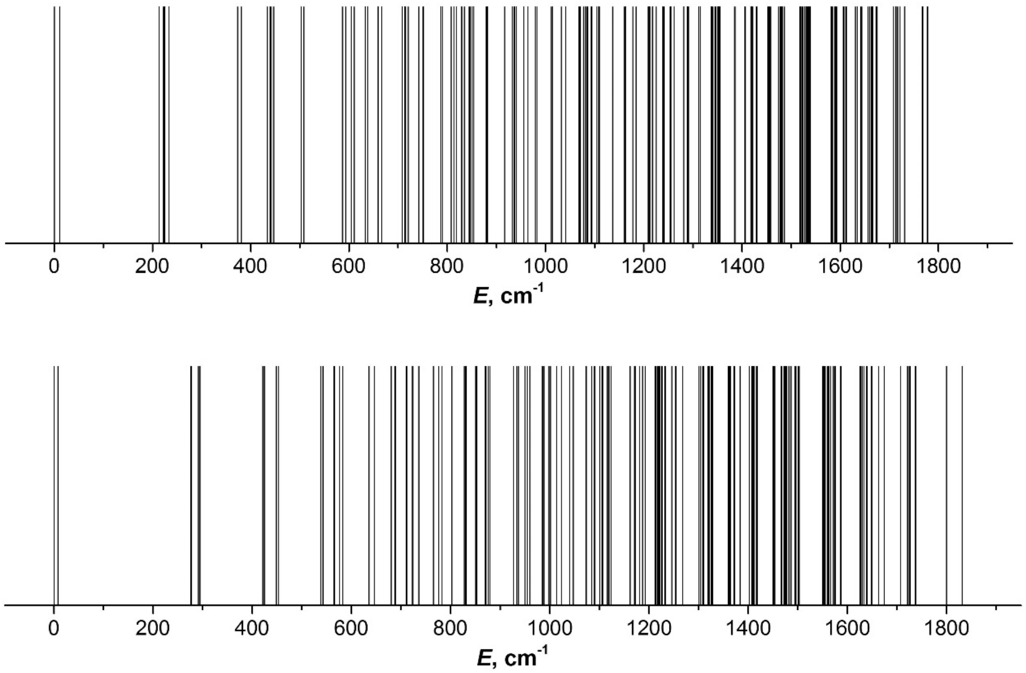

Figure S22. Energy spectrum of the total spin Hamiltonian for $Dy_2S@C_{82}$-C_s (top) and $Dy_2S@C_{82}$-C_{3v} (bottom) Dy_2 is not a Kramers system, but each vertical line in the spectra corresponds to *de facto* to a doublet state.

Magnetization curves at T = 3K: experiment versus simulation

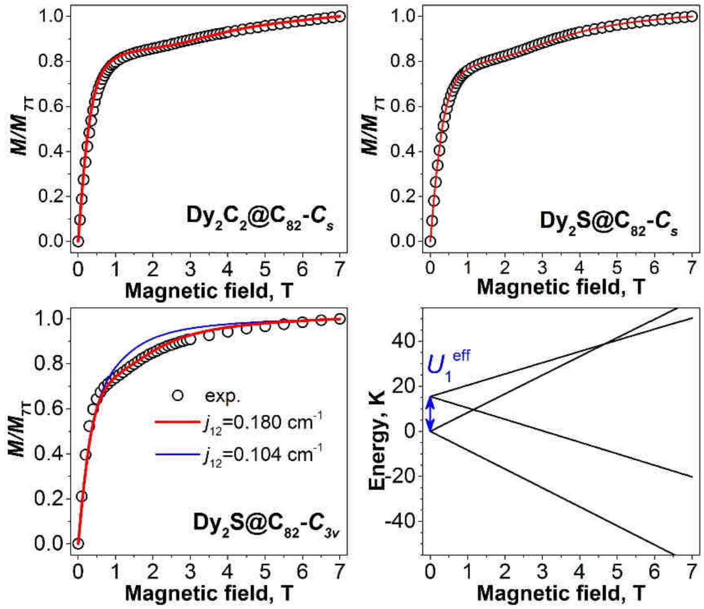

Figure S23. Experimental (dots) and simulated (lines) magnetization curves of $Dy_2C_2@C_{82}$-C_s, $Dy_2S@C_{82}$-C_s, and $Dy_2S@C_{82}$-C_{3v} at 3 K (for $Dy_2S@C_{82}$-C_{3v} the measurements were performed with very slow sweep rate to ensure that the curve is close to the thermodynamic limit). For $Dy_2S@C_{82}$-C_{3v}, two simulated curves corresponding to different j_{12} values are shown. Also shown is Zeeman energy splitting in $Dy_2S@C_{82}$-C_s for an arbitrary orientation of the magnetic field.

DFT-optimized Cartesian coordinates of the molecules used in ab initio calculations

Table S21. $Dy_2S@C_{72}$

C	-2.339852001	-0.100260236	-4.215908628
C	-1.518086717	1.086225037	-4.131868008
C	-1.449647383	-1.245074501	-4.087308824
C	-0.140035930	0.712939205	-3.949637468
C	-0.078570141	-0.747923960	-3.937101635
C	-3.497615023	0.087406361	-3.339331847
C	-2.129896226	2.028496604	-3.231718089
C	-1.851101944	-2.303904901	-3.216180046
C	0.916542825	-1.428897288	-3.121904642
C	0.727818731	1.504289595	-3.107431636
C	-3.384534406	1.442077500	-2.772436922
C	-3.093250307	-2.207740933	-2.448013780
C	-3.882964392	-0.989834587	-2.440673267
C	1.975772375	-0.623895115	-2.616018067
C	1.880233327	0.831798545	-2.605434905
C	-0.879399745	-3.007197054	-2.411483803
C	-1.313683953	2.833121330	-2.356487358
C	0.512066424	-2.626718217	-2.364896932
C	0.151070591	2.633164439	-2.342111063
C	-3.896186644	1.773527254	-1.462240676
C	-2.898859290	-2.973090393	-1.230475610
C	-1.551409580	-3.472987184	-1.224967906
C	2.716500673	-1.018363988	-1.432629322
C	2.565582265	1.303193880	-1.425912711
C	-4.475075304	-0.631028316	-1.171150866
C	-1.959777266	3.311339300	-1.173744778
C	1.231619050	-2.964792610	-1.180485688
C	0.864291258	3.066698073	-1.185035698
C	3.097160129	0.165305479	-0.703192519
C	-4.550471552	0.736820882	-0.719980860
C	-3.232013765	2.795511459	-0.727276233
C	2.341620431	-2.168692514	-0.726734514
C	2.057303649	2.399924907	-0.730322173
C	0.554452967	-3.460134545	0.000000000
C	0.174792721	3.526611733	0.000000000
C	-0.847972394	-3.676401137	0.000000000
C	-1.207139027	3.646091297	0.000000000
C	-3.542610290	-2.630641019	0.000000000
C	-4.372517600	-1.465183320	0.000000000
C	2.057303649	2.399924907	0.730322173
C	2.341620431	-2.168692514	0.726734514
C	-3.232013765	2.795511459	0.727276233
C	-4.550471552	0.736820882	0.719980860
C	3.097160129	0.165305479	0.703192519
C	0.864291258	3.066698073	1.185035698
C	1.231619050	-2.964792610	1.180485688
C	-1.959777266	3.311339300	1.173744778
C	-4.475075304	-0.631028316	1.171150866
C	2.565582265	1.303193880	1.425912711
C	2.716500673	-1.018363988	1.432629322
C	-1.551409580	-3.472987184	1.224967906
C	-2.898859290	-2.973090393	1.230475610
C	-3.896186644	1.773527254	1.462240676

C	0.151070591	2.633164439	2.342111063
C	0.512066424	-2.626718217	2.364896932
C	-1.313683953	2.833121330	2.356487358
C	-0.879399745	-3.007197054	2.411483803
C	1.880233327	0.831798545	2.605434905
C	1.975772375	-0.623895115	2.616018067
C	-3.882964392	-0.989834587	2.440673267
C	-3.093250307	-2.207740933	2.448013780
C	-3.384534406	1.442077500	2.772436922
C	0.727818731	1.504289595	3.107431636
C	0.916542825	-1.428897288	3.121904642
C	-1.851101944	-2.303904901	3.216180046
C	-2.129896226	2.028496604	3.231718089
C	-3.497615023	0.087406361	3.339331847
C	-0.078570141	-0.747923960	3.937101635
C	-0.140035930	0.712939205	3.949637468
C	-1.449647383	-1.245074501	4.087308824
C	-1.518086717	1.086225037	4.131868008
C	-2.339852001	-0.100260236	4.215908628
Dy	-1.496613022	-0.096381425	1.944419507
Dy	-1.496613022	-0.096381425	-1.944419507
S	0.000000000	0.000000000	0.000000000

Table S22. $Dy_2S@C_{82}$-C_s

C	2.567327651	2.696678762	3.191524641
C	2.968786865	1.457971496	3.745152924
C	4.003365384	0.719633681	3.084317225
C	1.974511812	0.716335067	4.475041218
C	0.610928564	1.173423171	4.670115890
C	0.175472105	2.359027339	3.944579822
C	1.185080896	3.131905072	3.297901257
C	1.974511812	-0.716335067	4.475041218
C	2.968786865	-1.457971496	3.745152924
C	4.003365384	-0.719633681	3.084317225
C	2.567327651	-2.696678762	3.191524641
C	-1.564006681	0.000000000	4.282713818
C	-0.236336425	0.000000000	4.872880231
C	0.610928564	-1.173423171	4.670115890
C	0.175472105	-2.359027339	3.944579822
C	1.185080896	-3.131905072	3.297901257
C	-1.440679311	3.154261991	2.217547605
C	-1.182957212	2.363392742	3.370620319
C	-1.999116183	1.169792497	3.526627288
C	3.082205507	-3.135587485	1.921032171
C	4.029642451	-2.347600023	1.210458573
C	4.526094251	-1.162413416	1.826660732
C	3.969481916	-2.347585419	-0.222437110
C	-0.389586786	-3.909626850	1.583134473
C	0.895985093	-3.886010753	2.109342516
C	2.023557996	-3.838133022	1.225499266
C	1.841515868	-3.706605357	-0.180454678
C	2.870811796	-3.000949892	-0.885916405
C	-1.999116183	-1.169792497	3.526627288
C	-1.182957212	-2.363392742	3.370620319
C	-1.440679311	-3.154261991	2.217547605
C	4.446599972	-1.248015526	-1.053495514
C	4.880014087	0.000000000	-0.418734026
C	4.870516109	0.000000000	1.023410400
C	4.446599972	1.248015526	-1.053495514
C	3.583639234	-1.237354387	-2.243154204
C	1.870520540	0.000000000	-3.487053168
C	3.150055582	0.000000000	-2.860099837
C	3.583639234	1.237354387	-2.243154204
C	1.310229491	-2.307805704	-2.698378892
C	2.621511988	-2.319677287	-2.111821066
C	1.013717968	-1.176098268	-3.509032783
C	0.231084489	-3.028438371	-2.019990591
C	-0.587687765	-3.903654414	0.160560271
C	0.496215137	-3.720542013	-0.750074901
C	-1.105580126	-2.590905233	-2.275725719
C	3.969481916	2.347585419	-0.222437110
C	4.029642451	2.347600023	1.210458573
C	4.526094251	1.162413416	1.826660732
C	3.082205507	3.135587485	1.921032171
C	2.870811796	3.000949892	-0.885916405
C	1.841515868	3.706605357	-0.180454678
C	2.023557996	3.838133022	1.225499266
C	-0.389586786	3.909626850	1.583134473
C	0.895985093	3.886010753	2.109342516
C	1.013717968	1.176098268	-3.509032783

C	1.310229491	2.307805704	-2.698378892
C	2.621511988	2.319677287	-2.111821066
C	0.231084489	3.028438371	-2.019990591
C	0.496215137	3.720542013	-0.750074901
C	-0.587687765	3.903654414	0.160560271
C	-0.336374713	0.725102236	-3.675788049
C	-1.377169111	1.421458087	-3.070382662
C	-1.105580126	2.590905233	-2.275725719
C	-2.476087892	-0.694170949	-2.455593574
C	-1.377169111	-1.421458087	-3.070382662
C	-0.336374713	-0.725102236	-3.675788049
C	-2.476087892	0.694170949	-2.455593574
C	-1.865620287	-3.276705789	-0.061829768
C	-2.884136518	-1.404333843	-1.274390114
C	-2.118830043	-2.635198737	-1.253393131
C	-2.809068991	-0.713479997	2.406031378
C	-2.884136518	1.404333843	-1.274390114
C	-3.216149945	0.734673170	-0.062177057
C	-3.216149945	-0.734673170	-0.062177057
C	-2.976045792	-1.460306575	1.195107371
C	-2.362445477	-2.736293069	1.171701431
C	-2.118830043	2.635198737	-1.253393131
C	-1.865620287	3.276705789	-0.061829768
C	-2.362445477	2.736293069	1.171701431
C	-2.976045792	1.460306575	1.195107371
C	-2.809068991	0.713479997	2.406031378
Dy	2.450813684	0.000000000	-0.407516779
Dy	0.000000000	0.000000000	2.506103988
S	0.000000000	0.000000000	0.000000000

Table S23. $Dy_2S@C_{82}$-C_{3v}

C	1.511278523	-3.436464631	-1.617349998
C	2.811802690	-2.896760060	-1.458408777
C	3.453898835	-3.048941217	-0.193766215
C	3.155762071	-1.728444981	-2.231725810
C	2.127715915	-1.061372892	-2.990169395
C	0.783720029	-1.554572567	-3.099611541
C	0.533088527	-2.804344764	-2.471822565
C	4.167085383	-0.754369258	-1.824867129
C	4.756257109	-0.877814924	-0.483461079
C	4.354536511	-2.022311553	0.297759404
C	4.927077578	0.350293086	0.276463281
C	3.698798270	0.554922558	-2.330766936
C	2.797871258	2.742735434	-1.648833397
C	3.837711735	1.767251778	-1.535567986
C	4.479791118	1.644115478	-0.244683815
C	-1.612498967	-1.011377836	-2.872495529
C	-0.307179416	-0.594724532	-3.279205941
C	-0.007664046	0.845620038	-3.288249364
C	1.377725416	1.306314761	-3.129733126
C	2.435572316	0.334749292	-3.027781573
C	1.639013212	2.554830680	-2.503951200
C	4.686291828	0.377687536	1.710678024
C	4.317666607	-0.782576234	2.465044728
C	4.198694146	-1.999268367	1.743102890
C	3.502401606	-0.604318176	3.630777744
C	4.159176281	1.663952049	2.073870804
C	2.264632649	2.830793735	3.092837303
C	3.278174467	1.836331576	3.193260344
C	2.992715005	0.688852348	3.993531069
C	2.379229402	3.569193837	-0.531395845
C	2.942863869	3.386003810	0.755371160
C	4.004940285	2.429948661	0.873913385
C	2.090558593	3.590166805	1.882675822
C	2.523435657	-1.587607241	4.003004338
C	2.342018484	-2.769337246	3.219187220
C	3.231175720	-2.992322182	2.120744536
C	1.006882244	-3.266643946	3.119024434
C	1.412272287	-0.913228296	4.638023400
C	-1.033084277	-0.433952612	4.566435287
C	0.057751765	-1.403864564	4.547898520
C	-0.102492857	-2.572623539	3.723946002
C	0.969526967	2.633076636	3.693281183
C	0.658192247	1.486620722	4.499081495
C	1.706870217	0.509954551	4.624427758
C	-0.720040779	1.025122151	4.530266512
C	0.542242936	-3.904307221	1.915649125
C	1.409551913	-4.070578655	0.792364138
C	2.768478955	-3.634508042	0.919204600
C	0.811969706	-4.019718689	-0.493383977
C	-0.848752658	-3.588992682	1.746315396
C	-1.433802121	-3.435571093	0.455591328
C	-0.597112366	-3.762241733	-0.653277536
C	-1.807137741	-2.222611808	-2.105923165
C	-0.756667944	-3.095241415	-1.913722023
C	-3.542552265	-0.366633262	1.140476665
C	-2.933646679	-1.627889040	1.421380679

C	-2.518302648	-2.467287745	0.285163861
C	-2.748810339	-1.975214149	-1.037202719
C	-1.251510052	-2.766266344	2.846420671
C	-2.152204581	-0.646826113	3.638344278
C	-2.253387870	-1.772927828	2.718242226
C	-3.392706955	1.544708035	-0.209038059
C	-3.690521916	0.120661056	-0.203479278
C	-3.294106384	-0.668786785	-1.271455777
C	-2.721384281	2.107850314	-1.282908403
C	-2.581123225	0.644388926	3.166922477
C	-3.059527567	1.940679253	1.132045626
C	-3.279087884	0.786619796	1.968347521
C	-0.007197691	3.257983113	2.820315103
C	-1.701635585	3.093748218	-1.057650694
C	-1.292502266	3.462459728	0.260355273
C	-2.000023758	2.861218694	1.400890561
C	-1.314370785	2.731341606	2.690960469
C	-1.659336733	1.654848247	3.605772326
C	-0.739672924	2.938998918	-2.124804593
C	0.572532463	3.331503559	-1.944149422
C	0.985311592	3.889282349	-0.690252213
C	0.088797233	3.923907697	0.423969677
C	0.686105550	3.846720479	1.712848588
C	-2.597697334	-0.075078427	-2.405193748
C	-2.318572574	1.279832232	-2.411323610
C	-1.040842499	1.744783581	-2.880223797
Dy	2.489423403	0.000000000	-0.313895351
Dy	0.000000000	0.000000000	2.486167623
S	0.000000000	0.000000000	0.000000000

Table S24. $Dy_2C_2@C_{82}$-C_s

C	2.649498739	2.697722325	2.760318525
C	3.102522021	1.458698539	3.272870364
C	4.068255163	0.720136750	2.517031687
C	2.184210318	0.715888333	4.093525312
C	0.844717600	1.172411015	4.415418832
C	0.343323386	2.364269545	3.741740571
C	1.285261115	3.135852548	3.000418060
C	2.184210318	-0.715888333	4.093525312
C	3.102522021	-1.458698539	3.272870364
C	4.068255163	-0.720136750	2.517031687
C	2.649498739	-2.697722325	2.760318525
C	-1.360389265	0.000000000	4.254261838
C	0.022407600	0.000000000	4.704939813
C	0.844717600	-1.172411015	4.415418832
C	0.343323386	-2.364269545	3.741740571
C	1.285261115	-3.135852548	3.000418060
C	-1.427986334	3.158655194	2.177818549
C	-1.063384984	2.368341163	3.302824540
C	-1.860255021	1.171857118	3.539989367
C	3.038971125	-3.136626427	1.446588199
C	3.913902432	-2.348767317	0.649541409
C	4.465524582	-1.163378266	1.215650561
C	3.712487205	-2.349315195	-0.769583644
C	-0.445040948	-3.916516262	1.444924624
C	0.884578266	-3.891448771	1.844668573
C	1.921071145	-3.843734432	0.855625382
C	1.605653126	-3.718012391	-0.527555555
C	2.560021763	-3.006766084	-1.326952936
C	-1.860255021	-1.171857118	3.539989367
C	-1.063384984	-2.368341163	3.302824540
C	-1.427986334	-3.158655194	2.177818549
C	4.102060837	-1.247364535	-1.640343241
C	4.585442567	0.000000000	-1.047746080
C	4.722683328	0.000000000	0.384577789
C	4.102060837	1.247364535	-1.640343241
C	3.141578189	-1.241060136	-2.751949208
C	1.317453143	0.000000000	-3.814970520
C	2.655916668	0.000000000	-3.327407873
C	3.141578189	1.241060136	-2.751949208
C	0.833799023	-2.312606029	-2.980887250
C	2.195753268	-2.323645168	-2.524104749
C	0.461938244	-1.177226419	-3.755762748
C	-0.173605899	-3.037854508	-2.203884347
C	-0.778469410	-3.913456252	0.046698839
C	0.212085609	-3.733619229	-0.965294308
C	-1.526604343	-2.595687479	-2.330295179
C	3.712487205	2.349315195	-0.769583644
C	3.913902432	2.348767317	0.649541409
C	4.465524582	1.163378266	1.215650561
C	3.038971125	3.136626427	1.446588199
C	2.560021763	3.006766084	-1.326952936
C	1.605653126	3.718012391	-0.527555555
C	1.921071145	3.843734432	0.855625382
C	-0.445040948	3.916516262	1.444924624
C	0.884578266	3.891448771	1.844668573
C	0.461938244	1.177226419	-3.755762748

C	0.833799023	2.312606029	-2.980887250
C	2.195753268	2.323645168	-2.524104749
C	-0.173605899	3.037854508	-2.203884347
C	0.212085609	3.733619229	-0.965294308
C	-0.778469410	3.913456252	0.046698839
C	-0.896505029	0.725404541	-3.796086329
C	-1.873134231	1.424350468	-3.094575402
C	-1.526604343	2.595687479	-2.330295179
C	-2.903207027	-0.694476408	-2.375621149
C	-1.873134231	-1.424350468	-3.094575402
C	-0.896505029	-0.725404541	-3.796086329
C	-2.903207027	0.694476408	-2.375621149
C	-2.069203403	-3.282091344	-0.052351422
C	-3.191385625	-1.403940115	-1.159735877
C	-2.433506663	-2.637569664	-1.213148151
C	-2.762936769	-0.713070868	2.494162801
C	-3.191385625	1.403940115	-1.159735877
C	-3.400103930	0.733674068	0.077519827
C	-3.400103930	-0.733674068	0.077519827
C	-3.042832177	-1.460051953	1.304063625
C	-2.442483346	-2.738623876	1.222751116
C	-2.433506663	2.637569664	-1.213148151
C	-2.069203403	3.282091344	-0.052351422
C	-2.442483346	2.738623876	1.222751116
C	-3.042832177	1.460051953	1.304063625
C	-2.762936769	0.713070868	2.494162801
Dy	2.143759007	0.000000000	-0.868606301
Dy	0.000000000	0.000000000	2.329099616
C	0.000000000	0.633376212	0.000000000
C	0.000000000	-0.633376212	0.000000000

Table S25. $Dy_2ScN@C_{80}$-I_h

C	0.823892060	2.552254810	3.130909700
C	0.443322500	1.422750850	3.945148030
C	-0.980361510	1.098621310	4.018665860
C	1.375994970	0.310778340	3.966231380
C	2.074000600	2.612540420	2.398295340
C	3.678246790	1.256734190	1.148488580
C	2.940909050	1.494049530	2.365694370
C	2.588728940	0.361615490	3.163501140
C	0.916685710	-1.085662470	3.974109130
C	-0.499121170	-1.424014280	3.968641080
C	-1.451821250	-0.309696500	4.033457930
C	-0.860318870	-2.541831610	3.127444200
C	1.859691920	-1.842949120	3.162443500
C	2.228442790	-3.252493630	1.180298040
C	1.479616980	-2.973173520	2.367542500
C	0.104757980	-3.336876780	2.391736200
C	4.058107790	-0.058286770	0.709609020
C	3.596624330	-1.198413300	1.470054890
C	2.898076100	-0.961141910	2.702217380
C	3.273247940	-2.370246110	0.743114550
C	-2.102844390	-2.612177990	2.377569960
C	-2.975640060	-1.495412060	2.337904810
C	-2.619177550	-0.361327200	3.157396560
C	-3.713361570	-1.264948120	1.128459900
C	-1.903250030	-3.439767930	1.219275020
C	-1.890782070	-3.421141170	-1.233197600
C	-2.576016270	-3.155790940	-0.013347150
C	-3.523428540	-2.088040900	-0.034507040
C	1.583054540	-3.781317790	0.008197190
C	0.183045630	-4.065904190	0.001950290
C	-0.536458370	-3.881977830	1.225422120
C	-0.527683380	-3.876105590	-1.226539290
C	-4.034772690	0.059762700	0.681580660
C	-3.600270540	1.195108200	1.426726290
C	-2.904340430	0.959549650	2.666781440
C	-3.274248340	2.366214440	0.698146000
C	-4.078895520	0.062401190	-0.767107310
C	-3.060919440	1.002602900	-2.826529430
C	-3.689365140	1.224199600	-1.525094300
C	-3.279042240	2.365439050	-0.749704990
C	-2.101164720	-2.598265990	-2.409255420
C	-3.075829450	-1.533981010	-2.471933080
C	-3.768941770	-1.288161010	-1.223847230
C	-2.759448590	-0.368963020	-3.315027920
C	-2.234658030	3.250232480	1.153980420
C	-1.501107310	2.981974630	2.351445120
C	-1.887005840	1.845264710	3.153912720
C	-0.129024730	3.352850100	2.389208490
C	-1.584772760	3.799934460	-0.005768560
C	0.533846420	3.896642610	-1.219723330
C	-0.184757670	4.079800860	0.002213830
C	0.525297760	3.896014610	1.229970300
C	-1.918311810	1.847452480	-3.156700670
C	-1.490259750	2.980700430	-2.362029660
C	-2.224609690	3.257273090	-1.175304340

C	-0.109117580	3.344149960	-2.384705860
C	1.898068770	3.449613820	-1.210670330
C	2.573942560	3.169980380	0.009517720
C	1.889520930	3.439516310	1.234074130
C	3.537801670	2.092585690	-0.010581940
C	2.113859320	2.612199710	-2.371233090
C	2.674727840	0.370950000	-3.140304060
C	3.066252530	1.536097490	-2.386151010
C	3.895081180	1.324532760	-1.197621670
C	-0.934353160	1.051917640	-3.837842030
C	1.406864550	0.306994910	-3.842506600
C	0.473169470	1.381726660	-3.796250100
C	0.860309970	2.552692070	-3.093545770
C	-0.831634870	-2.551499920	-3.112777800
C	0.951908350	-1.054532190	-3.842616980
C	-0.445890070	-1.381373410	-3.822087740
C	-1.417166970	-0.306943900	-3.905405720
C	2.235210050	-3.235353940	-1.151436450
C	1.914113140	-1.840951060	-3.116873940
C	1.506254310	-2.970920430	-2.346663560
C	0.126558600	-3.344853380	-2.389860030
C	4.239726730	-0.049429870	-0.741057680
C	2.997248830	-0.976347520	-2.691489830
C	3.739744560	-1.219253800	-1.473860590
C	3.291524640	-2.353084290	-0.708662860
N	0.203394520	0.040389550	-0.070729020
Sc	2.020219140	0.177221560	-0.713553180
Dy	-0.184945690	-0.002866800	2.003388780
Dy	-1.467782580	-0.055418830	-1.350608720

Table S26. $Dy_2TiC@C_{80}$-I_h

C	2.685727640	2.011637430	2.280834260
C	3.371109440	0.743478430	2.328897290
C	4.006358020	0.337912870	1.090066620
C	2.774108190	-0.311590490	3.156063680
C	1.450098090	2.274668160	2.995298880
C	-0.639098730	1.256521660	3.742818860
C	0.792708380	1.231385730	3.698178050
C	1.466884380	-0.047847380	3.748033680
C	2.748011880	-1.719755790	2.684443920
C	3.320802980	-2.096014460	1.392689520
C	2.625102550	-3.096662180	0.619641050
C	1.435716180	-2.259596960	3.021081460
C	-0.685988750	-3.274648860	2.276122660
C	0.741297300	-3.259961130	2.241161090
C	1.383579260	-3.706480750	1.052499610
C	-1.410001250	0.044053710	3.747550230
C	-0.766647760	-1.227915700	3.683989190
C	0.674996030	-1.248635480	3.698600270
C	-1.431517320	-2.273172950	2.993883930
C	2.608206580	-3.094192200	-0.828124750
C	3.204482560	-2.031016700	-1.547962450
C	3.917033530	-1.034567750	-0.817131150
C	2.554522800	-1.618498030	-2.767813120
C	1.375777800	-3.697399090	-1.274485510
C	-0.711252750	-3.296914170	-2.487603500
C	0.710473850	-3.262023350	-2.457162860
C	1.354073050	-2.236686290	-3.256504800
C	-1.454235250	-3.649835420	1.118629770
C	-0.808631500	-4.001664110	-0.103707870
C	0.620278820	-4.072824930	-0.108439930
C	-1.466967140	-3.654394140	-1.326255320
C	2.602430570	-0.271747150	-3.266193660
C	3.244862700	0.746791180	-2.455548650
C	3.917798590	0.332594490	-1.261272060
C	2.670751730	2.041448860	-2.485368150
C	1.467053680	-0.044816050	-4.161112650
C	-0.650430460	1.262598060	-3.957494880
C	0.794043440	1.251404490	-4.001741990
C	1.426809910	2.262878280	-3.204267160
C	-1.441516680	-2.266678160	-3.202117290
C	-0.797070280	-1.258192680	-3.992224160
C	0.663517690	-1.306011630	-4.147444540
C	-1.425096290	0.039405250	-3.959222050
C	2.681244790	2.889257400	-1.331437950
C	3.274796700	2.444070050	-0.107651950
C	3.939854650	1.176161070	-0.095563060
C	2.678751280	2.869383270	1.113345310
C	1.449452950	3.639097100	-1.324984760
C	-0.628296480	4.046891980	-0.098086800
C	0.805565930	3.991356630	-0.101791760
C	1.464859550	3.642939220	1.118503400
C	-1.403934370	2.256246390	-3.222185240
C	-0.744873110	3.256976620	-2.453575070
C	0.682127790	3.265286680	-2.481398280
C	-1.394605200	3.695243580	-1.258565510

C	-1.388334020	3.683265760	1.065824280
C	-0.722824970	3.247623960	2.252502310
C	0.708925530	3.281409690	2.285043520
C	-1.387731910	2.253656360	3.025037810
C	-2.658199630	3.117595970	0.641637240
C	-4.119992920	1.094569670	0.668031940
C	-3.365266890	2.115971650	1.416830810
C	-2.649742650	1.674513030	2.593407620
C	-2.643092260	1.662777930	-2.787011980
C	-4.043755750	1.076873340	-0.810109090
C	-3.282792220	2.066203190	-1.558452540
C	-2.640380760	3.108671980	-0.815343870
C	-2.667074240	-2.013586670	-2.474631440
C	-3.944077970	-0.307281650	-1.242757500
C	-3.265737930	-0.721794150	-2.452262330
C	-2.654041630	0.295371910	-3.232742550
C	-2.673680870	-2.882590580	1.122905000
C	-3.928624220	-1.151145220	-0.081870880
C	-3.263097030	-2.434194730	-0.092111940
C	-2.673762130	-2.864098800	-1.317350890
C	-2.639529840	0.290496450	3.040207170
C	-4.010838520	-0.311768840	1.093231200
C	-3.270518170	-0.732865680	2.268360820
C	-2.665222400	-2.029510740	2.283166950
C	3.983017140	-1.053988510	0.634307290
C	0.038229750	-0.194354510	-0.502107710
Dy	-1.841034590	0.643332070	0.207456970
Dy	1.538777230	-0.361237530	1.088188460
Ti	0.405295960	-0.243860050	-2.293654360

Table S27. $Dy_2TiC_2@C_{80}$-I_h

C	-0.250448780	-3.110300320	-2.677179690
C	-0.282759580	-3.817577000	-1.433445900
C	-1.502218690	-3.754606880	-0.696639890
C	0.935072380	-3.953387280	-0.701823590
C	0.956110830	-2.472614810	-3.119151500
C	3.008911750	-1.364806910	-2.360294250
C	2.151176360	-2.522075690	-2.335877630
C	2.142777340	-3.306458970	-1.152938550
C	0.938257440	-3.928390840	0.737257530
C	-0.271745550	-3.777132550	1.466358670
C	-0.214224650	-3.025088700	2.697407250
C	2.154718260	-3.287872020	1.176026000
C	3.075061210	-1.371394780	2.370996770
C	2.198719020	-2.498830610	2.354170590
C	0.995389910	-2.414843040	3.164376430
C	3.813940830	-0.973771890	-1.240069930
C	3.704898980	-1.727075870	-0.008483730
C	2.904977020	-2.905841270	0.000679180
C	3.813441510	-0.988888180	1.204931540
C	-1.328217130	-2.259393840	3.207436820
C	-2.517974560	-2.155878930	2.372607810
C	-2.607523630	-2.949039060	1.190857520
C	-3.198722630	-0.908734310	2.376454890
C	-0.834559530	-1.196138920	4.105466310
C	-0.630319250	1.316054610	3.863739970
C	-1.470605640	0.134892930	3.928835880
C	-2.640366790	0.224176630	3.093956340
C	2.683341600	-0.171637200	3.068604400
C	1.468396280	-0.083227160	3.841926670
C	0.656002790	-1.286704160	4.037661820
C	0.810966600	1.198307520	3.824478700
C	-3.893007490	-0.418484940	1.224241160
C	-3.914200310	-1.172243940	0.008197040
C	-3.294098090	-2.451871260	0.025400120
C	-3.946934150	-0.454088040	-1.252685040
C	-3.774764120	1.019076090	1.217462450
C	-2.910741970	2.934048940	-0.022196720
C	-3.687991170	1.741641180	-0.010482240
C	-3.795032050	0.985567460	-1.240661390
C	-0.984763610	2.508604120	3.118717130
C	-2.177001830	2.571745100	2.336182570
C	-3.016608750	1.419571380	2.365717120
C	-2.160031620	3.345065370	1.135414100
C	-3.382479610	-0.983305540	-2.495622170
C	-2.613454290	-2.236208320	-2.402252710
C	-2.618856730	-2.950644320	-1.154127100
C	-1.377470990	-2.329366520	-3.146299740
C	-2.839205570	0.176720230	-3.247131570
C	-0.665167040	1.237377580	-3.867315240
C	-1.529853580	0.082629580	-3.900548440
C	-0.848461460	-1.187445580	-3.871403680
C	-2.163514690	3.303900120	-1.198550920
C	-2.200014470	2.517640960	-2.384044920
C	-3.097293000	1.374403180	-2.438692720
C	-1.004600400	2.453889730	-3.167564080

C	0.775705650	1.132717120	-3.857289410
C	1.429114960	-0.126502530	-3.813219230
C	0.599397540	-1.286939820	-3.844593290
C	2.689806390	-0.185593600	-3.116654620
C	1.330412780	2.272819330	-3.163110560
C	2.664753710	3.009466910	-1.248755210
C	2.600188710	2.234059910	-2.471336870
C	3.343544810	0.968708670	-2.503447620
C	-0.943897890	3.942825310	-0.764131630
C	1.504321400	3.751016170	-0.780305350
C	0.263440330	3.797298550	-1.502816480
C	0.209707180	3.084912420	-2.736155550
C	0.226316680	3.124977460	2.655538510
C	1.496593850	3.743920820	0.651547090
C	0.274323750	3.816061140	1.403014120
C	-0.940735630	3.958559630	0.674523900
C	3.200763740	0.951888720	2.334394930
C	2.619357140	2.964022240	1.098330970
C	2.519750040	2.199910820	2.306028100
C	1.336692720	2.324946850	3.090825930
C	4.061688830	0.466764460	-1.309212530
C	3.373235530	2.513520120	-0.058755730
C	4.040332150	1.222345230	-0.050110230
C	3.903592110	0.459909030	1.168545630
C	-1.499003780	-3.748446790	0.738021070
Ti	-0.194160260	-0.521568080	2.235291900
Dy	-1.421008860	-0.268177750	-1.410180480
Dy	1.749097460	0.708675850	-0.737634440
C	-0.274301200	0.556367900	0.540315140
C	0.139885640	-0.719736240	0.267099550

Table S28. $Dy_2O@C_{82}$-C_{3v}

C	-3.163523370	2.664891930	-0.371310770
C	-2.369805140	3.050286720	-1.482082530
C	-2.391819480	2.217060140	-2.639098110
C	-1.157083230	3.793632650	-1.223636540
C	-0.710273080	3.946202470	0.139852910
C	-1.464081590	3.481609260	1.271441220
C	-2.742172340	2.933444680	0.983757690
C	0.004471590	3.784209190	-2.110317220
C	0.000777930	2.832168490	-3.228395450
C	-1.203699620	2.075865760	-3.458134030
C	1.225764840	2.108398520	-3.492073770
C	1.192353590	3.908361840	-1.239453660
C	3.150056740	2.683062400	-0.360576830
C	2.396418140	3.127705520	-1.492387380
C	2.401534080	2.260189280	-2.644312680
C	-1.420765810	2.169638180	3.362238140
C	-0.745980750	3.063164310	2.476035410
C	0.722305170	3.067867370	2.478280890
C	1.454645240	3.503545540	1.282350110
C	0.721114330	3.990011920	0.140164590
C	2.731796350	2.949021730	1.002616540
C	1.215578440	0.733825160	-3.957187270
C	0.003428500	0.011978470	-4.194197660
C	-1.211302490	0.719265780	-3.972673540
C	0.007992310	-1.402475550	-3.981272470
C	2.376029600	0.061478970	-3.439489070
C	3.122759970	-1.790602590	-2.022324360
C	2.357874790	-1.339432460	-3.138576060
C	1.172681660	-2.068426580	-3.460078580
C	3.870793920	1.424193950	-0.352143490
C	3.808768240	0.541379600	-1.460118670
C	3.092158400	0.990745180	-2.615764500
C	3.832423270	-0.859044760	-1.185443680
C	-1.155632790	-2.069825130	-3.463189410
C	-2.348920800	-1.346139280	-3.162045860
C	-2.382500020	0.048079510	-3.478429960
C	-3.109071560	-1.801405220	-2.039413790
C	-0.716114010	-3.166454050	-2.634520420
C	-0.749172030	-4.246723380	-0.387047650
C	-1.461608650	-3.635738760	-1.498578550
C	-2.656003330	-2.890438630	-1.214242400
C	2.665584380	-2.874904530	-1.194474070
C	1.458591770	-3.589603540	-1.478792860
C	0.730163320	-3.159321480	-2.634601220
C	0.730950780	-4.191679320	-0.380462730
C	-3.823044020	-0.872722310	-1.203609660
C	-3.809512080	0.528092590	-1.484335410
C	-3.106089150	0.974672420	-2.649075080
C	-3.878907170	1.407515740	-0.372881280
C	-3.790379840	-1.363748680	0.145728460
C	-3.760121750	-0.486077630	1.264848590
C	-3.912585590	0.903258360	0.977017930
C	-2.647241660	1.516569970	2.968241260
C	-3.289195900	1.892481060	1.806634080
C	-1.186928770	-2.319382980	3.360281040

C	-2.293532600	-2.182185230	2.464924570
C	-3.027988030	-0.904682330	2.461527660
C	-2.595585390	0.124181130	3.352903950
C	-3.068466950	-2.603054900	0.150299470
C	-1.198143240	-3.949330980	0.982621770
C	-2.307876060	-3.043192250	1.267315460
C	0.714699160	-1.228393150	4.185534080
C	-0.738979500	-1.231238320	4.185658530
C	-1.429755510	-0.029544280	4.175577710
C	1.404278370	-0.025644170	4.182292650
C	-0.011652430	-3.825515020	1.800674910
C	1.166556640	-2.314076300	3.358373410
C	-0.008509300	-3.056938630	2.968995730
C	3.073228520	-2.590567900	0.164944600
C	2.575507460	0.133175280	3.366350930
C	3.014556050	-0.893627940	2.477240420
C	2.279624600	-2.167680900	2.472607260
C	2.289928830	-3.006434610	1.269729690
C	1.166400920	-3.882327510	0.974371730
C	2.624927250	1.525110440	2.981623460
C	3.275259020	1.906483780	1.823855890
C	3.902931540	0.920549710	0.996125380
C	3.750295680	-0.472832200	1.281420410
C	3.793945560	-1.350764850	0.165655010
C	-0.705781500	1.233019360	4.185160720
C	0.677751840	1.235209820	4.188591590
C	1.394066330	2.172908050	3.366298930
Dy	0.341337210	1.632143920	-1.075093080
Dy	-0.179997490	-1.925852690	-0.044625700
O	-0.012520600	0.080690620	0.202856590

Table S29. DyNC@C_{76}-C_s

C	3.589159381	-2.645193049	-0.706155510
C	3.589159381	-2.645193049	0.706155510
C	3.893000382	-1.430310496	-1.418774124
C	2.523536140	-3.300833823	-1.420320787
C	3.893000382	-1.430310496	1.418774124
C	2.523536140	-3.300833823	1.420320787
C	1.524822876	-3.974188854	-0.724052159
C	4.234957777	-0.274734336	-0.723455320
C	1.524822876	-3.974188854	0.724052159
C	4.234957777	-0.274734336	0.723455320
C	2.154824026	-2.480050721	-2.567297027
C	3.002616340	-1.323513575	-2.566979995
C	3.002616340	-1.323513575	2.566979995
C	2.154824026	-2.480050721	2.567297027
C	0.150370463	-3.916891666	-1.170431402
C	3.773339605	1.021396714	-1.170011217
C	3.773339605	1.021396714	1.170011217
C	0.150370463	-3.916891666	1.170431402
C	0.810348364	-2.417489042	-3.031948384
C	2.543310152	-0.057739690	-3.031726662
C	0.810348364	-2.417489042	3.031948384
C	2.543310152	-0.057739690	3.031726662
C	-0.694504272	-3.948246838	0.000000000
C	3.552802086	1.838430701	0.000000000
C	-0.225915501	-3.176790686	-2.320594124
C	2.957066886	1.159451727	-2.321031107
C	-0.225915501	-3.176790686	2.320594124
C	2.957066886	1.159451727	2.321031107
C	1.283913344	-0.065626028	-3.700440143
C	0.442409024	-1.212148918	-3.701064858
C	0.442409024	-1.212148918	3.701064858
C	1.283913344	-0.065626028	3.700440143
C	-1.983375962	-3.343926627	0.000000000
C	2.592589400	2.889174494	0.000000000
C	-1.573978078	-2.688538086	-2.355117464
C	2.089602444	2.300883445	-2.354669263
C	-1.573978078	-2.688538086	2.355117464
C	2.089602444	2.300883445	2.354669263
C	0.417588753	1.108393921	-3.694820078
C	-0.936185449	-0.735154135	-3.695648688
C	0.417588753	1.108393921	3.694820078
C	-0.936185449	-0.735154135	3.695648688
C	-2.438821331	-2.773041565	-1.224825401
C	1.912830748	3.153139860	-1.225523925
C	-2.438821331	-2.773041565	1.224825401
C	1.912830748	3.153139860	1.225523925
C	0.828008732	2.261961886	-3.051858388
C	-1.913677775	-1.472378554	-3.051985907
C	0.828008732	2.261961886	3.051858388
C	-1.913677775	-1.472378554	3.051985907
C	-0.958328523	0.699696129	-3.663965230
C	-0.958328523	0.699696129	3.663965230
C	-3.317077909	-1.613067011	-1.223820751
C	0.544274485	3.646519508	-1.225164080
C	-3.317077909	-1.613067011	1.223820751

C	0.544274485	3.646519508	1.225164080
C	-2.970584890	-0.789453348	-2.350844613
C	-0.138404995	3.068762369	-2.351799495
C	-2.970584890	-0.789453348	2.350844613
C	-0.138404995	3.068762369	2.351799495
C	-1.946225955	1.426067667	-2.917417865
C	-1.946225955	1.426067667	2.917417865
C	-3.749272760	-1.026938002	0.000000000
C	-0.141996593	3.881330452	0.000000000
C	-1.524682101	2.699308565	-2.301790756
C	-3.035905995	0.644116412	-2.300386137
C	-1.524682101	2.699308565	2.301790756
C	-3.035905995	0.644116412	2.300386137
C	-1.573628506	3.711431262	0.000000000
C	-4.024278917	0.388246821	0.000000000
C	-2.277082704	3.221360441	-1.182784574
C	-3.766394382	1.205142573	-1.184135822
C	-2.277082704	3.221360441	1.182784574
C	-3.766394382	1.205142573	1.184135822
C	-3.457514077	2.533769815	-0.722820297
C	-3.457514077	2.533769815	0.722820297
Dy	-1.571014677	1.145421350	0.000000000
N	0.504332804	0.260746230	0.000000000
C	0.812972953	-0.881058199	0.000000000

Table S30. DyNC@C_{82}-C_2

C	3.162031339	-1.945913501	1.862128257
C	3.988699647	-0.956903017	1.266029601
C	3.966593274	0.422897746	1.729592535
C	3.233601945	0.801688885	2.880318896
C	2.561183570	-0.236237558	3.608664279
C	2.510747839	-1.567677223	3.081072966
C	1.390616406	-0.008553904	4.430771746
C	1.383890905	2.310834797	3.591259692
C	2.649526039	2.097052607	2.902134152
C	0.606421068	-1.245560658	4.408837195
C	1.303065425	-2.177451691	3.534025951
C	2.822806874	-2.956148104	-0.351344938
C	-0.698756540	1.312540128	4.371501630
C	-1.408732214	2.229523117	3.492744406
C	0.749180503	1.300765507	4.373149918
C	-0.844012631	-1.228566968	4.403409499
C	-1.487200628	-2.257130980	3.654649802
C	-0.757046417	-3.272661814	2.911647864
C	0.652038101	-3.185065649	2.743763047
C	1.269579787	-3.596022664	1.485093439
C	2.533642398	-2.981373300	1.042618900
C	0.396727309	-4.128783506	0.493008762
C	-1.031824668	-4.117947096	0.647943601
C	-1.607464897	-3.711116332	1.843798638
C	-2.822706966	-2.934653781	1.845956802
C	4.050682536	1.295070084	0.586700902
C	-1.480883426	0.078953364	4.424847540
C	-3.341938076	-0.762221380	2.926089175
C	4.192758711	-0.926548571	-0.151745269
C	4.202170249	0.460774402	-0.580773028
C	3.315537009	2.514305621	0.535176968
C	3.607438449	-1.905977758	-0.946122040
C	2.703475204	2.948240893	1.755660605
C	0.646066329	-3.903822342	-0.907156664
C	1.815907801	-3.322381985	-1.321058452
C	-2.665361687	0.291550240	3.622718638
C	-3.282771916	1.961420244	1.847959096
C	-0.592330853	-3.566379213	-1.564124019
C	-2.757282297	-2.056912307	2.972556248
C	-2.823120342	-2.939486622	-0.645349631
C	-1.646947422	-3.739231984	-0.609350891
C	-4.086871055	-0.410267053	1.773819529
C	-0.764497124	3.217673745	2.677250138
C	-2.623366488	1.613097907	3.070392717
C	-0.770816776	1.737872025	-2.949444395
C	-2.962105793	2.920551935	-0.391883160
C	1.738094578	0.358254244	-3.125873872
C	-4.114167245	0.958720266	1.280314172
C	2.981139209	-1.531678428	-2.202354460
C	-3.108199980	0.110882186	-2.568586881
C	1.689912460	1.754227084	-2.723854500
C	-1.849192480	-1.842244668	-2.668704926
C	-2.953979121	-2.088873460	-1.783436984
C	0.901471925	4.103812578	0.547710376
C	-4.329451307	0.896312131	-0.134757414

C	1.487110073	3.723308893	1.747263381
C	-3.740011654	-0.881852036	-1.726666964
C	-4.180476616	-1.308024925	0.651934582
C	0.609926523	-1.831441752	-2.915835684
C	0.558561882	-0.407810067	-3.277375226
C	-3.135972945	1.454398261	-2.208994527
C	-1.982922208	2.269530876	-2.435823926
C	0.646622976	3.308763068	2.832119400
C	-1.901550695	-0.455789676	-3.102186645
C	-0.722803365	0.306498450	-3.280150950
C	2.802733874	2.021031193	-1.854668029
C	-2.661155529	2.977695341	0.998715766
C	-1.393003923	3.602039202	1.415484496
C	-0.528656062	4.111971549	0.404580956
C	-1.963240161	3.264320881	-1.377845156
C	2.949331570	-0.196368897	-2.590592662
C	0.442852879	3.502504375	-1.647260500
C	3.589000650	0.815346265	-1.777294075
C	1.505733288	3.696841252	-0.706024887
C	2.682227876	2.897192885	-0.735039936
C	0.465014989	2.438883343	-2.597877033
C	-0.789969632	3.855420162	-0.987662997
C	-3.751905842	1.857473538	-0.956285462
C	-0.622891076	-2.523912365	-2.537528996
C	1.826362738	-2.351860544	-2.401342157
C	-3.445642269	-2.528450090	0.621857771
C	-4.341936763	-0.500470099	-0.532676796
C	-0.586971416	0.000000000	-0.076666934
N	0.586971416	0.000000000	0.076666934
Dy	0.000000000	0.000000000	2.382968342
